# Multifunctional injectable hydrogel system as a mild photothermal-assisted therapeutic platform for programmed regulation of inflammation and osteo-microenvironment for enhanced healing of diabetic bone defects *in situ*

**DOI:** 10.7150/thno.102779

**Published:** 2024-10-28

**Authors:** Yufan Zhu, Huifan Liu, Ping Wu, Yun Chen, Zhouming Deng, Lin Cai, Minhao Wu

**Affiliations:** 1 Department of Spine Surgery and Musculoskeletal Tumor, Zhongnan Hospital of Wuhan University, 168 Donghu Street, Wuchang District, Wuhan 430071 Hubei, China.; 2 Department of Anesthesiology, Research Centre of Anesthesiology and Critical Care Medicine, Zhongnan Hospital of Wuhan University, Wuhan, Hubei, China.; 3 National Key laboratory of macromolecular drug development and manufacturing, School of Pharmaceutical Science, Wenzhou Medical University, Wenzhou, 325035, China.; 4 Department of Biomedical Engineering and Hubei Province Key Laboratory of Allergy and Immune Related Disease, TaiKang Medical School (School of Basic Medicine Sciences), Wuhan University, Wuhan 430071, China.

**Keywords:** multifunctional hydrogel, minimally invasive injection, mild photothermal therapy, immune regulation, bone regeneration

## Abstract

**Background:** Factor-free biomaterial scaffolds play an increasingly important role in promoting *in situ* bone reconstruction and regeneration. However, the complicated and variable pathophysiological microenvironments of the injury sites under diabetic conditions, including the vicious cycle of oxidative stress and inflammatory response, impaired osteo/angiogenesis function and hyperactive osteoclastogenesis, as well as increased susceptibility to bacterial infection, may largely weaken the therapeutic potential of implanted scaffolds, leading to uncontrolled and poor outcomes of bone defect healing.

**Methods and Results:** To tackle the aforementioned challenges, a mild photothermal-assisted multifunctional therapeutic platform (denoted as GAD/MC) that integrates copper-containing two-dimensional Ti_3_C_2_T_x_ MXene nanosheets, gelatin methacrylate, and alginate-graft-dopamine was proposed to achieve efficient and synergistic therapy for diabetic bone defects. Thereinto, copper-decorated MXene (MC) nanosheets were employed as both functional crosslinkers and nanofillers to participate in the construction of an interpenetrating polymer network structure through multiple covalent and noncovalent bonds, which conferred the hydrogel with advantageous traits like enhanced mechanical properties, injectability and moldability, strong bone tissue adhesion and self-healing ability, as well as excellent anti-swelling and near-infrared (NIR) photothermal conversion capabilities. On account of the NIR/pH dual-responsive properties, the resulting hydrogel system was capable of achieving the controlled and stimuli-responsive release of bioactive Cu^2+^, allowing on-demand delivery at the site of injury. Moreover, with the assistance of mild photothermal effects, this integrated hydrogel system demonstrated remarkable antibacterial and antioxidant properties. It effectively scavenged excessive reactive oxygen species (ROS), inhibited inflammatory responses, and promoted macrophage polarization towards the pro-healing M2 phenotype. Such characteristics were beneficial for recreating an optimized microenvironment that supported the adhesion, proliferation, migration, and differentiation of osteoblasts and endothelial cells, while concurrently inhibiting osteoclast function. In a critical-sized cranial defect model using diabetic rats, the injectable GAD/MC hydrogel system combined with on-demand mild hyperthermia further synergistically accelerated new bone formation and bone healing processes by eliminating intracellular ROS, ameliorating inflammation, orchestrating M2 macrophage polarization, promoting osteo/angiogenesis, and suppressing osteoclastogenesis.

**Conclusions:** Overall, the constructed multifunctional injectable hydrogel system has emerged as a promising therapeutic candidate for addressing complex bone-related challenges by remodeling the disordered immune microenvironment and expediting the bone healing process.

## Introduction

With the population increasing and aging, the incidence of orthopedic disorders such as bone fractures, osteoporosis, bone infection, tumors, and rheumatic diseases has encountered a huge surge in the past several years. In particular, large bone defects and bone destruction, alternatively referred to as critical-sized bone defects, caused by trauma or other diseases affect more than 20 million people annually worldwide, imposing a significant socioeconomic burden on individual patients and public health systems 1. Current research estimates that more than half a million patients receive bone grafting or bone reconstruction procedures in the United States, generating a yearly financial burden greater than $2.5 billion 2. Generally, natural bone possesses innate regenerative potential, but the physiological process of bone healing can be severely compromised by defect size and other comorbid conditions, such as osteoporosis, diabetes, wound infection, and periodontitis. Under these circumstances, the *in situ* regeneration of injured bone tissues, especially when repairing critical-sized bone defects, is heavily impeded by the pathological immune microenvironment, which includes disrupted immune homeostasis, impaired blood vessel formation and osteoblast function, increased osteoclast formation, and heightened inflammation 3. However, effectively and dynamically modulating these intricate pathological dilemmas and simultaneously restoring the regenerative microenvironment remain extremely challenging. For instance, diabetes mellitus (DM) is a common chronic metabolic disorder in China and around the world, and the global prevalence of diabetes is estimated to exceed 10% by 2030 on account of the increase in the aging and obese population, which has become a major global health issue 4. Notably, the regeneration of bone defects in diabetic individuals often involves delayed bone healing, accompanied by a high risk of bone nonunion and infection, ultimately giving rise to repair failure. This is primarily attributed to prolonged systemic hyperglycemia and chronic inflammation, as well as resulting cellular dysfunction, which exerts a particularly detrimental effect on tissue regeneration 5. Moreover, under diabetic inflammatory conditions, macrophages, as one of the most vital innate immune cells, are overly polarized into classically activated proinflammatory M1 macrophages, leading to increased generation of reactive oxygen species (ROS) and inflammatory cytokines at the wound/injury site, thus hampering the intrinsic healing process. Furthermore, this immune imbalance and uncontrolled ROS accumulation combined with exposed wounds are particularly susceptible to bacterial infection, which further exacerbates inflammatory reactions, oxidative stress, and tissue/cellular dysfunction in the bone defect microenvironment 6. These complex situations, including high levels of proinflammatory cytokines and ROS, also expand abnormal inflammation to the surrounding bone tissue accompanied by endothelial dysfunction and abnormal bone metabolism, thus delaying bone healing and increasing the incidence of complications (nonhealing, bone disconnection, etc.) 7. Unfortunately, there is currently no widely adaptable strategy for effectively reducing unfavorable inflammatory responses and simultaneously recreating a conducive regenerative microenvironment for bone defect healing in patients with DM. Crucially, treating diabetic bone defects is frequently compounded by persistent and chronic inflammation within the defective area, hyperglycemia, oxidative stress, compromised osteo/angiogenesis, enhanced osteoclast activity, and overproduction of proinflammatory and chemotactic cytokines. Therefore, there is an urgent need to develop effective and feasible strategies to address these pathological issues concurrently and restore the proper immune microenvironment for augmented bone repair.

Recently, the use of biodegradable bioactive scaffolds, such as microspheres, nanoparticles, foams, sponges, fibers, aerogels, hydrogels, and other hybrid materials, to regulate the local immune response and simultaneously promote tissue regeneration has become a prospective strategy for augmenting bone healing in the clinic. In particular, injectable hydrogels, as polymer materials with three-dimensional (3D) network structures, have good biocompatibility, degradability, and adaptable physicochemical properties and can be injected into defective areas via minimally invasive surgical methods to form 3D scaffolds *in situ* 8. These *in situ*-formed hydrogels are structurally similar to the natural extracellular matrix (ECM), which has been reported to simulate the microenvironment of cell growth and accelerate bone healing. Additionally, they are convenient for filling irregular or deep defects and can seamlessly integrate with host tissues, thus providing a suitable microenvironment for guiding cell function and tissue repair 9. Recent advancements have been made in the development of multifunctional hydrogels that can tackle various types of physiological and pathological bone repair. As a representative example, silk fibroin/gelatin hydrogel patches or conductive alginate/gelatin scaffolds containing polydopamine-mediated micromaterials have been adopted for diabetic periodontal tissue regeneration 10, 11. Additionally, multifunctional hydrogel implants with mild photothermal activity or controlled drug release systems have been reported to be beneficial for relieving inflammation, promoting osteo/angiogenesis, and preventing bacterial infection 12, 13. Among the various strategies for accelerating bone healing, mild photothermal therapy (PTT), an imperative biophysical-level regulator and nonpharmacological intervention, has garnered widespread attention and has been widely studied due to its minimal side effects, relative safety, high efficiency, and spatiotemporal precision 14. In the past few decades, great progress has also been made in accelerating neovascularization and tissue regeneration to repair large bone defects via mild PTT in animal models 15. Recently, researchers have shown that mild hyperthermia therapy (~45 °C) induced by near-infrared (NIR) light irradiation can accelerate cell biomineralization and endogenous bone repair through the upregulation of heat shock proteins (HSPs), such as HSP47 and HSP70 16. Thus, to efficiently repair bone defects in the diabetic inflammatory state, NIR-induced mild PTT is a potentially remarkable assistant for coordinating inflammation and tissue regeneration. More importantly, the combined application of mild PTT with bioactive ingredients (e.g., drugs, cytokines, small molecules, extracellular vesicles, growth factors, and metal ions) and photothermal conversion agents can significantly amplify the therapeutic effect of biomaterial-mediated immunomodulation and tissue regeneration, offering innovative approaches and strategies to promote bone healing 17. With these findings in mind, we envision that mild hyperthermia-assisted bioactive hydrogels might synergistically modulate abnormal inflammation and tissue regeneration in diabetic bone defects, thus restoring bone homeostasis and resolving DM-related pathological conditions.

However, whether this photoactivated hydrogel therapeutic platform is able to provide on-demand thermal cues for enhanced *in situ* bone defect repair under comorbidity conditions (e.g., diabetes) is unclear. Previous studies have documented that multifunctional and ECM-mimicking biomaterials, especially injectable, adhesive, and self-healing hydrogels with the ability to scavenge inflammatory mediators, mitigate oxidative stress, and promote osteogenesis and vascularization, are potentially valuable therapeutic platforms for long-term immunomodulation and efficient tissue regeneration 18. However, in most works, hydrogels fabricated for bone regeneration lack adequate shape-adaptive and adhesive properties, which are required to repair irregular bone defects and multiple fragments in complex bone fractures 19. In addition, these hydrogels easily moved away from the defect region, which was closely related to the failure of bone repair. More importantly, in the presence of weak noncovalent interactions, the vast majority of available hydrogel materials are typically fragile and prone to breaking when subjected to external tension and exhibit uncontrolled swelling and biodegradation behaviors, thus limiting their clinical application prospects in the efficient induction of bone repair 20. Consequently, the coordinated manipulation of both physicochemical cues and the biological performance of preexisting hydrogels appears to be a rational and feasible strategy to meet these more rigorous requirements of bone repair in pathological microenvironments; however, this approach remains a formidable challenge in the fields of bone tissue engineering and regenerative medicine. Overall, the *in situ* regeneration of damaged bone tissues under comorbidity conditions (e.g., diabetes) still presents various challenges, necessitating materials with excellent antibacterial and ROS-scavenging capacities, good anti-inflammatory and immunomodulatory properties, and osteo/angiogenesis-promoting effects to restore an instructive osteoimmune microenvironment. Importantly, these materials maintain injectability; shape-adaptive and bio-adhesive properties to fill irregular bone defects; appropriate mechanical strength and stability; and ECM-mimicking porous structures for efficient cell infiltration, growth, proliferation, migration, and subsequent differentiation.

**Scheme 1:**
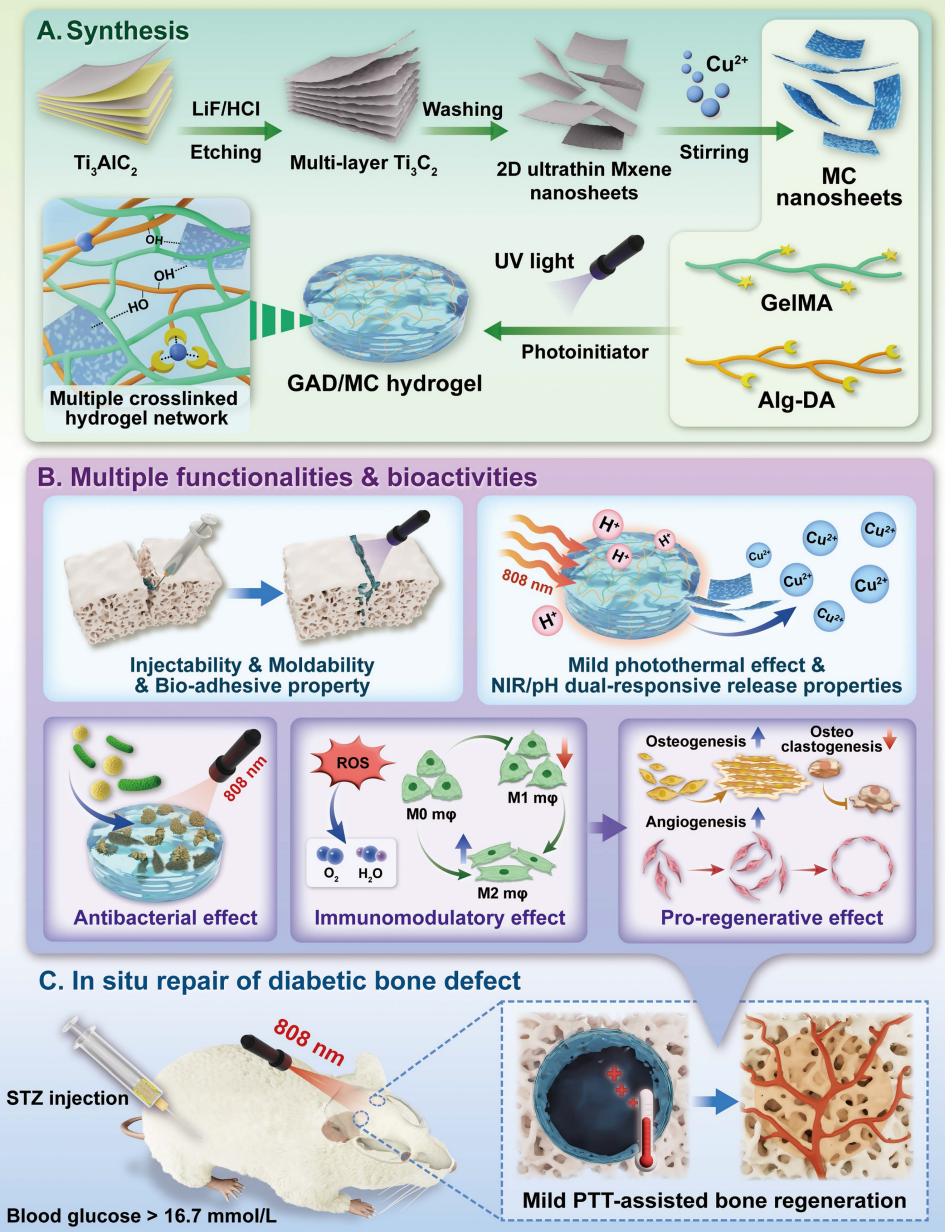
Schematic illustration of the **(A)** preparation and **(B)** application of the mild photothermal-assisted multifunctional injectable therapeutic system for **(C)** diabetes-related bone defect repair.

In the present study, we proposed a combined therapeutic strategy to synergistically restore the regenerative microenvironment and boost endogenous bone regeneration under inflammation-related pathological conditions on the basis of a multifunctional injectable hydrogel system (denoted as GAD/MC), along with adjunct near-infrared (NIR)-mediated mild PTT, as illustrated in the **Scheme 1**. Specifically, we integrate copper (Cu)-functionalized two-dimensional (2D) Ti_3_C_2_T_x_ MXene (MC) nanosheets *in situ* into a gelatin methacrylate/alginate-graft-dopamine (GelMA/Alg-DA) hybrid hydrogel network and combine it with on-demand NIR-induced mild photothermal effects with the aim of accelerating diabetic bone defect healing through eliminating the overproduction of ROS, controlling bacterial infections, regulating the local immune microenvironment, and promoting osteo/angiogenesis while inhibiting osteoclastogenesis without the addition of growth factors. As a rising star on the horizon of 2D nanomaterials, Ti_3_C_2_T_x_ MXene nanosheets with considerable biocompatibility and biodegradability have received increasing attention in the fields of biomedical engineering, such as drug delivery, cancer therapy, and tissue regeneration 21. Compared with other 2D nanomaterials (e.g., graphene oxide) with high surface areas and photothermal properties, MXene nanosheets are biodegradable, which is crucial for tissue engineering, and the degradation products (e.g., Ti-based species) have the potential to direct the behavior of osteoblasts, including adhesion, proliferation, and further differentiation 22. Moreover, nanoscale MXenes with outstanding photothermal conversion ability for NIR light could lead to high-quality repair of bone defects through NIR-assisted mild hyperthermia therapy 23. However, for the treatment of diabetic bone defects, MXene nanosheets only exhibit a single photothermal capability to promote osteogenesis with relatively low efficiency, so it is necessary to endow Ti_3_C_2_T_x_ MXenes with additional properties, such as proangiogenic activity and antioxidant and immunomodulatory functions. Furthermore, to achieve comprehensive therapeutic benefits at diabetic defect sites, PTT needs to be used in combination with other theranostic modes. The human body is rich in endogenous metal elements, such as copper (Cu), zinc (Zn), and magnesium (Mg), which are critically involved in cell metabolism and tissue regeneration. Among them, Cu, as an essential trace element for humans, not only exhibits excellent state-dependent (from Cu^2+^ to Cu^+^) enzyme-mimicking activity but also displays remarkable antibacterial and angiogenic activities 24. Furthermore, under on-demand NIR irradiation, Cu-based biomaterials exhibit enhanced antibacterial activity and improved tissue regeneration. Most recently, researchers found that combining Cu ions with other antibacterial therapies, such as PTT, could jointly enhance antibacterial and anti-biofilm effects against both *S. aureus* (Gram-positive bacteria) and *E. coli* (Gram-negative bacteria) while significantly reducing Cu ion usage and fostering vascularization and tissue healing 25. Considering the above rationale, GelMA and Alg-DA were employed as the fundamental backbone molecules of the hydrogel system, whereas MC nanosheets prepared by *in situ* decoration of copper ions (Cu^2+^) on the surface of MXene via physical absorption and electrostatic attraction acted as important multifunctional crosslinkers and bioactive ingredients to impart multiple functionalities to the GAD/MC hydrogel. The multiple crosslinked hydrogel networks formed by chemical bonds and physical bonds can simulate the cellular microenvironment and natural ECM and simultaneously impart the hydrogel with beneficial characteristics, including excellent injectability and moldability, strong bone tissue adhesion and self-healing properties, as well as improved mechanical and anti-swelling performance. In addition, MC, as an important component of the GAD/MC system, endows the hydrogel with outstanding photothermal effects and NIR/pH dual-responsive properties, which helps to realize the sustained and stimuli-responsive delivery of Cu^2+^ ions. In subcutaneous implantation experiments, the implanted GAD/MC hydrogel system significantly accelerated the ingrowth of vascular networks and the formation of dense cellular networks comprising pro-regenerative endogenous cells, including stem cells and anti-inflammatory M2 macrophages, across the entire interior of the scaffold at an early stage (14 days). In a well-established diabetic cranial defect model, the as-prepared GAD/MC hydrogel effectively reversed the inflammatory cascade, removed excessive ROS, and induced the polarization of macrophages from the proinflammatory (M1) phenotype to the anti-inflammatory (M2) phenotype, thus recreating an optimal regenerative microenvironment for vascularization and osteogenic differentiation while inhibiting osteoclast function and ultimately achieving complete bone regeneration. Notably, as an adjunctive approach for tissue regeneration, on-demand mild hyperthermia therapy induced by NIR stimuli could synergistically reinforce the biological activity and therapeutic efficacy of the hydrogel system, thus bolstering the healing process of diabetic bone defects. Through rational material design and functional optimization, the multifunctional injectable GAD/MC hydrogel demonstrated here possesses the capacity to fill irregular bone cavities in a minimally invasive manner and can be combined with mild PTT for diabetic bone defect treatment, highlighting its broad potential application in the field of orthopedics. Overall, this research provides new insight into the design of mild photothermal-assisted multifunctional injectable hydrogel systems for inflammatory-related bone healing, and the underlying biological mechanism underlying osteoimmune regulation is also elucidated.

## Results and Discussion

### Design of the multifunctional GAD/MC hydrogel system

Under pathological conditions (e.g., diabetes), desirable bone defect repair emphasizes the orchestration of immunomodulation and tissue regeneration, of which a conducive immune response is the precondition and stimulant for efficient osteogenesis and vascularization 10, 26. To circumvent all these aforementioned problems simultaneously and satisfy the anticipated demands for the treatment of diabetic bone defects, an injectable and biodegradable hydrogel therapeutic system (GAD/MC) with favorable immunomodulation and improved regenerative properties is designed and engineered. This high-performance hydrogel system, consisting of multiple covalent (free-radical photo-polymerization) and noncovalent (ionic crosslinking, metal coordination, hydrogen bonding, and π-π stacking) crosslinked networks, could meet the needs of different bone defects (various sizes and regions) in a minimally invasive injection manner, followed by rapid gel formation upon UV irradiation **(Scheme 1A)**. Remarkably, due to the optimized material selection and scaffold design, the resultant hybrid hydrogels with unique functional characteristics (injectability and moldability, *in situ* gelation, self-healing and adhesive abilities, stimuli-responsive release properties and photothermal effects) and biological activities (antioxidant and antibacterial capacities, immunomodulatory performance, and pro-osteogenic and proangiogenic potential) showed significant advantages over conventional hydrogels **(Scheme 1B)** 5, 7, 26. These advantageous properties endow the GAD/MC hydrogel system with remarkable therapeutic efficacy in the treatment of progressive inflammation-related issues under DM conditions. More significantly, the injectable GAD/MC hydrogel system in combination with NIR-assisted mild heat stimulation could serve as a controllable and smart thermal stimulator for synergistically promoting endogenous bone repair under comorbidity conditions (e.g., diabetes) by remodeling the dysregulated immune microenvironment and accelerating tissue regeneration **(Scheme 1C)**.

### Preparation, characterization, and optimization of MC nanosheets

**Figure 1:**
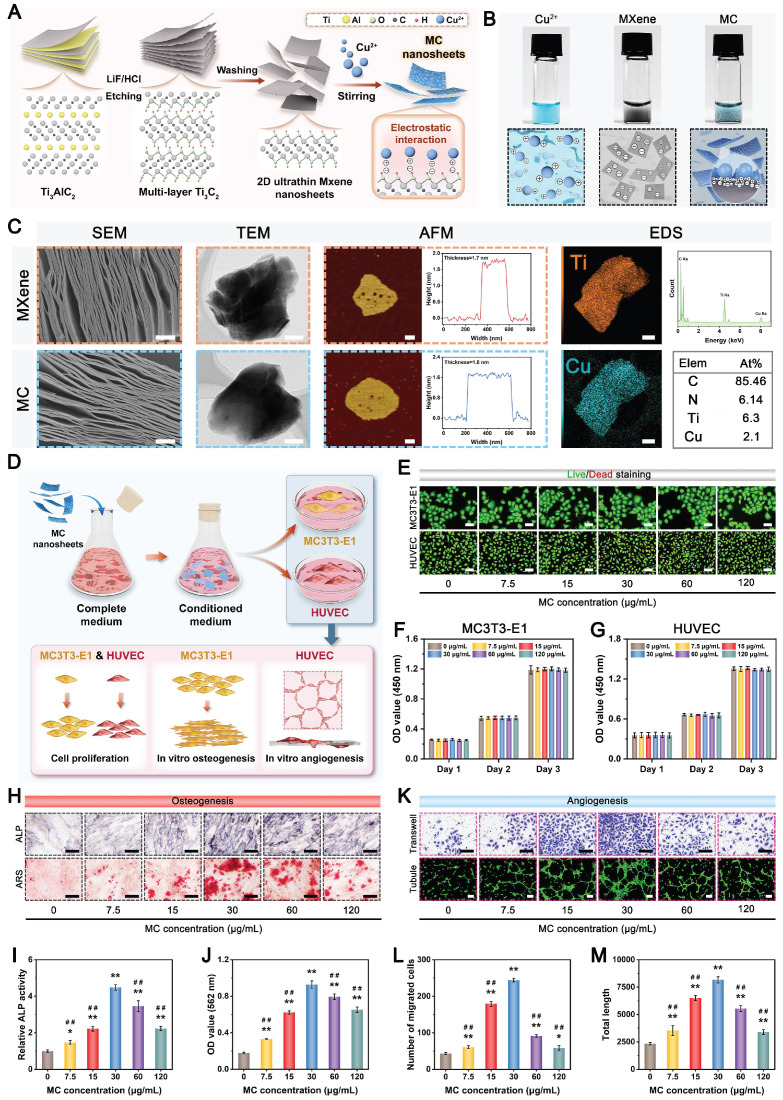
** Preparation, characterization, and bioactivity of MC nanosheets. (A)** Schematic illustration of the preparation of MXene and MC nanosheets. **(B)** Photographs of the Cu(NO_3_)_2_, MXene, and MC aqueous dispersions.** (C)** Structural and compositional characterization of MXene and MC nanosheets via SEM, TEM, AFM, and EDS elemental mapping analyses. Scale bar: 500 nm.** (D)** Schematic diagram of the experimental design for detecting the biological effects of MC nanosheets on MC3T3-E1 cells and HUVECs.** (E)** Effects of MC nanosheets at different concentrations on the proliferation of HUVECs and MC3T3-E1 cells, as determined by live/dead staining. Scale bar: 50 μm. **(F-G)** Effects of MC nanosheets at different concentrations on the proliferation of HUVECs and MC3T3-E1 cells, as determined by a CCK-8 assay. **(H)** Effects of MC nanosheets at different concentrations on the osteogenic potential of MC3T3-E1 cells, as determined by ALP and ARS staining assays. Scale bar: 100 μm. Quantification of **(I)** ALP and **(J)** ECM mineralization. **(K)** Effects of MC nanosheets at different concentrations on the angiogenic activity of HUVECs, as determined by Transwell migration and tube formation assays. Scale bar: 100 μm. Quantification of **(L)** cell migration and **(M)** angiogenesis. Data are presented as the mean ± SD (n = 3). *P < 0.05 and **P < 0.01 indicate significant differences compared with the control group. ^#^P < 0.05 and ^# #^P < 0.01 indicate significant differences compared with the 30 μg/mL-treated group.

Recently, increasing research interest has focused on the design and construction of 2D transition metal carbides (MXenes) and their derivatives because of their distinct advantages, including favorable biocompatibility, biodegradability, high photothermal conversion ability, and intrinsic antibacterial and osteogenic capacities 27, 28. In addition, the high specific surface area and unique multilayered structure of 2D MXene nanosheets make them promising nanoplatforms for the loading and delivery of other bioactive agents (e.g., Cu^2+^), endowing MXenes with more beneficial functions (e.g., proangiogenic activity). The synthesis procedures for the Cu-decorated MXene (MC) nanosheets are illustrated in **Figure 1A**. Pristine MXene nanosheets were first obtained through the selective etching of Ti_3_AlC_2_ powders in a mixed LiF/HCl solution. The obtained MXene nanosheets were immersed in a Cu(NO_3_)_2_ solution to capture Cu^2+^ via tight electrostatic attraction and then self-assembled into MC nanosheets. The optical images show the difference in color between the MXene and MC suspensions. The formation of a light blue suspension indicates that the MXene nanosheets effectively adsorbed metal ions (e.g., Cu^2+^) in the solution **(Figure 1B)**. As observed from the SEM images in **Figure 1C**, both the MXene and functionalized MXene (MC) nanosheets had multilayer accordion-like structures with similar lengths and sizes. After further sonication intercalation, high-resolution transmission electron microscopy (TEM) images and atomic force microscopy (AFM) images displayed a typical single-layer and 2D sheet-like morphology of these samples with a lateral size of dozens of nanometers. The height profile analysis indicated that the average thickness of the MC nanosheets was approximately 1.8 nm, which is similar to that of the MXene nanosheets. These results suggested that the decoration with Cu did not cause any morphological changes in the nanosheets. According to the energy dispersive spectrometry (EDS) elemental mapping analysis, the C, N, O, Ti, and Cu elements are evenly distributed on the as-synthesized MC nanosheets **(Figure 1C)**, further demonstrating the high-quality synthesis of the nanomaterial. Moreover, the MC nanosheets displayed the same X-ray diffraction (XRD) pattern as free Cu^2+^ **(Figure S1)**, suggesting that the Cu^2+^ within the nanosheets was not oxidized and existed in a free state in the MC nanosheets and could be released effectively. The decoration of Cu(II) was further verified by ζ potential analysis **(Figure S2A)**; it was found that the ζ potential of the raw MXene nanosheets was -23.6 mV, which led to a final charge of -16 mV for the synthesized MC nanosheets. Due to the presence of =O and -OH functional groups on the nanosheet surface 28, the raw MXene nanosheets exhibited a negative charge, which was conducive to their tight interaction with the positively charged Cu^2+^, further confirming that Cu(II) was successfully assembled on the surface of the MXene nanosheets by electrostatic forces. The composition and chemical state of the MC nanosheets were further investigated by X-ray photoelectron spectroscopy (XPS) analysis. Consistent with the results of the elemental mapping and EDS analysis, all the elements (C, N, O, Ti, and Cu) in the MC nanosheets could be detected and were indicated in the full-scan XPS survey spectrum **(Figure S2B)**. According to the high-resolution Cu 2p spectrum, the Cu 2p peak could be deconvoluted into two peaks: Cu 2p_3/2_ at 934.9 eV and Cu 2p_1/2_ at 955.2 eV, accompanied by characteristic shakeup satellite peaks at 942.6 and 962.9 eV, respectively **(Figure S2C)**, indicating the presence of Cu(II). These results demonstrated strong electronic interactions between the MXene nanosheets and Cu, which had a positive effect on the chemisorption property of Cu. Furthermore, thermogravimetric (TG) curves revealed that the MC nanosheets displayed enhanced thermal stability, indicating the coordination and electrostatic attraction of MXene and Cu^2+^ from another aspect **(Figure S2D)**. Altogether, the above analyses indicated that the MC nanosheets were synthesized successfully, while the phase composition and morphology of the nanosheets were unchanged after modification.

The as-prepared MC nanosheet plays a key role in the construction of the photoactivated GAD/MC hydrogel system, which not only acts as a skeleton material but also serves as a photothermal conversion agent. The ultraviolet-visible-near-infrared (UV-vis-NIR) absorption spectra showed that the MC nanosheets presented a prominent absorption band across the UV-vis region **(Figure S3A)**, indicating a high photothermal conversion potential, which was mainly derived from the exceptional NIR light absorption properties and high heat capacity of the MXene 21. Then, the photothermal performance of the aqueous dispersions of MC nanosheets was verified under 808 nm NIR light illumination (1 W/cm^2^, 5 min). Compared with that of the PBS solution, the temperature of the MC solution increased sharply and gradually reached a plateau of around 50 °C **(Figure S3B-C)**, indicating that MC nanosheets can quickly convert NIR light into heat energy. Furthermore, the MC nanosheets at a concentration of 100 μg/mL demonstrated a stable photothermal curve within five on/off cycles of NIR laser irradiation without any temperature loss **(Figure S3D)**, implying the high stability of MC nanosheets as a durable photothermal agent for PTT. In addition, the concentration of released Cu^2+^ ions from MC nanosheets with/without NIR irradiation was detected by inductively coupled plasma optical emission spectrometer (ICP-OES). As shown in **Figure S3E**, Cu^2+^ ions are released rapidly in the first 48 h, after which their release gradually slows. Moreover, the ion release behavior of the MC nanosheets in the presence of NIR light irradiation is further accelerated. The results demonstrated that the cumulative concentrations of Cu^2+^ ions released from the MC nanosheets after 120 h were 9.2 ppm and 15.8 ppm without and with NIR light irradiation, respectively, revealing that the photothermal properties of the MC nanosheets can accelerate ion release. The potential reason for this phenomenon may be that the amount of Cu dissociated from the MC nanosheets under NIR irradiation increased considerably owing to the elevated temperature, which stimulated the ionization of metal ions. Notably, SEM images further revealed that the multilayer structures of the MC nanosheets dissociated and fragmented following NIR irradiation **(Figure S4)**, which contributed to the decomposition of the nanosheets and accelerated the release of Cu^2+^, suggesting the feasibility of photothermal-responsive Cu^2+^ ion release. Thus, the above results indicate that we successfully prepared Cu-decorated MXene nanosheets with desirable photothermal properties and controllable ion release performance, which are feasible to provide a stimuli-responsive delivery nanosystem for bone regeneration applications.

Good biocompatibility is a basic requirement for biomedical engineering applications, prompting a series of investigations. Accordingly, the concentration-dependent cytotoxicity of the prepared MC nanosheets and their biological activity were then assessed in subsequent experiments. MC3T3-E1 preosteoblastic cells and human umbilical vein endothelial cells (HUVECs) were cocultured with MC nanosheets at different concentrations (0, 7.5, 15, 30, 60, and 120 μg/mL) **(Figure 1D)**. During the experimental period, the cell proliferation capacity was quantitatively and qualitatively evaluated by live/dead cell staining and the cell counting kit-8 (CCK-8) assay **(Figure 1E-G)**. The results demonstrated the superior biocompatibility of MC, which had no apparent adverse effects on the survival or proliferation of either cell line. After incubation for 3 days, live/dead staining images revealed a high percentage of living cells (green fluorescence) and few dead cells (red fluorescence) in both cell lines **(Figure 1E)**, indicating favorable proliferative activity at concentrations ranging from 7.5 to 120 μg/mL. Moreover, the results of the CCK-8 assay demonstrated that the proliferation rates of MC3T3-E1 cells and HUVECs in all the MC-treated groups closely mirrored those in the control group after 1, 2, and 3 days of incubation **(Figure 1F-G)**, implying the harmonious coexistence between the nanosheets and the cultured cells. The cytotoxicity of metal nanomaterials has been considered a major obstacle to their clinical application 29. The main reason for our findings might be that the inclusion of Ti_3_C_2_T_x_ MXene nanosheets was able to efficiently adsorb free Cu^2+^ ions by electrostatic and coordination interactions, which largely prevented the explosive release of Cu^2+^ ions, thus effectively addressing the safety implications associated with Cu^2+^ toxicity. Although there was no detectable cytotoxicity, long-term biosafety of the MC nanosheets was necessary for broader application potential.

Intriguing discoveries from previous studies have shown that MXene nanosheets can modulate osteoblast differentiation, while bioactive Cu^2+^ ions exhibit potent concentration-dependent proangiogenic activity 27. After confirming the good biocompatibility of the MC nanosheets, we investigated the effects of different concentrations (0-120 μg/mL) of MC on the biological functions of osteoblasts and endothelial cells. The ability of the MC nanosheets to stimulate the osteogenesis and mineralization of MC3T3-E1 cells was analyzed using alkaline phosphatase (ALP) activity and alizarin red S (ARS) staining assays **(Figure 1H)**. It was noticeable that MC treatment (30 and 60 μg/mL) elicited the highest ALP expression and more mineralized nodules than any other groups **(Figure 1I-J)**, suggesting excellent osteoinductive activity. The same phenomenon was also found in the Transwell migration and tube formation assays **(Figure 1K)**, revealing that MC treatment (15 and 30 μg/mL) effectively promoted the migration and angiogenesis of HUVECs **(Figure 1L-M)**. In addition to shielding the toxicity of the Cu^2+^ ions, the functionalized MC nanosheets further enhanced the osteogenic and angiogenic activities due to the intrinsic osteoinductive properties of the MXene nanosheets and the proangiogenic activity of the Cu^2+^ ions. According to preliminary screening results, MC nanosheets at a final concentration of 30 μg/mL achieved a good balance between overall biological activity and cytocompatibility, demonstrating superior performance in inducing osteogenesis and vascularization; thus, MC nanosheets were selected for optimal encapsulation in the hydrogel for follow-up experiments. Collectively, we successfully prepared MC nanosheets with *in situ* decoration of Cu(II) while preserving the functionality and structure of the MXene.

### Preparation and characterization of GAD/MC hydrogels

**Figure 2:**
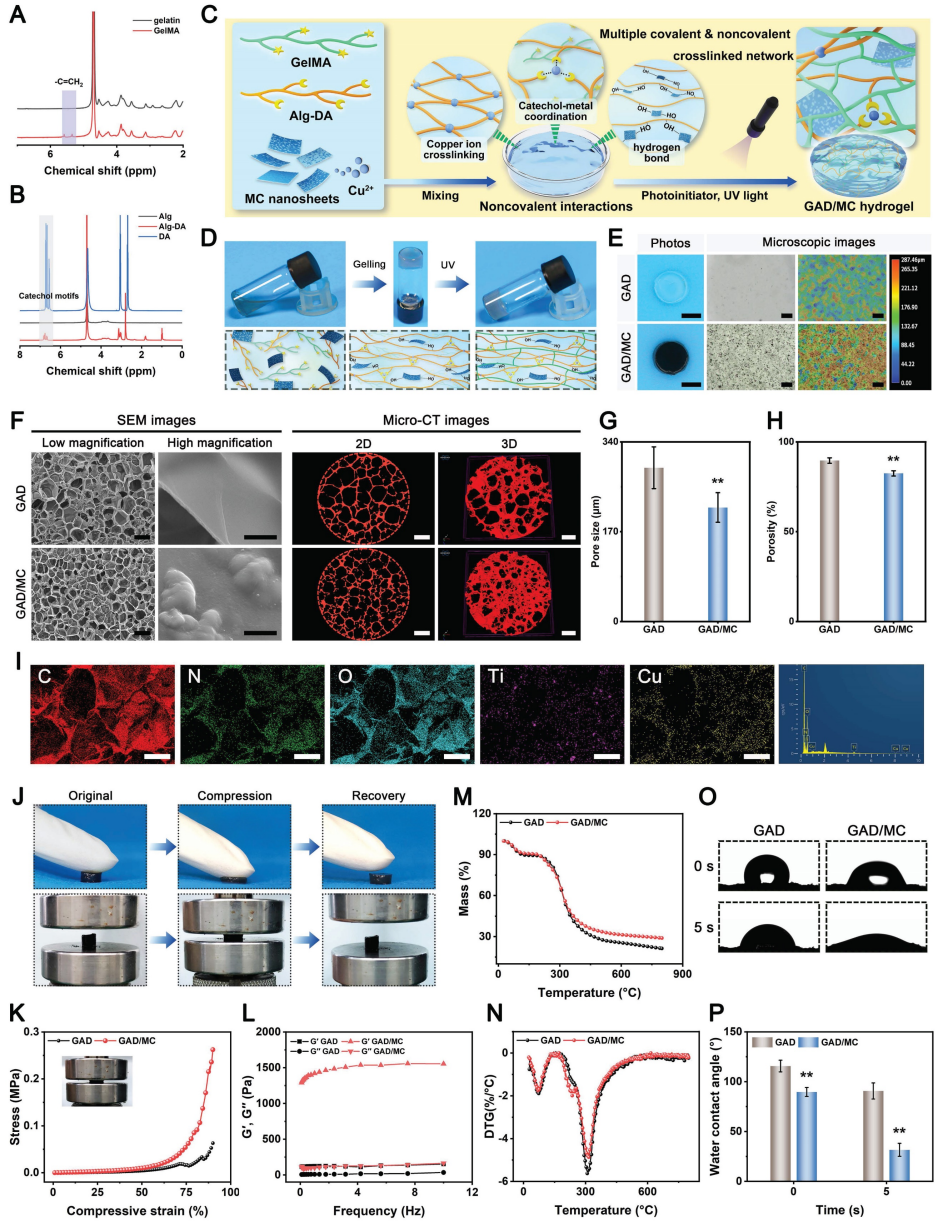
** Preparation and characterization of the GAD/MC hydrogel. (A)** ^1^H NMR spectra of gelatin and GelMA. **(B)** UV-vis absorption spectra of Alg, DA, and Alg-DA. **(C)** Schematic illustration of the fabrication process of the GAD/MC hydrogel. **(D)** Photographs of the solution before and after hydrogel transition.** (E)** Photographs (left), optical images (middle), and corresponding 3D surface maps (right) of different hydrogels. Scale bar: 5 mm (digital images) and 200 μm (optical images). **(F)** SEM and micro-CT images of different hydrogels. Scale bar: 300 μm (low-magnification SEM images), 10 μm (high-magnification SEM images), and 200 μm (micro-CT images).** (G-H)** Pore size and porosity of different hydrogels. **(I)** EDS elemental mapping images of the GAD/MC hydrogel. Scale bar: 100 μm.** (J)** Photographs of the GAD/MC hydrogel for standing compression. **(K)** Compressive stress-strain curves of different hydrogels. **(L)** Rheological behaviors of different hydrogels. **(M-N)** TG analysis and DSC images of different hydrogels. **(O-P)** Hydrophilicity and corresponding water contact angle measurements of different hydrogels. Data are presented as the mean ± SD (n = 3). *P < 0.05 and **P < 0.01 indicate significant differences compared with the GAD group.

In the context of bone regeneration, the selection of appropriate matrix materials and scaffold designs is essential for achieving satisfactory healing outcomes. Compared with existing synthetic and semisynthetic polymer scaffolds, natural biopolymer-based hydrogels provide obvious advantages for the efficient encapsulation and delivery of bioactive agents in terms of biocompatibility, biodegradability, low immunogenicity, promotion of cell adhesion and growth, and so forth 30. Thus, in the present work, both GelMA and Alg-DA were selected as the main network molecules, followed by the incorporation of MC nanosheets, which is likely a win-win strategy. Besides, GelMA-based hydrogels have been widely investigated for diverse biomedical applications, especially in the field of bone regeneration, due to the advantages of *in situ* curing, easy control, and use 31. To enable *in situ* crosslinking under UV light, the methacrylate group was grafted onto the polymer chains through esterification, as shown in **Scheme 1A**. The synthesis of GelMA was confirmed by proton-1 nuclear magnetic resonance (^1^H NMR) analysis **(Figure 2A)**. New signals corresponding to methacrylate-based double bonds were observed at approximately 5.3 and 5.6 ppm, indicating the presence of -C=CH_2_, which confirmed the successful synthesis of GelMA. The substitution degree of the methacrylate group on gelatin was calculated to be 45.45%. On the other hand, the natural compound sodium alginate (Alg) possesses excellent biocompatibility and abundant functional groups that can form ionic crosslinks through divalent cations of metal ions (e.g., Cu^2+^). As a catecholamine derived from marine mussels, dopamine (DA) is well known for its tissue adhesive capability. Leveraging this catechol chemistry, the tissue adhesion of Alg was further improved by the grafting of DA. Furthermore, DA can also provide antioxidant, anti-inflammatory, metal coordination, and osteogenic properties to hydrogels 32. Thus, this DA-grafted-Alg (Alg-DA) with multiple functions was chosen as another matrix composition to construct the hydrogel network in this study. As shown in **Scheme 1A**, catechol group-containing DA was grafted into the backbone of Alg using classical EDC/NHS coupling chemistry, which is favorable for the subsequent formation of coordination bonds with metal ions (e.g., Cu^2+^). The successful grafting of catechol moieties was verified by ^1^H NMR spectroscopy and UV-vis absorption spectroscopy. The presence of catechol proton-specific peaks at approximately 6.7 ppm was clearly observed **(Figure 2B)**, thus validating successful conjugation. The substitution degree of the DA group on Alg-DA was calculated to be 57.52%. Finally, the UV-vis profile of Alg-DA exhibited a strong ultraviolet absorption peak at 280 nm **(Figure S5)**, which is the characteristic peak of the catechol moiety 18. Taken together, the aforementioned results suggested that both GelMA and Alg-DA were successfully obtained.

Based on previous research findings 33, GAD hydrogel containing 7% GelMA and 3% Alg-DA was selected as a representative hydrogel for loading MC nanosheets, which was named the GAD/MC hydrogel. **Figure 2C** shows a schematic diagram of the preparation and multiple crosslinking mechanisms of the hydrogel, in which lithium phenyl-2,4,6-trimethylbenzoylphosphinate (LAP) serves as the photoinitiator. The MC nanosheets were mixed with the GAD precursors before photo-crosslinking to construct the first dynamic metal coordination bonds and ionic crosslinked network at room temperature. When the MC nanosheets are dispersed *in situ* into a precursor solution containing GelMA and Alg-DA, some of the MC nanosheets slowly release Cu^2+^ ions, forming coordination bonds with the carboxyl and amino groups of Alg-DA chains and triggering ionic crosslinking; simultaneously, the residual intact MC nanosheets are incorporated and embedded in the network, functioning as nanofillers that form supramolecular interactions with Alg-DA. The GAD polymer interacted with the MC nanosheets primarily due to the strong binding affinities of the catechol groups (present in Alg-DA) to divalent Cu ions through coordination or hydrogen bonding. Next, to further strengthen the network of the hydrogel, photo-triggered covalent bonds were introduced into the original hydrogel network. After exposure to UV light, the C=C bonds in the methacrylate group (present in GelMA) can undergo free radical polymerization to form C-C bonds, which provide a rigid skeleton in the hydrogel network. These multiple crosslinked networks with covalent and noncovalent bonds endow the hydrogel with good mechanical and self-healing properties, excellent injectability and moldability, and strong bone tissue adhesion and anti-swelling capabilities. The bulk hydrogel formed by the photo-crosslinking of GelMA and Alg-DA (denoted as GAD) served as a control. The gelation of the GAD/MC injectable hydrogel is schematically depicted in **Figure 2D**. By tilting the bottle to change the liquid level, we observed that the hydrogel transformed from a flowing liquid into a crosslinked solid, verifying the successful construction of the GAD and GAD/MC hydrogels. Macroscopic photographs indicated that the obtained GAD hydrogel exhibited a colorless translucent appearance, the color of which changed to dark black after the addition of the MC nanosheets **(Figure 2E)**, providing further evidence of the successful encapsulation of the MC nanosheets. Remarkably, even in the absence of UV light irradiation, the GAD/MC pre-gel mixture could form a free-standing hydrogel, which was primarily because the numerous functional groups, including catechol, carboxy, and hydroxy groups, on Alg-DA reacted with the Cu^2+^ ions released from MC to form ionic crosslinks, metal coordination bonds, and hydrogen bonds *in situ*. These results confirmed the successful synthesis of GelMA, Alg-DA, and MC nanosheets, as well as the successful preparation of GAD/MC hydrogels based on ionic crosslinking and coordination reactions as well as subsequent photo-crosslinking. Collectively, the proposed multiple crosslinking strategy in this work has the advantages of a fast reaction speed, simple operation process, and mild reaction conditions at room temperature, showing great promise for practical applications.

In the following characterization experiments, the surface morphology, microstructures, mechanical properties, and hydrophilicity of the hydrogels were investigated. The microscopic images and 3D surface profiles confirmed the good dispersion of the as-synthesized MC nanosheets, which were uniformly embedded within the hydrogel matrix without obvious agglomeration and potentially participated in the construction of the hydrogel network as a specific crosslinker. Simultaneously, the results also showed that MC nanosheets loaded with Cu^2+^ ions and rich functional groups (-OH, =O, -F, etc.) are more likely to be incorporated into the polymer network of hydrogels through multiple covalent or noncovalent interactions. After lyophilization, the cross-sectional SEM images further showed that the MC nanosheets were homogeneously distributed within the hydrogel matrix **(Figure 2F)**, as evidenced by the presence of some wrinkles and ripples on the pore walls of the GAD/MC hydrogels, which exhibited a rough surface that facilitated cell adhesion and proliferation 17.

In contrast, the GAD hydrogel had a relatively smooth surface. A porous structure is a crucial feature of ideal bone repair scaffolds. It was also demonstrated that two kinds of hydrogels possessed uniform and interconnected 3D porous structures with pore sizes ranging from 150-300 µm, which aligned with clinical requirements. Moreover, the desirable pore uniformity and connectivity demonstrated substantial capacities in guiding cell infiltration, penetration, and growth as well as facilitating the exchange of nutrients and metabolites and the release of bioactive substances 34. Interestingly, compared with GAD hydrogels, the introduction of MC nanosheets significantly affected the pore structure of the hydrogels. **Figure 2G-H** revealed that the pore size and porosity of the hydrogel decreased with the addition of MC nanosheets, which may be closely associated with the increased crosslinking density in the polymer network. To provide more detailed information from 3D observations, micro-CT was utilized to examine the architecture formed in the hydrogels. Both 2D- and 3D-reconstructed micro-CT images revealed that the *in situ* incorporation of MC nanosheets resulted in a highly dense and tightly packed microstructure of the hydrogel **(Figure 2F** and **Movies S1 and S2)**, closely recapitulating the architecture of natural cancellous bone 35. These 3D bulk network microstructures formed in the GAD/MC hydrogel provide a favorable platform for cell and tissue ingrowth after implantation 36. These results were consistent with those of previous studies, revealing that the crosslinking density plays a major role in determining the pore size of hydrogels 37. The addition of MC can increase the number of Cu^2+^ ions crosslinked by the catechol, carboxy, and hydroxy groups of Alg-DA, yielding higher crosslinking density and smaller pores in the final synthesized hydrogel. The EDS elemental mapping images further demonstrated the existence of homogeneously distributed C, N, O, Ti, and Cu elements in the GAD/MC hydrogel matrix **(Figure 2I)**, which suggested that the MC nanosheets participated in the construction of the hydrogel network as functionalized crosslinkers. Additionally, the characteristic elemental peaks of Ti and Cu in the EDS spectra demonstrated their efficient incorporation into the hydrogel networks, further illustrating the successful preparation of the GAD/MC hybrid hydrogel.

The mechanical performance of the prepared hydrogel was crucial because it determined its long-term stability and structural support during bone repair 38. **Figure 2J** shows the mechanical properties of the scaffolds through the optimized preparation procedure. As the applied loads were released, the GAD/MC composite hydrogels could completely recover their original shape without obvious breakage or collapse, demonstrating excellent elasticity and mechanical performance. We further investigated the mechanical properties of the hydrogels using a universal testing machine. **Figure 2K** illustrates the compression stress-strain curves of the hydrogels. As expected, the compressive mechanical properties of the hydrogels were enhanced with the addition of MC nanosheets. As shown in **Figure S6**, the compressive strength and modulus of the GAD/MC hydrogel were superior to those of the GAD hydrogel, highlighting the beneficial impacts of MC nanosheet incorporation on its mechanical properties. The underlying mechanism was likely because the MC nanosheets acted as reinforcing nanofillers to effectively block crack propagation generated in the hydrogel matrix under external force. In addition, MC nanosheets can form various covalent and noncovalent bonds with hydrogel polymers, such as metal coordination bonds, hydrogen bonding, and electrostatic interactions, thus increasing the crosslinking density and mechanical performance of the hydrogel. Importantly, after the incorporation of MC nanosheets, the elastic modulus of the GAD/MC hydrogel was approximately 2.5 MPa **(Figure S6)**, which has been demonstrated to be suitable for the osteogenic differentiation of osteogenesis-related cells 39. Moreover, the GAD/MC hydrogel maintained structural integrity during the experiment **(inset of Figure 2K)**, suggesting that it can withstand mechanical stresses when implanted in bone defects. Similarly, as shown in the rheological curves, the storage modulus (G′) of the hydrogels remained constant and consistently higher than their loss modulus (G′′), which did not display any obvious structural failure, indicating stable gelation and favorable mechanical properties **(Figure 2L)**. The addition of MC nanosheets effectively increased the storage modulus of the hydrogels, resulting in enhanced mechanical performance, which was in accordance with the results of the compressive test. Importantly, the coordination of Cu and Alg-DA could serve as a bonding motif between GelMA molecules to strengthen the hydrogel network, preventing the introduction of a carbon-carbon double bond that most adhesive hydrogels possess to improve the mechanical properties of the resulting hydrogel. These features suggested that the GAD/MC hydrogel was more advantageous for maintaining structural stability and providing basic mechanical support for long-term bone regeneration. Thermogravimetric and differential thermal analysis (TG/DTA) also revealed that the GAD/MC hydrogel possessed a higher denaturation temperature than the GAD hydrogel **(Figure 2M-N)**, which was attributed to the formation of highly dense multiple crosslinked networks that efficiently enhanced the thermostability of the GAD/MC hydrogel. To meet the demands for bone regeneration, the favorable mechanical properties of hydrogels need to be considered. In the hydrogel system, the incorporation of an appropriate amount of MC nanosheets improved the mechanical performance and stability of the hydrogel because of the formation of additional physical crosslinking and coordination bonds between the Cu^2+^ released from the nanosheets and the catechol of Alg-DA. This also confirmed the potential of MC as a reinforcement nanobuilding block and crosslinker in the hydrogel network. In addition, GelMA, together with MC and Alg-DA, could also form hydrogen bonds via intra- and intermolecular hydrogen bonds. Thus, these results collectively demonstrated that the addition of MC nanosheets improved the mechanical properties of the hydrogel, allowing it to withstand compressive loading for continuous operation and avoiding irreversible structural damage in local defect areas. Except for the proper mechanical properties, the surface hydrophilicity of biomaterial scaffolds also plays an imperative role in the early adhesion and differentiation of osteoblasts 40. As shown in **Figure 2O-P**, the addition of MC nanosheets substantially enhanced the hydrophilicity of the hydrogel, which was potentially ascribed to the special formation process of the multiple crosslinked hydrogel network structure. The improved mechanical strength and hydrophilicity facilitated physical interactions between the hydrogel and host cells, which is thought to be beneficial for regulating subsequent cell function and behavior (e.g., cell recruitment, adhesion, proliferation, and differentiation) and ultimately promoting tissue regeneration and integration 41.

**Figure 3:**
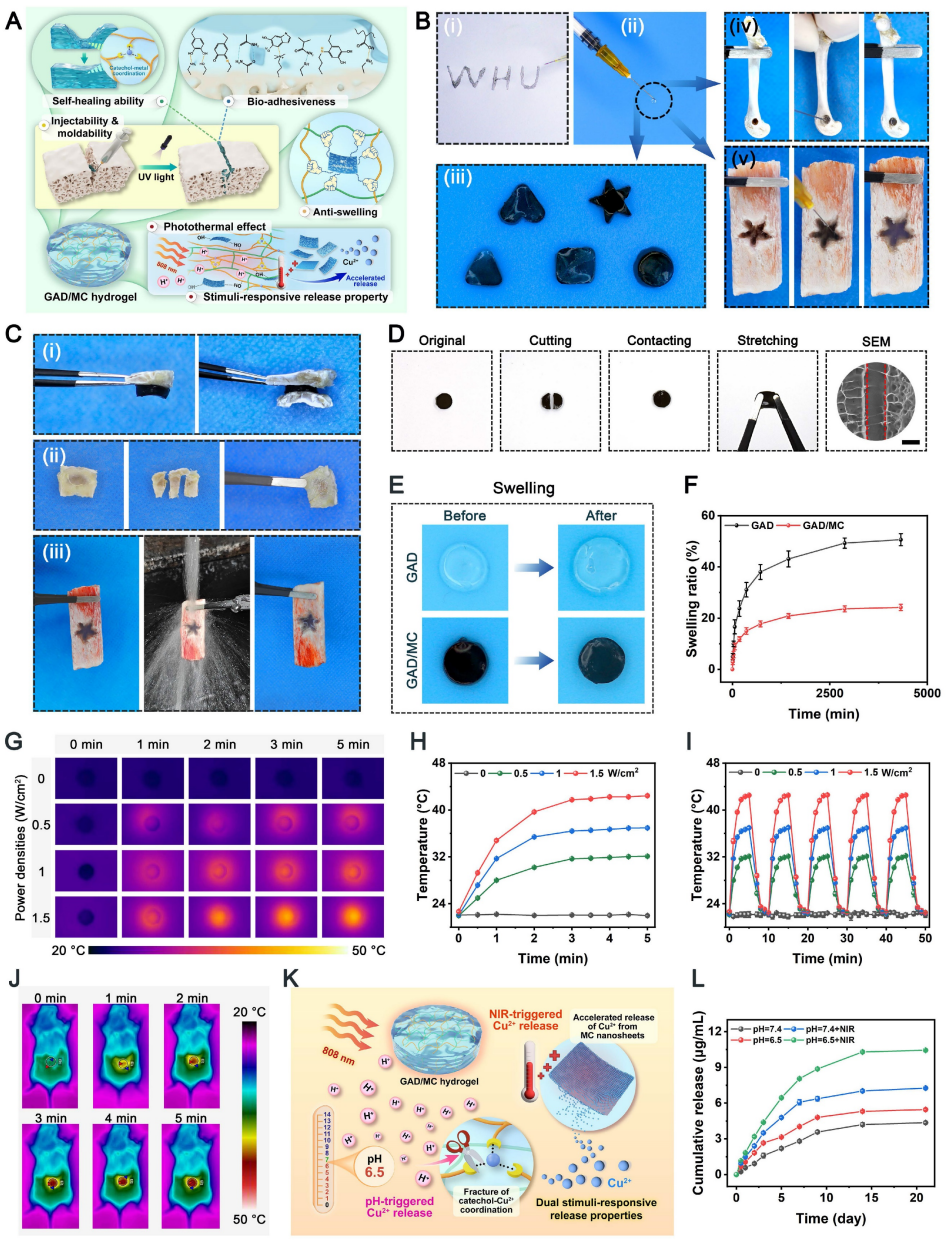
**Multifunctional characteristics of the GAD/MC hydrogel. (A)** Schematic illustration of the potential application of the GAD/MC hydrogel. **(B)** Photographs of the injectability and moldability of the GAD/MC hydrogel. **(C)** Photographs of the bone tissue adhesion of the GAD/MC hydrogel. **(D)** Photographs of the macroscopic self-healing process of the GAD/MC hydrogel and SEM images of the aggregated hydrogel. **(E)** Photographs of morphological changes in the hydrogels before and after immersion in PBS. **(F)** Swelling ratio of the hydrogels. **(G)** Real-time infrared thermal images of the GAD/MC hydrogel under 808 nm laser irradiation at various power densities (0, 0.5, 1, and 1.5 W/cm^2^) for 5 min. **(H)** Temperature curves of the GAD/MC hydrogel under 5 min of NIR irradiation. **(I)** Photothermal stability of the GAD/MC hydrogel after five laser on/off cycles. **(J)** Real-time infrared thermal images of the GAD/MC hydrogel under NIR light (808 nm, 1.5 W/cm^2^) for 5 min after *in vivo* subcutaneous implantation. **(K)** Schematic illustration of the NIR/pH dual-responsive release properties of the GAD/MC hydrogel. **(L)** Cumulative release curve of Cu^2+^ from the GAD/MC hydrogel under different conditions. Data are presented as the mean ± SD (n = 3).

The successful fabrication of the GAD/MC hydrogel system encouraged us to investigate its multiple functionalities **(Figure 3A)**. Large bone defects are typically characterized by an irregular shape, deep location, and numerous discontinuous bone fragments. Injectable hydrogels with excellent moldability, strong bone tissue adhesion and self-healing properties, and the capability to match irregular defects have emerged as attractive alternatives for the treatment of large bone defects in the clinic 19. **Figure 3B** shows that the GAD/MC hydrogel could be continuously injected into the culture dish through a 26-gauge needle without clogging, forming a “WHU” font that maintained the hydrogel state even after the shear force was removed. Moreover, the ability of the GAD/MC hydrogel to be prepared into various complex shapes is shown in **Figure 3B**, suggesting its excellent moldability, which enabled adequate filling of bone defects with irregular sizes and shapes. This could be attributed to the rapid photo-polymerization of the hydrogel precursor under UV light after injection, followed by gelation to accommodate various shapes of molds. This excellent injectability and shape adaptability align with therapeutic requirements, allowing the hydrogel to be shaped into different forms as needed to accommodate various bone defect sizes 4. Subsequently, we directly injected the hydrogel precursor into defects in fresh rat femoral condylar defects and porcine rib star-shaped defects to observe whether the GAD/MC hydrogel could maintain its injectability and gelation properties to accommodate irregularly shaped and deep defects in biological tissues **(Figure 3B)**. Benefiting from its remarkable injectability and moldability, the GAD/MC hydrogel system could completely fill these cavities due to the irregular shapes of the bone defects and achieve rapid gelation, thus strengthening the tightness of the interface between the materials and the surrounding tissue, which provided distinctive advantages for localized minimally invasive applications. Furthermore, the GAD/MC hydrogel exhibited good bone tissue adhesion **(Figure 3C)**, which not only ensured stable adhesion around the bone defect but could also serve as a bio-adhesive to fix comminuted bone fragments made on fresh rat calvaria to simulate bone fracture under physiological conditions. To further assess the adhesive properties of the GAD/MC hydrogel, a lap shear test was performed, as schematically shown in **Figure S7A**. The adhesion strength of the hydrogel increased from 13.6 to 27.7 kPa (**Figure S7B**), indicating that the addition of MC nanosheets significantly enhanced the adhesion of the hydrogel to biological tissues. These promising results were consistent with previous reports on mussel-inspired materials containing catechol groups and catechol-metal ion coordination bonds with strong adhesion effects on bone tissue 42. In addition, good adhesion of biomaterials to bone defect sites is essential for safe and efficient bone regeneration. As shown in **Figure 3C**, the solidified GAD/MC hydrogel firmly bonded to the star-shaped defects in the wet porcine rib without falling under a forceful flush of water (**Movie S3**), indicating its potential to serve as a bio-adhesive *in vivo* and in a dynamic environment. The outstanding bio-adhesive properties and tissue integration protect the hydrogels from detaching from the defect site, leading to sufficient matching for irregular defects. By coordinating Alg-DA with Cu^2+^ ions, the adhesive and self-healing properties of the resulting hydrogels were enhanced. More importantly, the oxidation of catechol groups in aqueous solutions was dramatically suppressed after metal-phenol coordination. These unique characteristics allowed our GAD/MC hydrogel to stably adhere to the surrounding native bone after filling bone defects and fulfill its biological activity for accelerated *in situ* bone regeneration in the wet and dynamic environment of the bone cavity. Altogether, the excellent bio-adhesive ability of the GAD/MC hydrogel was likely correlated with the following potential mechanism: 1) The abundant free catechol groups in the hydrogel tended to form reactive catechol-quinone groups due to partial deprotonation under physiological conditions, which enabled Schiff-base reactions or Michael-type reactions between the hydrogel and the nucleophiles (amines, thiol, and amide bonds) on the surface of the biological tissue 43. 2) The introduction of the MC-enhanced network, coordination and electrostatic interactions between the Cu^2+^ and carboxyl groups on Alg-DA, and various physical interactions, such as hydrogen bonds, coordination bonds, electrostatic interactions, π-π stacking, and π-cation interactions, further improved the cohesion and wet tissues of the network. 3) The free catechol groups in the hydrogel network tended to undergo intra- and intermolecular crosslinking, which led to further solidification of the adhered hydrogel 44. The consolidation allows irreversible anchorage of the hydrogel to the tissue surface, which improves bio-adhesion. Moreover, we also assessed the self-healing behavior of the GAD/MC hydrogel by macroscopic observation and SEM **(Figure 3D)**. The cylindrical hydrogel samples were cut into two pieces, which were then put together without external intervention. It was observed that two hydrogel pieces could recombine and remain intact without breakage even when stretched by tweezers. The self-healing behavior of the GAD/MC hydrogel was assessed by rheological recovery tests. As shown in **Figure S8**, when a high dynamic strain (500%) was applied, the energy storage modulus G′ decreased significantly, and the loss modulus G″ increased significantly, indicating the disruption of the hydrogel structure. However, when a low strain (1%) was applied, the G′ values were able to recover to their initial values, implying a rapid “sol-to-gel” transition. Even after five cycles, the hydrogel system was able to recover and form a stable hydrogel structure, demonstrating the rapid and efficient self-healing ability. The exceptional self-healing capacity of the GAD/MC hydrogel primarily stems from the formation of dynamic and reversible metal coordination bonds and physical bonds (e.g., electrostatic forces, hydrogen bonds, and π-π stacking interactions) 45. Notably, the cut line was still visible in the SEM images, probably as a result of the unfit joined interface. The strong bone tissue adhesion and self-healing properties of the prepared GAD/MC hydrogel were conducive to maintaining its integrity spontaneously, even after the implanted material was compromised by external forces during dynamic movement. Overall, these results highlighted the remarkable benefits of injectable GAD/MC hydrogels for bone regeneration applications, particularly for ease of surgical manipulation and minimal invasiveness, which make them suitable for repairing irregular bone defects and multiple fragments in complex bone fractures, especially bone defects in the oral and maxillofacial regions, as well as avoiding contact with external pathogens during surgery.

The *in vitro* swelling properties and biodegradation behavior of the hydrogels were further investigated, as they are important parameters that influence the long-term therapeutic efficacy of bone repair and reconstruction. Not surprisingly, the introduction of MC nanosheets could also affect the swelling ratio and enzymatic degradation of the hydrogels. All the hydrogels reached the swelling equilibrium state after about 24 h and maintained a stable swelling rate for the following experimental period. As shown in **Figure 3F**, after 72 h of incubation, the equilibrium swelling ratio of the GAD hydrogel was significantly higher than that of the GAD/MC hydrogel, suggesting that the addition of MC nanosheets endowed the GAD/MC hydrogel with excellent anti-swelling properties. The prepared MC nanosheets act as crosslinking agents, offering abundant crosslinking sites and rigid properties that effectively limit the diffusion of physiological fluid into the hydrogel network. Meanwhile, due to the formation of metal coordination bonds and physical crosslinking, the resultant GAD/MC hydrogel demonstrated a much denser network with a smaller swelling ratio, which was in accordance with the results of the SEM and micro-CT images. Likewise, this further indicated that the prepared MC nanosheets, which serve as reinforcing nanofillers, together with additional physical crosslinking and coordination interactions through Cu^2+^ ions, have a positive influence on maintaining the mechanical support of the hydrogel and restricting its swelling expansion, thus providing stable and sufficient healing space for cellular infiltration and tissue regeneration. The macroscopic appearance of the hydrogel samples before and after swelling further validated the restriction of swelling behavior resulting from the multiple crosslinked network structure **(Figure 3E)**, indicating that our GAD/MC hydrogel could adapt to the complexity of the local defect rather than ectopic extrusion. These unique characteristics were capable of protecting the hydrogels from detaching from the defect site, which allowed our GAD/MC hydrogel to stably adhere to the surrounding native bone and fulfill its biological activity for accelerated *in situ* bone regeneration in the wet and dynamic environment of the bone cavity. The biological function of the hydrogel is also related to its degradation behavior; thus, the *in vitro* enzymatic degradation of the hydrogels was investigated here. As shown in **Figure S9**, the GAD/MC hydrogel was more stable than the pure GAD hydrogels in a physiological environment on account of multiple chemical and physical crosslinking reactions. It is well-established that the degradation properties of hydrogels are closely associated with their network structure and crosslinking density 46. The multiple crosslinked network structures of the GAD/MC hydrogel containing covalent and noncovalent interactions are relatively stable under physiological conditions and provide adequate structural support and mechanical stability for long-term bone defect repair and healing. During the early stage of tissue regeneration, gradual degradation of the hydrogel is crucial for tissue ingrowth and blood vessel formation because it provides space for cell infiltration and facilitates tissue remodeling. Therefore, the as-prepared GAD/MC hydrogels can be gradually degraded and absorbed when applied *in vivo* without removal, avoiding secondary surgery, which makes them highly advantageous and attractive for bone regeneration research.

In the past few decades, mild PTT, an emerging non-invasive treatment modality, has attracted tremendous attention from researchers due to its wide application prospects in the field of bone tissue engineering 47. Evidence from both laboratory and clinical settings has substantiated that NIR-assisted on-demand mild thermal stimulation (~45 °C) can effectively promote cell biomineralization and neovascularization, thereby boosting tissue regeneration 48. Consequently, aside from the desirable structural and physical features, mild photothermal effects should be taken into consideration during the architecture of functional hydrogels for bone repair. However, to avoid excessive thermal damage to normal cells and surrounding tissues, the optimized parameters, including the power density of the laser irradiation and irradiation time, should be systematically screened. Therefore, the photothermal conversion performance of the hydrogels was evaluated under 808 nm NIR light irradiation at various power densities (0, 0.5, 1, and 1.5 W/cm^2^). As illustrated in **Figure 3G-H**, the temperature changes became increasingly prominent with increasing laser irradiation power density and irradiation time. For instance, when subjected to NIR irradiation at a power density of 1.5 W/cm^2^, the local temperature in the GAD/MC hydrogel increased from 22.6 ± 0.2 °C to 41.7 ± 0.1 °C after 3 min of NIR irradiation, and its core temperature stabilized at 41-43 °C within 3-5 min, which was sufficient to provide mild heat stimulation for tissue regeneration. Compared with the literature on photothermal biomaterials, the temperate rise was not significant in our designed hydrogel, which was targeted to be used for tissue regeneration rather than for antibacterial or antitumor therapy 49, 50. Stable photothermal properties are the basic requirement for mild PTT. Accordingly, to evaluate the photothermal stability of the GAD/MC hydrogel, periodic NIR irradiation was applied. As depicted in **Figure 3I**, no apparent temperature decrease was detected in the GAD/MC hydrogel even after five consecutive heating-cooling cycles, highlighting that GAD/MC has excellent photothermal stability, which is suitable for reusability *in vivo*. Then, the *in vivo* photothermal properties of the GAD/MC hydrogel were examined using a BALB/c mouse subcutaneous implantation model. The prepared GAD/MC hydrogel samples were subcutaneously implanted into the dorsum of mice and irradiated with an 808 nm NIR laser (1.5 W/cm^2^, 5 min). The changes in temperature and infrared thermal images of the implanted site were recorded by using an infrared thermal imaging instrument **(Figure 3J)**. Excitingly, the GAD/MC hydrogel still showed an exceptional NIR-mediated temperature increase and eventually reached an equilibrium temperature of ~42 °C, which remained within the temperature range required for mild hyperthermal therapy. There is accumulating evidence that mild PTT at temperatures less than 45 °C is more suitable for clinical application, while excessive temperature (>45 °C) adversely affects cell viability and metabolism, such as decreased cell viability, apoptosis, and overexpression of inflammation 14. Thus, both *in vitro* and *in vivo* experiments showed that the GAD/MC hydrogel can produce mild heat after 5 min of NIR laser irradiation (808 nm, 1.5 W/cm^2^), which paved the way for further experiments. Based on this, a power density of 1.5 W/cm^2^ and NIR irradiation for 5 min were used for subsequent *in vitro* and *in vivo* studies.

Leveraging the advantages of mild hyperthermia and injectable hydrogels, designing a smart hydrogel system capable of responding to internal and external stimuli (such as pH, ROS, heat, etc.) in a precise and controlled manner to achieve on-demand stimuli-responsive bioactive substance release holds great potential for future clinical applications in diabetic bone regeneration 51. Because of the enrichment of acid metabolites, the injury sites of refractory bone defects (e.g., diabetic bone defects, infected bone defects or infected diabetic bone defects) are usually featured with a low pH (around 5.5-6.5). Furthermore, higher levels of inflammation and apoptosis in the initial stage of bone injury are also closely related to a more acidic pH in the microenvironment 52. As an indispensable trace element in the human body, bioactive Cu^2+^ ions display remarkable angiogenic and antibacterial activities and exert significant therapeutic effects on bone defects and bacterial infection 45. Based on mussel-inspired metal-phenol coordination chemistry, the Cu^2+^ ions released from MC nanosheets can chelate the abundant multivalent catechol/quinone groups in Alg-DA to form a pH-sensitive coordinate bond (catechol-Cu) via cation-π interactions, π-π stacking, electrostatic interactions and chelation coordination. pH-responsive coordination (catechol-Cu) endows the hydrogel with on-demand delivery of bioactive ions in an acidic environment **(Figure 3K)**. In addition, the formation of metal-phenol coordination networks also results in prominent antioxidant, antibacterial and anti-inflammatory effects, which play vital roles in accelerating bone regeneration 24. Therefore, we selected Cu^2+^ ions as a prototype biological agent to investigate the responsive release of Cu^2+^ from the GAD/MC hydrogel. Considering the inherent photothermal effect of the hydrogel, we investigated the thermos- and pH-responsive release properties of the GAD/MC hydrogel under various environmental conditions. As shown in **Figure 3L**, in the neutral physiological environment (pH = 7.4), a burst release of Cu^2+^ ions was observed in the initial 24 h, which tended to plateau with small fluctuations around a certain Cu^2+^ concentration in the following days, indicating that the release of Cu^2+^ was effectively prolonged under the combined action of hydrogel encapsulation and electrostatic and coordination systems. Moreover, the Cu^2+^ ion release kinetics were significantly accelerated under weakly acidic conditions at pH = 6.5, highlighting the pH responsiveness of the GAD/MC hydrogel to pathological microenvironments (inflammatory and acidic conditions). The burst release of Cu^2+^ can be ascribed to the accelerated dissociation of shallowly embedded MC nanosheets in acid, which induces a rapid increase in the concentration of Cu^2+^. The rapidly released Cu^2+^ ions coordinate with Alg-DA and form a dense film on the surface of the GAD/MC hydrogel, which prevents the permeation of the acidic solution into the hydrogel network, leading to an equilibrium of Cu^2+^ ions generated by MC nanosheet dissociation and the consumption of Cu^2+^ ions by coordination. On the other hand, the metal coordination bond between catechol and Cu^2+^ is unstable and can be destroyed under acidic conditions, which causes the rapid release of Cu^2+^ ions under acidic conditions, which is conducive to early vascularization and bacterial clearance. Additionally, the Cu^2+^ ion release performance of the GAD/MC hydrogel was also significantly enhanced after treatment with daily periodic NIR irradiation, which was mainly derived from the NIR-mediated photothermal effect that accelerated the degradation of the nanosheets. From the SEM images, it could be observed that the MC nanosheets loaded in the hydrogel matrix dissociated under the combined effects of weakly acidic conditions (pH = 6.5) and periodic NIR irradiation **(Figure S10)**, indicating their favorable removal ability, which facilitated the release of encapsulated bioactive components (i.e., Cu^2+^) and drugs. Additionally, the photothermal effect increases the temperature of the hydrogel, triggering shrinkage of the hydrogel network, which can also affect the release of Cu^2+^ ions in the hydrogel system. These findings further validated the stimuli-responsive drug release behavior of the GAD/MC hydrogel system under different pH conditions with/without NIR laser irradiation. Notably, bioactive Cu^2+^ ion release had therapeutic significance in this system. It can strengthen the antibacterial effect of mild photothermal treatment and accelerate angiogenesis to improve the therapeutic efficacy of bone defects. Based on the fact that the infectious or diabetic microenvironment was acidic, the injectable GAD/MC hydrogel released more Cu^2+^ ions in the acidic environment, which laid a solid foundation for achieving excellent antibacterial and vascularization efficacy through mild photothermal effects combined with Cu^2+^ release during the initial phase of bone repair. These advantages enabled GAD/MC to facilitate Cu^2+^ ion bioavailability and alleviate systemic adverse effects while maximizing its biofunction at the site of diabetic bone defects, thus potentially improving other stimuli-responsive scaffolds for bioactive ion delivery. Collectively, these experimental results proved that the release behavior of Cu^2+^ ions can be effectively tuned by both endogenous (e.g., low pH) and exogenous (e.g., mild heat) stimuli, showing the potential to dynamically adapt to the pathological microenvironment of bone defect sites, which is anticipated to achieve precise personalized treatment for diabetic bone defects even when infection and inflammation occur.

### *In vitro* antibacterial capability

Following severe trauma or other complex bone diseases, the repair of large-scale bone defects poses a formidable challenge in clinical practice due to the detrimental effects of heightened susceptibility to pathogen infection and excessive oxidative stress, resulting in deteriorated inflammation and impaired osteogenesis/angiogenesis 53. This phenomenon is more pronounced in patients with diabetes, where persistent chronic inflammation severely hampers bone regeneration and high levels of glucose contribute to a favorable environment for bacterial infection 52. In this context, it is crucial to construct a multifunctional hydrogel scaffold with outstanding antimicrobial and anti-inflammatory properties that are beneficial for accelerated bone healing under diabetic conditions.

**Figure 4:**
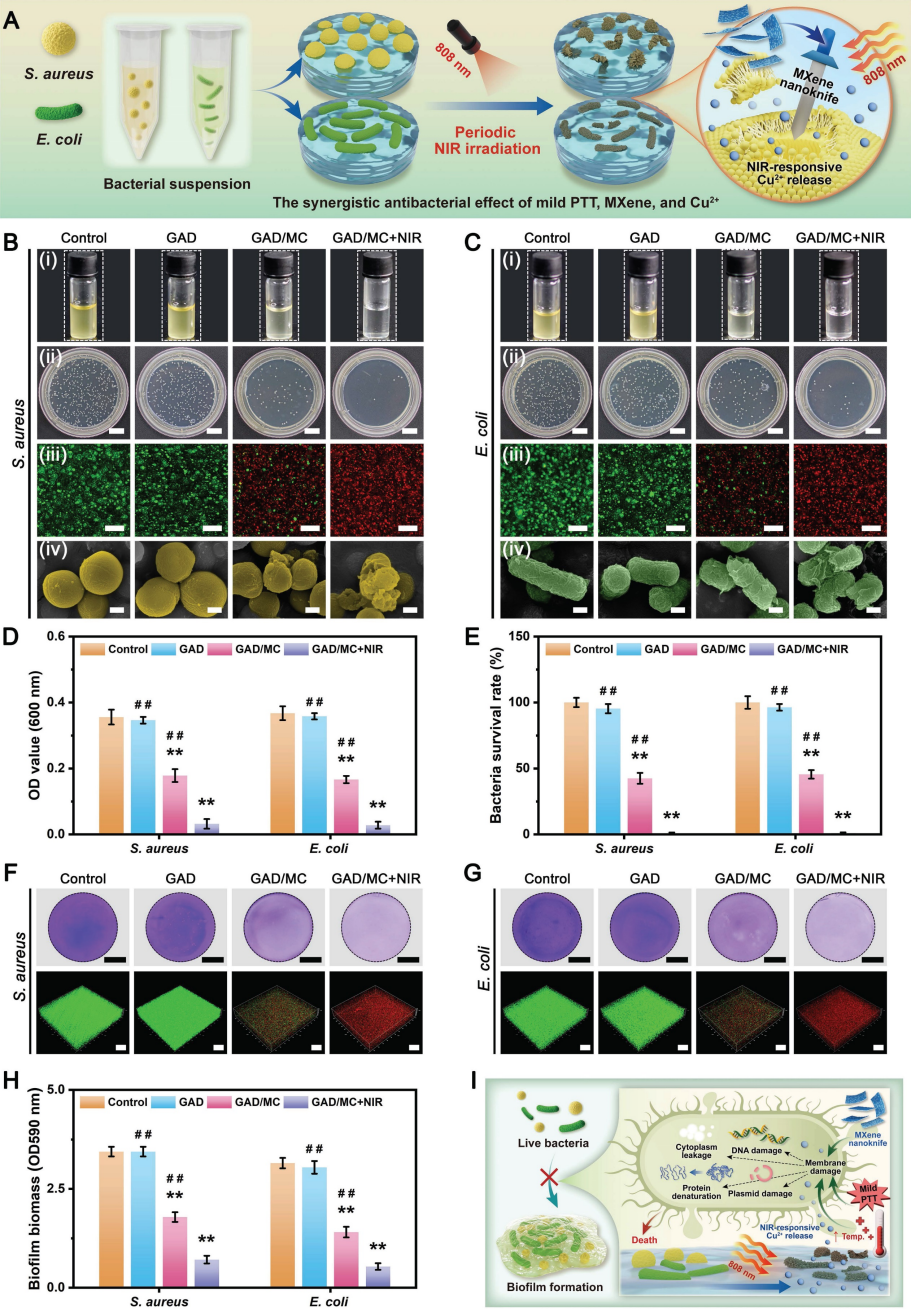
***In vitro* antibacterial performance of the mild photothermal-reinforced hydrogel system. (A)** Schematic illustration of the antibacterial mechanism of the photoactivated GAD/MC hydrogel system. **(B)** (i) Photographs of the *S. aureus* suspension after various treatments. (ii) Photographs of *S. aureus* colonies on agar plates after various treatments. Scale bar: 1 cm. (iii) Representative live/dead fluorescence images of *S. aureus* after various treatments. Scale bar: 50 μm. (iv) SEM images of *S. aureus* after various treatments. Scale bar: 300 nm. **(C)** (i) Photographs of the *E. coli* suspension after various treatments. (ii) Photographs of *E. coli* colonies on agar plates after various treatments. Scale bar: 1 cm. (iii) Representative live/dead fluorescence images of *E. coli* after various treatments. Scale bar: 50 μm. (iv) SEM images of *E. coli* after various treatments. Scale bar: 300 nm. **(D)** Quantitative analysis of bacterial viability for *S. aureus* and *E. coli* after various treatments. **(E)** Quantitative analysis of the bacterial survival rate after various treatments. **(F)** Representative images of crystal violet staining and 3D live/dead staining of *S. aureus* after different treatments. Scale bar: 3 mm (crystal violet staining images) and 100 μm (3D CLSM images).** (G)** Representative images of crystal violet staining and 3D live/dead staining of *E. coli* after different treatments. Scale bar: 3 mm (crystal violet staining images) and 100 μm (3D CLSM images).** (H)** Quantitative analysis of bacterial biofilms after different treatments. **(I)** Schematic illustration of the anti-biofilm mechanism of the photoactivated GAD/MC hydrogel system. Data are presented as the mean ± SD (n = 3). *P < 0.05 and **P < 0.01 indicate significant differences compared with the control group. ^#^P < 0.05 and ^# #^P < 0.01 indicate significant differences compared with the GAD/MC+NIR group.

Benefiting from the inherent antibacterial activity of Cu^2+^ ions, coupled with the nano-knife effect of the MXene nanosheets and mild hyperthermia, the photoactivated GAD/MC hydrogel was anticipated to be a broad-spectrum antibacterial platform **(Figure 4A)**. To verify our hypothesis, Gram-positive *Staphylococcus aureus* (*S. aureus*) and Gram-negative *Escherichia coli* (*E. coli*) were used for *in vitro* experiments because they are representative bacteria associated with bone infections 54. Subsequently, the antibacterial performance of the GAD/MC hydrogel with NIR light irradiation (GAD/MC+NIR) was comprehensively evaluated *in vitro* **(Figure S11)**. In the turbidimetric test **(Figure 4B(i)** and** Figure 4C(i)**), the culture medium in both the GAD/MC and GAD/MC+NIR groups was clearer than that in the GAD group, while the control group showed apparent turbidity due to the growth of *S. aureus* and *E. coli*. In particular, the bacterial suspension treated with the GAD/MC hydrogel plus NIR irradiation became transparent to a certain extent, which meant that the growth of *S. aureus* and *E. coli* was dramatically inhibited. The quantitative results revealed a significant reduction in the amplification rates of bacteria treated with the GAD/MC hydrogel, and this effect was even more pronounced with the combined application of periodic NIR irradiation **(Figure 4D)**. Simultaneously, treatment with the GAD hydrogel yielded limited inhibitory effects on bacterial growth, as evidenced by the turbidity being comparable to that of the control group. This observation indicated the potent antibacterial effect of the photoactivated GAD/MC hydrogel system against both bacterial strains. Similarly, the results of the spread plate experiment are shown in **Figure 4B(ii)** and** Figure 4C(ii)**, which also demonstrated the excellent antibacterial performance of the GAD/MC hydrogel against both *S. aureus* and *E. coli*. Specifically, compared with the control group, the GAD group exhibited no significant antibacterial activity against *S. aureus* or *E. coli*. In contrast, the antibacterial effect of the GAD/MC group was significantly improved, which can contribute to the inherent antibacterial property of the MXene nanosheets along with the release of Cu ions. In particular, there was a great bacterial reduction in the GAD/MC group in conjunction with mild NIR irradiation. Quantification of bacteria survival rate in the GAD/MC+NIR group was significantly lower than that in the GAD/MC and GAD groups **(Figure 4E)**, demonstrating that the GAD/MC+NIR group had the greatest ability to prevent the proliferation of *S. aureus* and *E. coli* on agar plates. Interestingly, the lower antibacterial efficacy of both the GAD/MC and GAD/MC+NIR groups on *E. coli* is primarily attributed to the slightly more negative surface charge of Gram-negative strains, contributing to their higher resistance to MXene nanosheets, which have negative surface charges, in agreement with the antibacterial results of other 2D nanomaterials 55. Moreover, as shown in **Figure 4B(iii)** and** Figure 4C(iii)**, the live/dead staining assay of bacteria indicated that there was a large amount of green fluorescence with negligible red fluorescence in the control and GAD groups, indicating that the proliferative activity of the bacteria was not affected. In contrast, a remarkable increase in red fluorescence and a substantial decrease in green fluorescence in the GAD/MC group indicated significant antibacterial activity. Notably, the fluorescence staining image of the GAD/MC group exposed to NIR irradiation exhibited nearly the strongest red fluorescence, demonstrating that a majority of the bacteria in the GAD/MC+NIR group were dead.

To further evaluate the membrane integrity and morphological changes of bacteria after different treatments, SEM observations were conducted. It could be seen in **Figure 4B(iv)** and** Figure 4C(iv)** that the bacteria in the control and GAD groups appeared normal, with smooth surfaces and intact cell membranes, indicating that the bacteria were not significantly damaged.

Conversely, bacteria exposed to GAD/MC hydrogel treatment underwent obvious morphological alterations, including cell membrane rupture, crumpling, and loss of cellular integrity. Notably, in the presence of NIR irradiation, both bacteria exhibited irregular morphological shrinkage and severe cytoplasm leakage, indicating that the integrity of the bacteria was severely damaged, leading to leakage of contents and protein denaturation. The strong antibacterial performance of the GAD/MC+NIR group was primarily due to its excellent photothermal properties and the synergistic effect of antibacterial substances such as MXene nanosheets and Cu^2+^ ions 27, 46.

Since bacterial biofilm formation is an important cause of persistent infection in the clinic that significantly prevents osseointegration and delays bone healing, developing bone repair scaffolds with good antibacterial properties and anti-biofilm formation capacity for treating diabetic bone defects is urgently needed. Encouraged by the above promising antimicrobial experiments, we further investigated the inhibitory efficacy of the hydrogel system against *S. aureus* and *E. coli* biofilms by crystal violet staining and 3D confocal laser scanning microscope (CLSM) observation. As shown in **Figure 4F-G**, a continuous biofilm layer with high integrity was formed in the control and GAD groups, exhibiting an active living status (green fluorescence) with a dense structure. In contrast, the introduction of MC into the hydrogel caused biofilm damage and bacterial apoptosis (displaying red fluorescence) in the GAD/MC group. Notably, the biofilm was severely disrupted after GAD/MC+NIR treatment, with thin and dispersed features. Meanwhile, intense red fluorescence was observed in the biofilm treated with GAD/MC+NIR, demonstrating its tremendous potential against *S. aureus* and *E. coli*, which was confirmed with a spread plate experiment and SEM observation. The quantitative results of crystal violet staining verified that the GAD/MC hydrogel could damage the biofilm structure to a large extent under NIR irradiation **(Figure 4H)**, exhibiting the highest efficacy in eradicating biofilms. These findings underscore the inhibitory impact of the combined action of MXene nanosheets, Cu^2+^ ions, and local mild hyperthermia on bacterial biofilms, resulting in the disruption of biofilm structure and bacterial killing. Furthermore, emerging studies have corroborated the synergistic antibacterial and anti-biofilm effects of mild PTT combined with sustained Cu^2+^ delivery 53, 56, further supporting our present findings. In this context, multiple bactericidal mechanisms stemming from mild photothermal-reinforced hydrogel systems are elucidated in **Figure 4I**. First, benefiting from the intrinsic antibacterial properties of MXene and Cu^2+^, the hydrogel may interact with the cell wall through electrostatic interactions as well as through physical damage to the bacterial membrane structure, leading to impaired membrane function and nutrient assimilation. Subsequently, in the presence of on-demand NIR irradiation, the photoactivated hydrogel system initiated mild PTT that can amplify the activity of metal ions (i.e., Cu^2+^) via the “mild hot ions effect”, showing high-efficiency, quick, and long-term inhibition of bacteria, which played a synergistic antibacterial role in combating bacterial infections. All these findings supported the strong antibacterial activity of the rationally designed GAD/MC hydrogel for inhibiting the colony growth, bacterial proliferation, and biofilm formation of *S. aureus* and *E. coli*.

### *In vitro* biocompatibility and osteogenic potential

**Figure 5:**
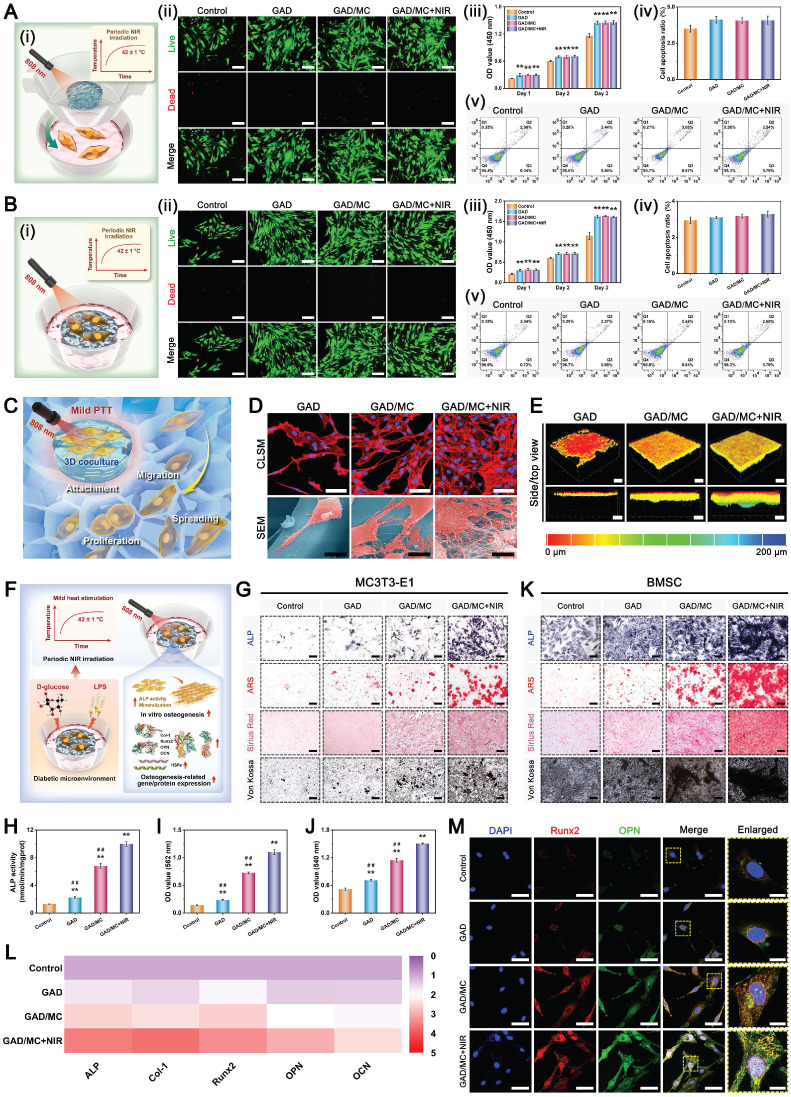
***In vitro* biocompatibility and osteogenic potential of the mild photothermal-reinforced hydrogel system. (A)** (i) Schematic illustration of the Transwell coculture system. (ii) Representative live/dead staining images of MC3T3-E1 cells after different treatments for 3 days. Scale bar: 200 μm. (iii) Cell proliferation of MC3T3-E1 cells in different groups by the CCK-8 assay after 1, 2, and 3 days of coculture. (iv-v) Representative flow cytometry images and apoptosis analysis of MC3T3-E1 cells in different groups after 3 days of coculture. **(B)** (i) Schematic illustration of the direct coculture system. (ii) Representative live/dead staining images of MC3T3-E1 cells after different treatments for 3 days. Scale bar: 300 μm. (iii) Cell proliferation of MC3T3-E1 cells in different groups by the CCK-8 assay after 1, 2, and 3 days of coculture. (iv-v) Representative flow cytometry images and apoptosis analysis of MC3T3-E1 cells in different groups after 3 days of coculture.** (C)** Schematic illustration of the *in vitro* 3D coculture model. **(D)** CLSM and SEM images of MC3T3-E1 cells after different treatments for 3 days. Scale bar: 50 μm (CLSM images) and 10 μm (SEM images). **(E)** 3D CLSM images of MC3T3-E1 cells distributed within the hydrogels. Scale bar: 100 μm. **(F)** Schematic illustration of the osteogenic differentiation of MC3T3-E1 cells *in vitro*. **(G)** Representative images of ALP, ARS, Sirius Red, and Von Kossa staining of MC3T3-E1 cells after different treatments. Scale bar: 200 μm.** (H-J)** Quantitative analysis of ALP activity, ECM mineralization, and collagen secretion. **(K)** Representative images of ALP, ARS, Sirius Red, and Von Kossa staining of BMSCs after different treatments. Scale bar: 200 μm.** (L)** Relative mRNA expression of osteogenesis-related genes in MC3T3-E1 cells after different treatments. **(M)** Representative images of immunofluorescence staining for Runx2 and OPN in MC3T3-E1 cells. Scale bar: 50 μm (low-magnification images) and 10 μm (high-magnification images). Data are presented as the mean ± SD (n = 3). *P < 0.05 and **P < 0.01 indicate significant differences compared with the control group. ^#^P < 0.05 and ^# #^P < 0.01 indicate significant differences compared with the GAD/MC+NIR group.

As a promising biomaterial scaffold to facilitate bone regeneration, the as-prepared hydrogel system should possess good cytocompatibility and osteogenic activity. In this study, calvaria-derived MC3T3-E1 preosteoblasts were used as model cells because of their stable performance and good experimental reproducibility 57. The viability and proliferation of MC3T3-E1 cells with or without NIR irradiation were assessed in both direct contact and indirect contact coculture models **(Figure 5A-B)**. According to the results of live/dead staining and the CCK-8 assay, the MC3T3-E1 cells maintained good viability and proliferation rates during the entire experimental period, with almost identical growth rates in all experimental groups and higher growth rates than those of the control group, suggesting desirable cytocompatibility *in vitro*. Furthermore, after 3 days of coculture, cell proliferation in all experimental groups outperformed that of the control group without hydrogels, indicating that the hydrogels prepared in this work could favor cell proliferation and that appropriate NIR hyperthermia would not cause significant toxicity. The effective promotion of cell proliferation was likely attributed to the release of nutrients and bioactive Cu^2+^ caused by the degradation of organic macromolecules and nanosheets. Similar tendencies were also found by flow cytometry and corresponding quantitative analysis, with apoptosis rates below 5% under all conditions, suggesting negligible cytotoxicity in all experimental groups. Previous studies reported that a high concentration of antimicrobial agents, including Cu^2+^, within the biomaterial scaffold would produce cytotoxicity, limiting their practical application in the clinic 54. Herein, we demonstrated that the cytotoxic effects of Cu^2+^ could be effectively mitigated by adsorbing them in MXene nanosheets through tight electrostatic interactions, followed by hydrogel encapsulation and ionic crosslinking as well as metal coordination between catechol and Cu^2+^. Many studies have reported methods for encapsulating Cu^2+^, such as covering it with polymers or mixing it directly into surface coatings 46. Compared with these strategies, our multiple-crosslinking strategy realized the controlled and stimuli-responsive release of Cu^2+^, which helped to decrease toxicity while maintaining long-term biological activity. Taken together, these results suggest that the incorporation of MC nanosheets in hydrogels and the synergistic application of periodic NIR irradiation are appropriate and safe for cell proliferation and growth, thus meeting the requirements for their potential application as bone repair scaffolds in clinical settings.

After bone injury or scaffold implantation, the effective promotion of osteoblast adhesion, migration, recruitment, spreading, and differentiation within defect areas are essential processes involved in bone repair and remodeling. After irradiation with NIR laser (808 nm, 1.5 W/cm^2^), the temperature of the cell-laden GAD/MC hydrogel reached about 42 °C within 5 min **(Figure S12)**, which meets the anticipated requirements for mild PTT 42. Herein, the attachment and morphology of MC3T3-E1 cells cultured on hydrogels for 5 days were comparatively analyzed by SEM and CLSM **(Figure 5C)**. As shown in **Figure 5D,** the hydrogels were capable of effectively supporting cell adhesion and spreading, with the cells displaying apparent elongation and F-actin filament extension. Remarkably, the GAD/MC group showed abundant elongated filopodia and pseudopodia, and the number of these osteoblasts was increased compared with the GAD group, indicating strong cell-cell interactions. Such intercellular interactions favor intercellular signal transmission and further cell differentiation 58. Furthermore, this characteristic performance was more obvious after treatment with periodic NIR irradiation, characterized by the formation of a high density of homogeneous cell layers in the GAD/MC+NIR group. Compared with those in the GAD and GAD/MC groups, cells in the GAD/MC+NIR group developed a more well-spread morphology with prominent elongated microfilaments and filamentous extensions, which could effectively transmit ECM signals into the cell interior to regulate cell behavior and tissue development, implying potent pro-osteogenic potential. Quantitative analysis further revealed the largest spreading area in the GAD/MC+NIR group, followed by the GAD/MC group and the GAD group **(Figure S13)**. The strong interaction between MC3T3-E1 cells and the hydrogel system might be correlated with the highly ECM-mimicking porous structure, rough surface morphology, DA-induced cell adhesion, and mild heat stimulation, which provided favorable anchoring sites and physical cues for cell adhesion and spreading. Cell migration and recruitment are essential for tissue regeneration because they facilitate the recruitment of stem cells to the injured site and participate in tissue remodeling. To assess cell migration, osteoblasts were incubated on hydrogel surfaces, and their migration from the upper surface into the hydrogel interior was examined using cytoskeleton staining after coculture and observed using CLSM **(Figure 5E)**. The results of 3D scanning images revealed that the GAD/MC group displayed increased cell penetration depth and cell density compared to those of the GAD group, while the GAD/MC+NIR group showed even better performance, indicating remarkable cellular ingrowth into the hydrogel system. This difference was attributed to MC incorporation and mild heat stimulation, which led to more structural and physiological cues, thus providing guidance for the migration and spatial organization of osteoblasts within the 3D hydrogel matrix. Notably, periodic and long-term local mild heat stimulation has been clinically proven to be beneficial for stimulating cell biomineralization and osteogenesis, further affecting the process of bone regeneration, including osteoblast attachment and migration and osseointegration 12. In addition, hydrophilicity and mechanical properties can impact cell adhesion, spreading, and osteoblastic differentiation. In this study, compared to those in the GAD hydrogel, the inorganic/organic interweaved nanostructure in the GAD/MC hydrogel markedly increased the hydrophilicity and mechanical properties of the hydrogel, which may be one of the reasons for the rapid migration and infiltration of cells. In summary, these observations strongly suggested that the photoactivated hydrogel system promoted osteoblast adhesion, spreading, and migration via various physical and exogenous stimuli.

With respect to diabetic bone defects, chronic inflammation and high glucose in bone tissue inhibit osteoblast differentiation, leading to bone loss and impaired bone formation in individuals with DM 7. Thus, enhancing the osteogenic differentiation capacity under the DM-related microenvironment can improve bone health and reverse abnormal osteogenic activity. To mimic the diabetic environment characterized by aggravated inflammation and high glucose, LPS (200 ng/mL) and D-glucose (25 mM) were added to the culture medium 11, 59. The ability of the hydrogel system to stimulate the osteogenic differentiation behavior of MC3T3-E1 preosteoblasts was evaluated by measuring ALP activity, collagen secretion, ECM mineralization, and calcium deposition *in vitro* **(Figure 5F)**. ALP, an early marker of osteogenic differentiation, plays a critical role in the mineralization process by regulating Ca^2+^ and PO_4_^3-^ metabolism. Simultaneously, collagen secretion, ECM mineralization, and calcium deposition occur mainly at the later stages of osteogenic differentiation 60. As displayed in **Figure 5G**, more pronounced coloration was observed in the hydrogel-treated groups than in the control group, suggesting enhanced osteoinductivity. Compared with the control and GAD groups, the GAD/MC hydrogel-treated group exhibited higher ALP activity and ECM mineralization as well as more collagen secretion and calcium deposition, indicating the synergistic osteogenesis-promoting effects of DA functionalization and MC incorporation. Importantly, the osteogenic potential of MC3T3-E1 cells was further strengthened by the mild heat stimulation induced by periodic NIR irradiation, which suggested that MC-mediated mild hyperthermia promoted osteoblast differentiation. For 2D nanomaterials, existing evidence suggests that Ti_3_C_2_T_x_ MXene nanosheets have intrinsic osteoinductive activity and can synergize with mild heat stimulation to collectively enhance osteoblast function 23. In addition, quantitative analysis also validated the superior osteoinductive potential of the hydrogel system **(Figure 5H-J)**. Building upon our previous experimental findings, we further explored the influence of the GAD/MC hydrogel system on the osteogenic differentiation of BMSCs *in vitro*. Rat BMSCs extracted from rat bone marrow were cultured in accordance with a previously described protocol 61, 62. Compared with those in the control group, prominent improvements in ALP activity, collagen secretion, and mineralized nodule formation were observed in the GAD group, while the GAD/MC group further effectively enhanced the osteogenic function of BMSCs **(Figure 5K)**, which was consistent with the trend observed in MC3T3-E1 cells. Notably, with the help of NIR-mediated mild PTT, the GAD/MC hydrogel-treated group displayed the highest osteogenic activity and biomineralization capability among all the groups in the hyperglycemic and inflammatory environments, indicating enhanced osteogenic differentiation and mineralization of the BMSCs. These findings aligned with the previous notion that on-demand mild hyperthermia can facilitate cell spreading and cytoskeleton reorganization to modulate the osteogenic differentiation and biomineralization of MSCs 63. Meanwhile, osteogenic gene expression was evaluated, and the qRT-PCR results demonstrated that the GAD/MC hydrogel combined with mild heat stimulation significantly upregulated the expression of osteogenesis-related genes (ALP, Col-1, Runx2, OPN, and OCN) to the greatest extent under diabetic inflammatory conditions (**Figure 5L**). The improved osteogenic differentiation capability was also supported by the results of the immunofluorescence staining assay. As shown in **Figure 5M**, the expression levels of osteogenic markers (Runx2 and OPN) were profoundly upregulated in cells cocultured with GAD/MC hydrogels, especially after being subjected to periodic NIR irradiation. Quantitative statistics further proved that the GAD/MC+NIR group displayed increased fluorescence intensities of Runx2 and OPN in comparison with those of all the other groups **(Figure S14)**. These results revealed that the levels of osteogenic markers in the GAD/MC hydrogel-treated groups were higher than those in the GAD and control groups at both the protein and gene levels, especially for the GAD/MC+NIR group, which could be ascribed to the combined effects of MC nanosheets and NIR-assisted mild hyperthermia. Similarly, the immunofluorescence staining of Runx2 and OPN in the BMSCs further demonstrated that the osteogenic capability significantly increased with the aid of mild photothermal treatment in the GAD/MC group **(Figure S15)**. As previously reported, mild heat stimulation, an important biophysical cue, can promote osteogenic differentiation and bone formation by activating the heat shock response in osteogenesis-related cells, upregulating HSPs, and stimulating the downstream ERK-Wnt/β-catenin signaling pathway 14. The same finding was further verified in our present study, showing that the expression levels of HSP47 and HSP70 were predominantly upregulated in cells cocultured with the GAD/MC hydrogel under NIR irradiation, whereas the expression levels of these genes in both the GAD/MC and GAD groups were comparable to those in the control group **(Figure S16)**. In addition, other physical properties (hydrophilicity and mechanical strength) and active components (DA, Cu^2+^, active proteins, and glycoproteins) may also be conducive to osteogenesis, leading to increased expression levels of osteogenesis-related markers. Based on the synergistic effects of mild heat stimulation and Cu^2+^ delivery, improved mechanical properties, remarkable injectability and moldability, and adhesive abilities, NIR/pH dual-responsiveness, biocompatibility, and outstanding antibacterial and osteogenic activities, the GAD/MC hydrogel platform offers a new perspective for expediting bone defect repair and healing under pathological diabetic conditions.

### *In vitro* antioxidant and anti-inflammatory activities

**Figure 6:**
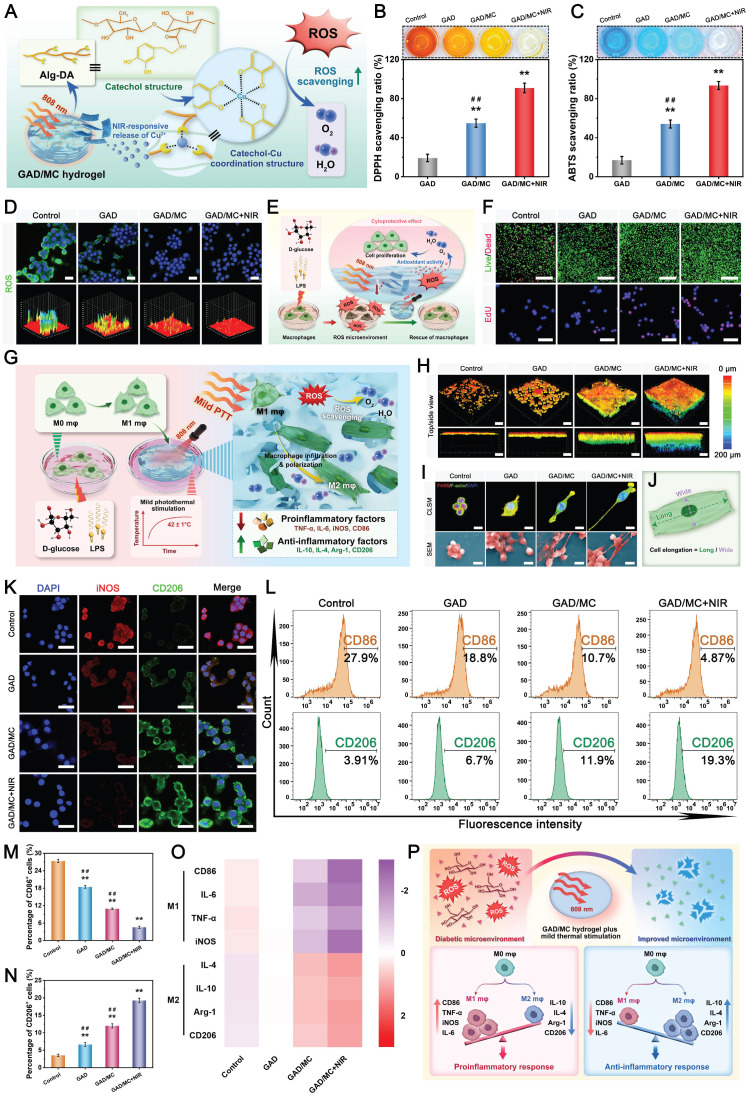
** Antioxidant and anti-inflammatory activities of the mild photothermal-reinforced hydrogel system. (A)** Schematic illustration of the antioxidant mechanism of the photoactivated GAD/MC hydrogel system. **(B-C)** DPPH and ABTS scavenging activity of the hydrogels. Data are presented as the mean ± SD (n = 3). *P < 0.05 and **P < 0.01 indicate significant differences compared with the GAD group. ^#^P < 0.05 and ^# #^P < 0.01 indicate significant differences compared with the GAD/MC+NIR group. **(D)** Intracellular ROS scavenging assay and corresponding 3D surface plot images of RAW264.7 cells under LPS and D-glucose stimulation. Scale bar: 25 μm.** (E)** Schematic illustration of the cytoprotective effect of the photoactivated GAD/MC hydrogel system on RAW264.7 cells under LPS and D-glucose stimulation. **(F)** Representative images of live/dead staining and EdU staining of RAW264.7 cells under LPS and D-glucose stimulation. Scale bar: 200 μm (live/dead staining images) and 50 μm (EdU staining images). **(G)** Schematic illustration of the immunomodulatory mechanism of the photoactivated GAD/MC hydrogel system. **(H)** 3D CLSM images of RAW264.7 cells distributed within the hydrogels. Scale bar: 100 μm. **(I)** CLSM and SEM images of RAW264.7 cells after different treatments for 3 days. Scale bar: 5 μm (CLSM images) and 10 μm (SEM images). **(J)** Schematic diagram of the calculation of macrophage elongation. **(K)** Representative images of immunofluorescence staining for iNOS and CD206 in RAW264.7 cells after different treatments. Scale bar: 25 μm. **(L)** Flow cytometry and **(M-N)** quantitative analysis of the cell surface markers CD86 and CD206 in RAW264.7 cells after different treatments.** (O)** Relative mRNA expression of inflammation-related markers in RAW264.7 cells after different treatments for 3 days. **(P)** Schematic illustration of diabetes-related complications leading to an inflammatory response and the effect of the photoactivated GAD/MC hydrogel system on the immune microenvironment balance. Data are presented as the mean ± SD (n = 3). *P < 0.05 and **P < 0.01 indicate significant differences compared with the control group. ^#^P < 0.05 and ^# #^P < 0.01 indicate significant differences compared with the GAD/MC+NIR group.

An increasing amount of research has demonstrated that diabetic bone defects are more susceptible to oxidative stress than normal bone defects without comorbidities 3. In the diabetic milieu, excessive intracellular mitochondrial ROS catalyze oxidative damage to cytomembranes, nucleic acid, and DNA, disrupting cellular equilibrium and promoting apoptosis, ultimately causing a vicious cycle of inflammation and oxidative stress. Uncontrolled oxidative stress and persistent inflammation in local defect areas ultimately contribute to the loss of bone tissue homeostasis and the decrease in regenerative potential 64. Therefore, the development of an injectable hydrogel integrated with ROS scavenging capabilities holds promise for maintaining the cellular redox balance and achieving optimal regeneration outcomes under DM conditions **(Figure 6A)**. Encouragingly, both polyphenol group-containing materials (e.g., Alg-DA) and 2D MXene nanosheets have recently been reported to possess excellent ROS-scavenging capacities 13, 32, making them promising candidates for treating diabetic bone defects. To verify our hypothesis, the antioxidant capacity of the prepared GAD/MC hydrogel system was first evaluated by 2,2-diphenyl-1-picrylhydrazyl (DPPH) and 2,2′-azino-bis (3-ethylbenzothiazoline-6-sulfonic acid) (ABTS) radical scavenging assays using a biological oxidative stressor, H_2_O_2_, as the catalytic substrate **(Figure 6B-C)**. Compared with the control or GAD group, the GAD/MC group demonstrated excellent free radical scavenging ability, and this effect was enhanced with the assistance of mild PPT (above 90%). The potent free radical scavenging ability of the GAD/MC+NIR group was mainly attributed to the synergistic effect of Alg-DA and Ti_3_C_2_ MXene. Specifically, ROS were eliminated by the antioxidant phenol quinone groups in Alg-DA chains via redox reactions, which was further reinforced by the outstanding electron transmission ability of the Ti_3_C_2_ MXene 6. Remarkably, the synergistic utilization of mild NIR irradiation could accelerate the formation of new coordination interactions between Cu^2+^ and Alg-DA and facilitate adequate contact between reductive components and free radical detection reagents, thereby significantly enhancing the antioxidant capacity of these compounds to scavenge ROS.

Multiple studies have demonstrated that the pathological microenvironment in diabetes commonly involves the overproduction of ROS induced by high levels of glucose and persistent inflammation 65. Due to the presence of DA and MXene nanosheets, we speculated that the prepared hydrogels have the potential to efficiently scavenge intracellular ROS to mitigate oxidative stress and protect cells against damage in diabetic conditions. To validate these findings, macrophages (RAW264.7) were stimulated with LPS (200 ng/mL) and D-glucose (25 mM) to simulate local hyperglycemia and the inflammatory environment after bone injury in individuals with diabetes. Subsequently, intracellular ROS production in response to LPS and D-glucose was quantified by 2′,7′-dichlorofluorescein diacetate (DCFH-DA, a probe for staining ROS) and malondialdehyde (MDA) assays. As can be seen in **Figure 6D** and **Figure S17A**, in contrast to the control group, which exhibited intense green fluorescence, a significant decrease in the number of ROS^+^ cells was observed in the hydrogel-treated groups, especially the GAD/MC group. This phenomenon might be explained by the combined effects of MXene incorporation and the quinone/catechol-rich structure of DA 66, which could efficiently reduce the elevated intracellular ROS levels induced by LPS and D-glucose. Previous studies, including ours, have consistently reported that MXene nanosheets have excellent free radical scavenging ability, including hydrogen peroxide (H_2_O_2_), hydroxyl radicals (•OH), and superoxide anions (•O_2_^-^), which helps maintain the cellular redox balance and control inflammation 13, 23. It was worth noting that the application of mild NIR irradiation achieved the best antioxidant performance, as confirmed by the further decrease in DCFH-DA signal intensity within cells. The efficiency of ROS elimination was evaluated quantitatively via flow cytometry analysis; the lowest mean fluorescence intensity was achieved in the group treated with the GAD/MC hydrogel plus mild NIR irradiation, corroborating the DCFH-DA staining results. The excellent ability of the photoactivated hydrogel system to scavenge intracellular ROS was mainly derived from the synergistic effect of the accelerated electron movement in the MXene and quinone/catechol-rich structure of Alg-DA, as well as the formation of new catechol-metal coordination bonds between Cu^2+^ and Alg-DA 44. Simultaneously, MDA is the final product of lipid peroxidation, and its level can reflect the lipid peroxidation level induced by ROS. Here, a lipid peroxidation MDA assay kit was used to measure the MDA content in the macrophages. Moreover, treatment with the GAD/MC hydrogel plus NIR irradiation remarkably downregulated the elevated MDA level in macrophages subjected to LPS and D-glucose **(Figure S17B)**, revealing the photothermal-enhanced ROS scavenging capacity of the hydrogel system. As mentioned earlier, excessive accumulation of ROS within the diabetic bone defect microenvironment can heighten cellular oxidative stress and impair cellular activity. Encouraged by the above results, the as-prepared GAD/MC hydrogel system was expected to maintain a favorable redox environment and offer superior protection against intracellular ROS generation **(Figure 6E)**. To further validate our hypothesis, mouse macrophages (RAW264.7) were used to examine the cytoprotective properties of the GAD/MC hydrogel system against overproduced ROS *in vitro*. The results of live/dead staining, 5-ethynyl-2′-deoxyuridine (EdU) staining, and CCK-8 assays confirmed the remarkable cytoprotective effect of the hydrogel system, and this ability was further reinforced with the aid of NIR-induced mild heat stimulation, as evidenced by the increased cell viability and proliferation rate compared with those of the control group **(Figure 6F** and **Figure S18)**. LPS and D-glucose stimulation led to decreased cell viability and a large number of dead cells (red fluorescence), mainly due to the killing effect caused by persistently elevated levels of proinflammatory cytokines and overexpressed ROS generated in DM-related conditions. Conversely, an increased number of live cells (green fluorescence) appeared in the hydrogel-treated groups. Among these groups, the GAD/MC+NIR group showed the highest green fluorescence (live cells) and the best cell density, followed by the GAD/MC and GAD groups, which reached a higher level than that of cells without any treatment. Notably, by reversing the elevated ROS levels *in situ*, the GAD/MC+NIR group demonstrated significant cytoprotective effects compared to the GAD/MC and GAD groups, which was consistent with our hypothesis. We speculate that the therapeutic mechanism may be closely linked to the combined effects of multiple DA/MXene-mediated ROS-scavenging activities, the formation of catechol-metal coordination, and mild PTT, which jointly improve cell metabolism and growth by reducing mitochondrial dysfunction and ROS production 16, thereby shielding these cells from the impact of diabetic pathological conditions. These *in vitro* results clearly manifested that the hydrogel system, in combination with mild PTT, possessed the capability to protect cells against exogenous oxidative stress by scavenging excessive ROS. Recent studies have shown that both DA- and MXene-functionalized hydrogels exhibit multiple ROS scavenging activities, which can help maintain redox homeostasis to alleviate cellular damage under oxidative stress 23, 44. Because of the inherent biological properties of polyphenols, a mussel-inspired molecule, i.e., DA, possesses favorable antioxidant properties because the catechol moiety in DA can eliminate ROS while being oxidized to quinone moieties 67. Mounting evidence has also demonstrated that 2D transition metal carbides (MXenes) have excellent reducibility and catalase (CAT)-like and superoxide dismutase (SOD)-like activities and can exert synergistic effects on reducing intracellular ROS and inhibiting oxidative stress damage 28. Taken together, these findings revealed that our prepared GAD/MC hydrogel system, which has excellent antioxidant activity, could improve cell viability and protect cells from diabetes-induced cell damage or death by eliminating intracellular ROS, which provides strong evidence supporting its further application for bone repair under diabetic conditions.

For bone defect repair under diabetic conditions, local injury sites fail to transit smoothly from the inflammatory phase to the repair and remodeling phases due to macrophage dysfunction, resulting in a persistent proinflammatory environment 64. Moreover, the local inflammatory milieu within the defective area, accompanied by the production of excessive ROS, could induce reprogramming of the infiltrated macrophages toward a proinflammatory M1 phenotype, which further secretes proinflammatory cytokines to recruit and polarize macrophages, creating a positive feedback loop to exacerbate the pathological conditions of bone defects. These findings indicate that modulating the macrophage phenotype to alter the immune microenvironment could alleviate the inflammatory state within the defect area and expedite the inflammatory phase transition to subsequent repair and remodeling phases, which is beneficial for promoting *in situ* bone regeneration 59. Considering its efficient antioxidant and ROS-scavenging activities as well as favorable cytoprotective capacity, we further assessed the effect of the hydrogel system on *in vitro* macrophage infiltration and polarization **(Figure 6G)**. The infiltration of recruited macrophages is an important step for the implanted material to interact with native tissue and mediate the host foreign body response 68. 3D reconstructed confocal images showed that the seeded macrophages only adhered to the surface of the tissue culture plate (TCP) (at a depth of less than 40 μm), revealing a typical 2D growth pattern. In contrast, the cells could not only adhere and distribute on the hydrogel surface but also migrate downward to the bottom of the GAD (about 60 μm in depth) and the GAD/MC (about 120 μm in depth) **(Figure 6H)**. Most importantly, upon exposure to periodic NIR irradiation, remarkable cellular infiltration into the GAD/MC hydrogel (about 200 μm in depth) was found in the 3D restructuration images. These contrasting findings showed that the GAD/MC hydrogel system is beneficial for promoting cell infiltration and penetration, thus facilitating the interaction between macrophages and implanted materials and regulating subsequent tissue remodeling, aided by mild heat stimulation.

To investigate the anti-inflammatory potential of the GAD/MC hydrogel system, RAW264.7 cells were stimulated with LPS and D-glucose to induce a proinflammatory M1 phenotype. As shown in** Figure 6I**, the macrophages treated with LPS and D-glucose exhibited a round shape and extensive pseudopodia, which was in accordance with the characteristics of proinflammatory M1 macrophages 57. A recent study reported that M0/M1 macrophages present a round shape and extensive pseudopodia, while M2 macrophages are elongated, spindle-shaped, and spread better 40. The results of SEM images also verified the successful activation of proinflammatory M1 macrophages. In contrast, macrophages cocultured with the hydrogels appeared as apparently elongated and flattened spindle-like cells with F-actin filament extension, and this morphological feature was more pronounced following treatment with the GAD/MC hydrogel plus daily periodic NIR irradiation. Consistently, a higher cell aspect ratio was detected in the GAD and GAD/MC groups than in the control group, and this stimulation effect was significantly enhanced following periodic NIR irradiation (**Figure 6J** and **Figure S19)**. It has been demonstrated that elongated spindle-shaped macrophages with a high spreading area and filopodia prefer an activated anti-inflammatory macrophage phenotype and induce high expression of the anti-inflammatory factors IL-4 and IL-10 for optimal bone regeneration 69. Hence, these findings suggested that the GAD/MC hydrogel combined with NIR-assisted mild heat stimulation could jointly facilitate macrophage infiltration elongation and spreading, showing the potential of this hydrogel platform for inducing macrophage polarization toward an anti-inflammatory M2 phenotype *in vitro*. Subsequently, immunofluorescence staining and flow cytometry analysis revealed that the control group exhibited a persistent inflammatory state characterized by increased M1 macrophages and elevated levels of proinflammatory mediators, as well as a spike in intracellular ROS levels. As shown in **Figure 6K-L**, macrophages were first stimulated with LPS and D-glucose, wherein an apparent red fluorescence of iNOS was detected. In contrast to the macrophages in the control group, those in the GAD and GAD/MC groups presented a significantly increased ratio of M2 macrophages (CDCD206^+^), accompanied by a decreased percentage of M1 macrophages (CD86^+^). In particular, the optimal anti-inflammatory effect was achieved when combined with periodic NIR irradiation, as evidenced by the significantly elevated expression of CD206 and decreased expression of iNOS in the GAD/MC+NIR group **(Figure 6M-N)**. Both the CLSM and flow cytometry results suggested that the hydrogel system combined with mild PTT could effectively accelerate the transition of macrophages from the proinflammatory (M1) phenotype to the anti-inflammatory (M2) phenotype under the DM environment. To further verify these results, the expression levels of inflammation-related markers in macrophages were quantified at the gene level. The downregulation of M2-related markers, including IL-4, IL-10, Arg-1, and CD206, was detected in cells exposed to LPS and D-glucose, whereas upregulation of these genes was observed after treatment with the hydrogels **(Figure 6O)**. Notably, macrophages treated with the GAD/MC hydrogel showed a significant increase in the expression of M2 phenotype markers, and a further increase was evident in the presence of mild NIR irradiation, while the mRNA levels of M1 macrophage markers, including CD86, IL-6, TNF-α, and iNOS, exhibited opposite results.

These findings are in agreement with the data obtained from the abovementioned immunofluorescence staining and flow cytometry analysis, indicating that the photoactivated hydrogel system effectively suppressed M1 polarization of macrophages following LPS and D-glucose stimulation while simultaneously promoting the polarization of macrophages toward the regenerative M2 phenotype. Extensive studies have demonstrated that mild heat stimulation and DA functionalization can induce M2 macrophage polarization, which in turn regulates the osteoimmune microenvironment by promoting anti-inflammatory cytokine secretion 16, consistent with our present *in vitro* data. Additionally, other studies have reported that surface nanotopography cues from biomaterials influence macrophage polarization by regulating cell morphology 70, 71. In this study, the GAD/MC hydrogel exhibited a nanoroughed surface, while the GAD hydrogel had a relatively smooth surface. The nanoscale roughness of the surface caused by the MC nanosheets in the GAD/MC hydrogel exerts a synergistic effect on inducing cell filopodia formation, resulting in the distinct expression of integrins and focal adhesion (FA) molecules that can modulate macrophage polarization. Furthermore, intracellular ROS levels have been found to play a crucial role in the process of inflammation and macrophage polarization, and reduced ROS levels at sites of bone defects were more inclined to promote the reprogramming of macrophages to the M2 phenotype. Consequently, our findings showed that the GAD/MC hydrogel combined with NIR-assisted mild PTT effectively scavenged ROS in DM-activated macrophages, which was expected to reverse the continuous inflammatory reaction by promoting M2 macrophage polarization under diabetic conditions **(Figure 6P)**. To further examine the effect of the hydrogel system on the inflammatory response, an ELISA was used to quantify inflammatory factors in RAW264.7 cells after various treatments. Compared with those in the control group, the treatment with the hydrogels, especially the GAD/MC hydrogels, were shown to dramatically downregulate proinflammatory cytokines (IL-4 and IL-1rα), and upregulated anti-inflammatory cytokines (IL-1β and IL-6), and these effects became more obvious after being subjected to periodic NIR irradiation **(Figure S20)**. This result was consistent with the above immunofluorescence staining, suggesting that more obvious M2 macrophage polarization and anti-inflammatory cytokine secretion were induced in the GAD/MC and GAD/MC+NIR groups than in the other groups, with the latter showing superior effects. On the basis of the above analysis, our proposed GAD/MC hydrogel system could attenuate hyperglycemia and the inflammatory environment-mediated proinflammatory response while promoting pro-regenerative M2 macrophage activation and inducing the expression of immunomodulatory cytokines most efficiently, which would provide obvious benefits in the restoration of the proper immune environment and subsequent osteo/angiogenesis induction.

### *In vitro* angiogenic activity

**Figure 7:**
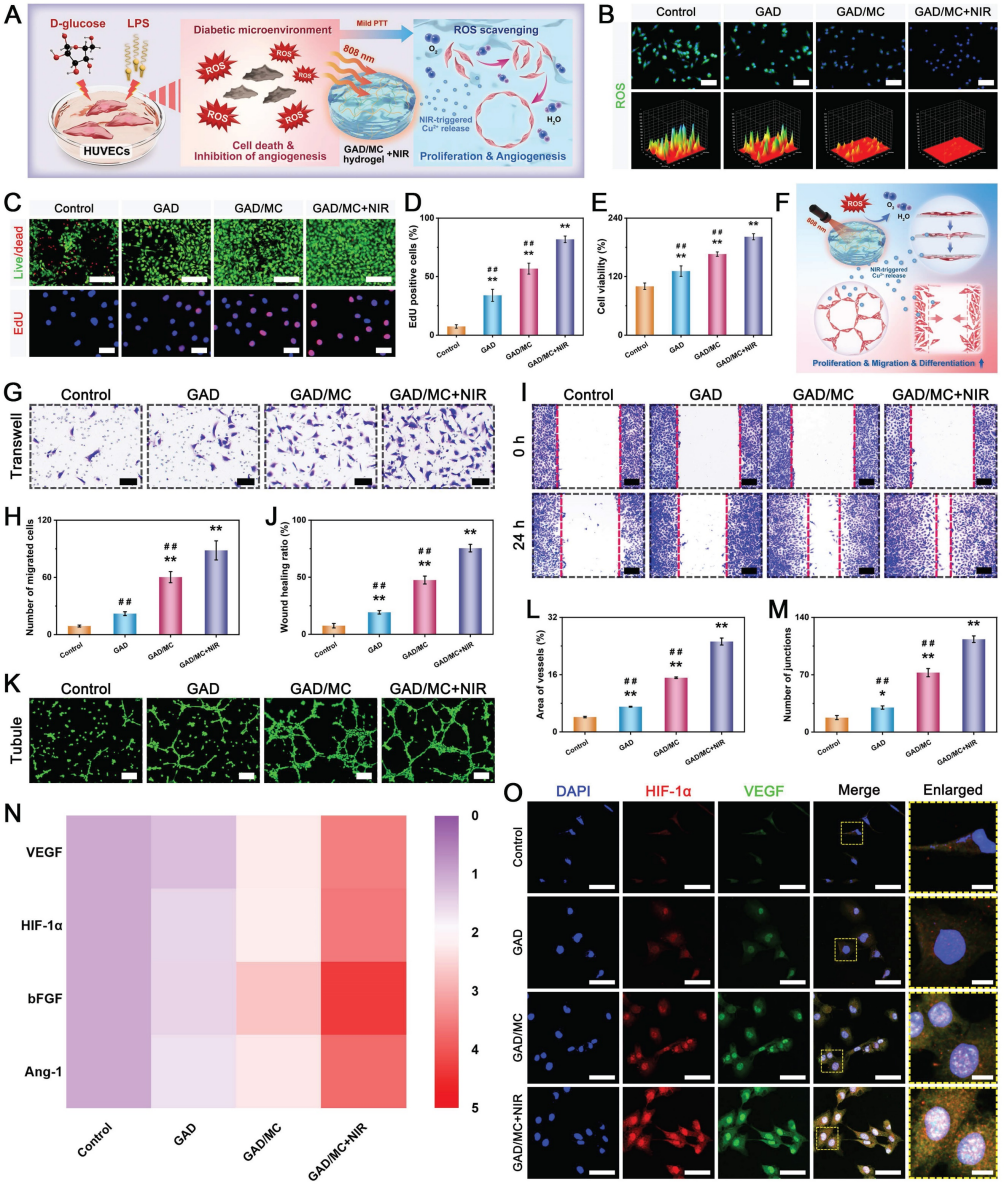
***In vitro* angiogenic activity of the mild photothermal-reinforced hydrogel system. (A)** Schematic illustration of the cytoprotective and proangiogenic effects of the photoactivated GAD/MC hydrogel system on HUVECs under LPS and D-glucose stimulation. **(B)** Intracellular ROS scavenging assay and corresponding 3D surface plot images of HUVECs under LPS and D-glucose stimulation. Scale bar: 50 μm. **(C)** Representative images of live/dead staining and EdU staining of HUVECs under LPS and D-glucose stimulation. Scale bar: 200 μm (live/dead staining images) and 50 μm (EdU staining images). **(D)** Quantitative analysis of the ratio of EdU-positive cells. **(E)** Cell viability of HUVECs after different treatments for 3 days. **(F)** Schematic illustration of the cytoprotective and proangiogenic effects of the photoactivated GAD/MC hydrogel system under LPS and D-glucose stimulation. **(G-H)** Representative Transwell images and quantitative analysis of HUVECs after different treatments for 24 h. Scale bar: 100 μm. **(I-J)** Representative wound healing assay images and quantitative analysis of HUVECs after different treatments for 24 h. Scale bar: 200 μm. **(K-M)** Representative tube formation images and quantitative analysis, including the area of vessels and number of junctions, of HUVECs after different treatments for 8 h. Scale bar: 200 μm. **(N)** Relative mRNA expression of angiogenesis-related markers in HUVECs. **(O)** Representative images of immunofluorescence staining for HIF-1α and VEGF in HUVECs after different treatments. Scale bar: 50 μm (low-magnification images) and 10 μm (high-magnification images). Data are presented as the mean ± SD (n = 3). *P < 0.05 and **P < 0.01 indicate significant differences compared with the control group. ^#^P < 0.05 and ^# #^P < 0.01 indicate significant differences compared with the GAD/MC+NIR group.

Throughout the entire endogenous bone repair process, the reconstruction of a functional vascular network is essential for new bone formation and bone mineralization. Accelerated revascularization is able to provide sufficient nutrients, oxygen, and growth factors needed for cellular function and behavior as well as tissue regeneration, which are key factors in regulating local inflammation and bone healing 31. However, underlying refractory bone defects in diabetic individuals are complex microenvironments in which sustained inflammatory responses, the accumulation of advanced glycation end products, and excessive oxidative stress give rise to microvascular complications and endothelial dysfunction at the local wound/injury site 37. Therefore, it is crucial to maintain a balance in ROS levels and implement strategies to mitigate oxidative stress to restore the biological functions of endothelial cells. Encouraged by the excellent intracellular ROS scavenging performance of the hydrogels in the aforementioned experiments, we established hyperglycemic and inflammatory conditions *in vitro* by adding LPS and D-glucose to mimic the hostile microenvironment of DM and evaluated the antioxidant activity and protective effect of the hydrogel on HUVECs; cells without hydrogel treatment were used as a control **(Figure 7A)**. As illustrated in **Figure 7B**, strong fluorescence signals were clearly detected in the control group, whereas attenuation was noticeably detected in the hydrogel-treated groups, indicating a significant decrease in the intracellular ROS level. In particular, the fluorescence intensity of DCFH-DA dramatically decreased in the GAD/MC- and GAD/MC+NIR-treated groups. The quantitative analysis also revealed that the ROS signals in the GAD/MC+NIR-treated group were lower than those in the other three groups **(Figure S21)**, validating the successful elimination of intracellular ROS by the hydrogel system. The cytoprotective effect of the hydrogel system on HUVECs upon exposure to LPS and D-glucose was then studied. The live/dead staining assay revealed that LPS and D-glucose treatment resulted in massive cell death (red fluorescence), which was primarily due to ROS-induced cellular toxicity **(Figure 7C)**. Compared with the control group, the hydrogel-treated groups presented increased cell viability and proliferation, whereas the GAD/MC+NIR group presented even better performance. Likewise, the results of the EdU staining and CCK-8 assays also revealed that the GAD/MC hydrogel, in conjunction with mild heat stimulation, strongly rescued the HUVECs from oxidative damage-induced cell death as well as elicited a considerable improvement in cellular proliferation **(Figure 7D-E)**, which further verified its strong antioxidant performance. Thus, these results suggest that the GAD/MC hydrogel system, which has excellent antioxidant activity and cytoprotective effects, could scavenge excessive intracellular ROS from an imbalanced immune microenvironment, leading to the significant alleviation of cell damage caused by cellular oxidative stress.

Diabetes-induced endothelial dysfunction is a critical and initiating factor in the pathogenesis of diabetic vascular complications. After confirming the excellent ROS scavenging capacity and cytoprotective effect of the hydrogel system, we investigated the effect of the hydrogel system on endothelial functions under LPS and D-glucose stimulation using a Transwell chamber system **(Figure 7F)**. Endothelial cell migration is a hallmark of angiogenesis and vascular remodeling 72. The results of the Transwell migration and wound healing assays showed that the GAD/MC hydrogel-treated groups exhibited greatly increased migration of HUVECs and significantly enhanced mobility compared with the control group in the presence of LPS and D-glucose **(Figure 7G-J)**, which suggested the potent angiogenic competence of the GAD/MC hydrogel system. Of note, the migration of HUVECs was further enhanced when the GAD/MC hydrogel was combined with thermal stimulation, possibly because the photoactivated hydrogel system can eliminate oxidative damage, and Cu^2+^ ions can promote HUVEC migration and activate the ERK1/2 signaling pathway 73. To confirm the angiogenic potential of the hydrogel system, an *in vitro* tube formation assay was performed under LPS and D-glucose stimulation. The results of the tube formation assay were consistent with those of the Transwell migration assay. As illustrated in **Figure 7K**, a more complete and denser vessel network formed in the GAD/MC+NIR group than in all the other groups. The number of junctions and percentage of vessels was significantly increased in the GAD/MC+NIR-treated group, followed by the GAD/MC and GAD groups, and the least in the control group **(Figure 7L-M)**. To further understand the mechanism regulating angiogenesis, qRT-PCR analysis was carried out to evaluate the expression levels of angiogenesis-related genes in the presence of LPS and D-glucose **(Figure 7N)**. After treatment with the GAD/MC hydrogel for 7 days, the relative expression levels of VEGF, HIF-1ɑ, bFGF, and Ang-1 in HUVECs were significantly increased compared with the other treatment groups. More interestingly, the GAD/MC hydrogel and mild heat stimulation were able to further synergize, elevating the expression of angiogenesis-related genes and resulting in the best stimulatory effect on angiogenesis. Benefiting from the intrinsic angiogenic activity of Cu^2+^ ions, the GAD/MC hydrogel system can stimulate vascular endothelialization by upregulating the expression of VEGF, HIF-1ɑ, bFGF, and Ang-1, which is conducive to accelerated angiogenesis and an adequate blood and oxygen supply under DM conditions. More importantly, MC nanosheets endow hydrogels with excellent photothermal and stimulus-responsive release properties, thus enabling the promotion of blood vessel formation by the combination of intelligent release of the proangiogenic active component (Cu^2+^ ion) and mild thermal stimulation (42 ± 1 °C) under periodic NIR irradiation, thereby facilitating tissue repair and remodeling. High levels of ROS and glucose in diabetic conditions are often associated with decreased levels of HIF-1α, which in turn downregulates the expression of VEGF and thus impairs angiogenesis 29. Therefore, immunofluorescence staining of HIF-1α and VEGF was performed to confirm the qRT-PCR results. Moreover, significantly enhanced expression of HIF-1α and VEGF was found in the GAD/MC and GAD/MC+NIR groups, whereas the expression of these two markers of neovascularization was decreased in the control group. Notably, under periodic NIR irradiation, the GAD/MC-treated (GAD/MC+NIR) group exhibited an even more pronounced effect **(Figure 7O)**, which can be attributed to the dual effects of mild thermal stimulation and Cu^2+^ ions. Numerous studies have demonstrated that both Cu ions and mild thermal effects can effectively activate HUVECs, resulting in enhanced proliferation, migration, and angiogenesis via the targeting of various proangiogenic molecules, including VEGF, HIF-1ɑ, bFGF, Ang-1, and eNOS 56. It is worth emphasizing that adequate ROS scavenging can synergistically promote angiogenesis, while the formation of a robust vascular network can in turn mitigate oxidative stress and promote tissue regeneration. Accordingly, the catechol-Cu coordination structure within the hydrogel also plays an important role in restoring the function of impaired HUVECs induced by diabetes, thereby contributing to improved cellular activity and enhanced angiogenesis. Taken together, the remarkable proangiogenic capacity of the GAD/MC hydrogel system can be ascribed to the combined vascular regulatory activity of the mild heat effect and Cu, as well as the ability of the hydrogel system to inhibit oxidative stress and promote cell attachment and migration, suggesting great potential in DM-related tissue regeneration *in vivo*.

### Macrophage-mediated osteoblast/osteoclast differentiation and endothelial cell vascularization

Bone regeneration initiates an acute inflammatory response accompanied by a well-orchestrated series of biological events, such as the immune response, osteogenesis, angiogenesis, osteoclastogenesis, and biomineralization. Each of these processes entails intricate interactions with diverse cell types (e.g., osteoblasts, endothelial cells, osteoclasts, and different types of immune cells) 74. However, in cases of uncontrolled inflammatory dysregulation, particularly in patients with diabetes, the bone healing process is inevitably impeded due to an imbalance of osteoblasts/osteoclasts and impaired neovascularization 3. This is because, under diabetic inflammatory conditions, dysfunctional macrophages are more inclined to transform into the proinflammatory M1 phenotype, accompanied by the overproduction of proinflammatory factors and ROS to trigger excessive inflammation and osteoclastogenesis, ultimately leading to attenuated osteo/angiogenesis and bone formation. It has been well documented that macrophages not only modulate the local inflammatory microenvironment but also produce a variety of beneficial factors to mediate the interactions among angiogenesis, osteogenesis, and osteoclastogenesis, revealing their pivotal involvement in both bone formation and remodeling processes 35.

**Figure 8:**
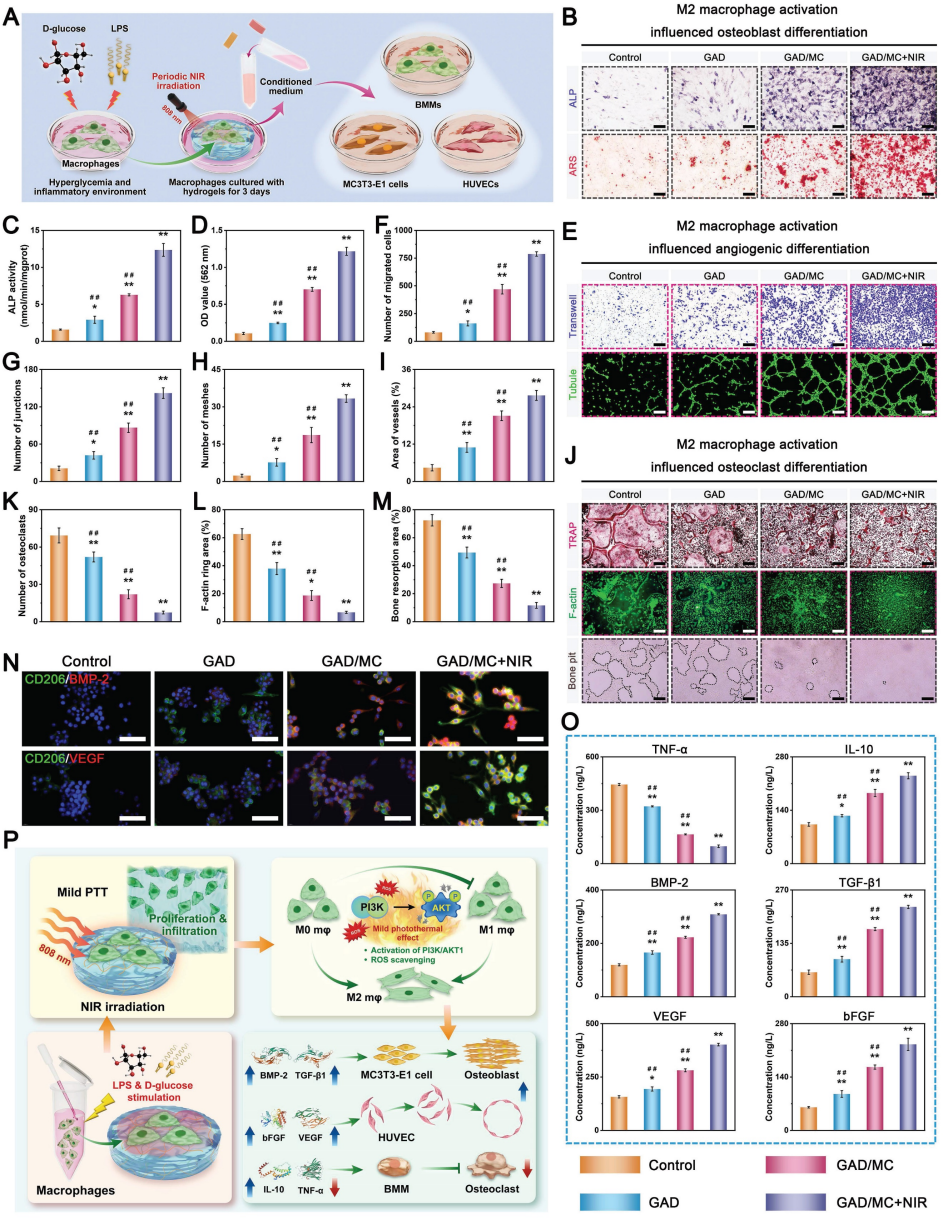
***In vitro* osteoimmunomodulatory performance of the mild photothermal-reinforced hydrogel system. (A)** Schematic illustration of conditioned medium preparation and the cell culture process. **(B)** Representative images of ALP and ARS staining of MC3T3-E1 cells after different treatments. Scale bar: 200 μm**. (C-D)** Quantitative analysis of ALP activity and ECM mineralization. **(E)** Representative images of Transwell migration and tube formation assays of HUVECs after different treatments. Scale bar: 200 μm. **(F-I)** Quantitative analysis of Transwell migration and tube formation assays. **(J)** Representative images of TRAP, F-actin ring, and bone pit assays of BMMs after different treatments. Scale bar: 200 μm. **(K-M)** Quantitative analysis of TRAP staining, F-actin ring, and bone resorption pit assays. The black dotted lines indicate the bone resorption areas caused by activated osteoclasts. Scale bar: 100 μm. **(N)** Representative images of immunofluorescence staining for BMP-2, VEGF, and CD206 in RAW264.7 cells after different treatments. Scale bar: 50 μm.** (O)** ELISA analyses of inflammatory and pro-healing factors secreted by macrophages after different treatments. **(P)** Schematic illustration of the osteoimmunomodulatory effect of the photoactivated GAD/MC hydrogel system on angiogenesis, osteogenesis, and osteoclastogenesis. Data are presented as the mean ± SD (n = 3). *P < 0.05 and **P < 0.01 indicate significant differences compared with the control group. ^#^P < 0.05 and ^# #^P < 0.01 indicate significant differences compared with the GAD/MC+NIR group.

Moreover, as mentioned earlier, we confirmed that the hydrogel system has significant potential not only for protecting macrophages against oxidative stress injury by scavenging ROS but also for stimulating the conversion of proinflammatory M1 macrophages to anti-inflammatory and pro-regenerative M2 macrophages under diabetic inflammatory conditions, which may trigger a beneficial immune response that may serve as a chemical cue for regulating cellular function. However, whether this beneficial immunomodulatory effect is capable of modulating the immune microenvironment and facilitating osteogenic differentiation and angiogenesis while inhibiting osteoclast differentiation remains to be explored. To further verify the osteoimmunomodulatory role and underlying mechanism of the hydrogel in osteoblast/osteoclast differentiation and angiogenesis, we cocultured osteoblasts, endothelial cells, and osteoclasts with conditioned medium from polarized macrophages **(Figure 8A)**. In terms of osteogenic activity, the GAD/MC+NIR group substantially reversed the inhibitory effect of LPS and D-glucose by increasing ALP expression and calcium mineral deposition, which was further confirmed by quantitative analysis **(Figure 8B-D)**. It has been suggested that M2 macrophages are capable of promoting the differentiation and mineralization of osteoblasts by secreting BMP-2 and TGF-β1 75. Similarly, compared to the control group, the hydrogel-treated groups also showed excellent promotion of angiogenesis, as evidenced by enhanced cell migration and tube formation **(Figure 8E-I)**. Furthermore, in contrast with the incomplete or sparsely distributed tubular networks in the control group, those in the GAD/MC and GAD/MC+NIR groups markedly enhanced the vascularization ability of HUVECs, as shown by the increased number of migrated cells, junctions, and meshes. It has been recently demonstrated that the anti-inflammatory and angiogenic cytokines secreted by M2 macrophages can stimulate angiogenesis and thus bridge initial inflammation and subsequent tissue regeneration and repair 76. Moreover, another potential reason for the enhanced vascularization could be attributed to the presence of bioactive Cu^2+^. Accordingly, photoactivated hydrogel system-induced M2 macrophages are conducive to osteogenic differentiation and vascularization through the secretion of growth factors and chemokines, as reported in the literature 77. These findings indicated that the synergistic enhancement effect of immunomodulation and Cu ions successfully achieved high-efficiency proangiogenic activity, which is beneficial for augmented bone regeneration.

Having confirmed the functional improvements in osteoblasts and endothelial cells by macrophage-mediated osteoimmunomodulation, we next explored the crosstalk between macrophages and osteoclasts. Accumulating evidence has demonstrated that the occurrence of bone loss and impaired bone healing in individuals with DM may be intricately linked to oxidative stress, immune dysfunction, and abnormal activation of osteoclasts 78. In the case of excessive inflammation under diabetic conditions, M1 macrophages tend to secrete or produce large amounts of proinflammatory factors and ROS, contributing to enhanced osteoclast differentiation and inhibited osteoblast differentiation. IL-6, in particular, is one of the most important proinflammatory cytokines secreted by M1 macrophages and can impede the bone healing process by promoting osteoclast differentiation and bone resorption 4. Besides, cytokines such as IL-1β and TNF-α disrupt homeostasis between bone formation by osteoblasts and bone resorption by osteoclasts through the activation of the proinflammatory transcription factor nuclear factor-kappa B (NF-κb) 79. To investigate whether the hydrogel system inhibits osteoclastogenesis through orchestrating M2 macrophage polarization, different CMs were prepared for subsequent experiments, including TRAP staining, F-actin ring immunofluorescence staining, and bone resorption tests **(Figure 8J)**. Our data showed that, compared with the control group, the hydrogel-treated groups exhibited strongly inhibited osteoclast differentiation and maturation of osteoclast progenitor cells from mouse bone marrow and more profound anti-osteoclast activity in the GAD/MC group, as evidenced by the reductions in TRAP^+^ multinucleated osteoclasts, F-actin ring formation, and bone resorption areas **(Figure 8K-M)**. Importantly, this biological effect was dramatically augmented after treatment with mild thermal stimulation induced by daily periodic NIR irradiation. In addition to modulating the inflammatory environment, macrophages can mediate bone regeneration and revascularization by secreting functional factors. For example, BMP-2 and VEGF are potent inducers of osteogenesis and angiogenesis produced by M2 macrophages 80. The expression levels of BMP-2 and VEGF in macrophages cocultured with the hydrogels were detected by immunofluorescence staining and ELISA. As shown in **Figure 8N**, more endogenous BMP-2 and VEGF colocalized with CD206^+^ macrophages appeared in the GAD/MC+NIR group than in the GAD/MC and GAD groups, providing further evidence that macrophages stimulated by the GAD/MC hydrogel secreted more beneficial cytokines, which in turn promoted osteogenesis and angiogenesis through providing an instructive osteoimmune microenvironment. Additionally, we also found that the expression levels of BMP-2 and VEGF in macrophages after various treatments were similar to those of M2 macrophage-related markers (IL-4 and IL-10). The ELISA results are shown in **Figure 8O** and are consistent with the results mentioned above; macrophages treated with the GAD/MC hydrogel under periodic NIR irradiation secreted lower amounts of the proinflammatory cytokine TNF-α but more of the anti-inflammatory cytokine IL-10. Concurrently, the levels of osteogenic and angiogenic cytokines, including BMP-2, TGF-β1, VEGF, and bFGF, were significantly elevated in both the GAD/MC and GAD/MC+NIR groups, especially in the latter group, suggesting the potential of the hydrogel system to provide an anti-inflammatory and pro-healing microenvironment for expedited tissue healing. Numerous studies have shown that the immune microenvironment plays a critical regulatory role in bone homeostasis and bone diseases 81. As typical intrinsic immune cells, macrophages exhibit significant heterogeneity and actively coordinate revascularization, osteogenic differentiation, biomineralization, and osteoclast differentiation through the production of a variety of inflammatory and growth factors, which in turn profoundly affect the endogenous repair process 81. Researchers have shown that the cytokines IL-6 and TNF-α produced by M1 macrophages can enhance osteoclast differentiation and inhibit osteoblast differentiation, leading to low-bone mass phenotypes, whereas the cytokines IL-4 and IL-10 produced by M2 macrophages can inhibit osteoclastogenesis by inhibiting the nuclear factor kappa-B (NF-κB) signaling pathway 69, 82. In addition, animal experiments have shown that the local hyperglycemic and inflammatory environment of diabetic bone defects trigger M1-like macrophage infiltration at the injured site, leading to the sustained release of proinflammatory cytokines, hindering the transition from the inflammatory phase to the repair phase, and consequently delaying bone healing 26. More intriguingly, several recent works have demonstrated that periodic NIR irradiation-assisted mild PTT could promote RAW264.7 polarization toward the M2 type and the release of anti-inflammatory factors (IL-4, IL-10, and Arg1) by activating the PI3K/AKT1 signaling pathway while inhibiting polarization toward the M1 type and proinflammatory factors 16. The PI3K/AKT1 signaling pathway is considered an important signaling pathway for macrophage polarization to the M2 phenotype. In comparison, the NF-κB signaling pathway is the key to activating M1-type macrophages 83.

Furthermore, the activation of the PI3K/AKT1 signaling pathway in macrophages may provide an instructive early immune microenvironment that promotes tissue regeneration by secreting functional cytokines, accelerating angiogenesis, and maintaining the osteoblast/osteoclast balance 68. In the present study, the protein expression of PI3K and p-AKT was significantly upregulated in the GAD/MC+NIR group, whereas no difference was observed between the GAD/MC and control groups (**Figure S22**). These findings strongly suggest that mild photothermal treatment plays a regulatory role in reducing the inflammatory response and maintaining M2 macrophage polarization through the PI3K/AKT signaling pathway, corroborating our previous findings 30. Hence, according to the above results, photoactivated GAD/MC hydrogel system-induced M2 macrophages were conducive to osteo/angiogenesis promotion and osteoclastogenesis inhibition by the secretion of growth factors and chemokines, as reported in the literature 84. The mechanism by which the hybrid GAD/MC hydrogel guided macrophage infiltration, proliferation, and polarization and subsequently influenced osteo/angiogenesis and osteoclastogenesis under DM conditions is illustrated in **Figure 8P**. As such, through the secretion of beneficial cytokines and growth factors such as IL-10, BMP-2, and VEGF, these M2-polarized macrophages establish an instructive immune microenvironment that protects osteoblasts and endothelial cells from oxidative stress and a disordered immune microenvironment and suppresses osteoclast activity and bone resorption (osteoimmunomodulation), which is expected to accelerate bone regeneration under DM conditions. Collectively, these findings demonstrated that the hydrogel system not only possessed good antioxidant and anti-inflammatory activities, which effectively mitigated inflammation via the elimination of intracellular ROS and activation of M2 macrophages but also modulated the secretion of inflammatory cytokines and growth factors to create a microenvironment conducive to the promotion of osteogenesis/angiogenesis and the inhibition of osteoclastogenesis.

### *In vivo* biocompatibility and bioactivity

**Figure 9:**
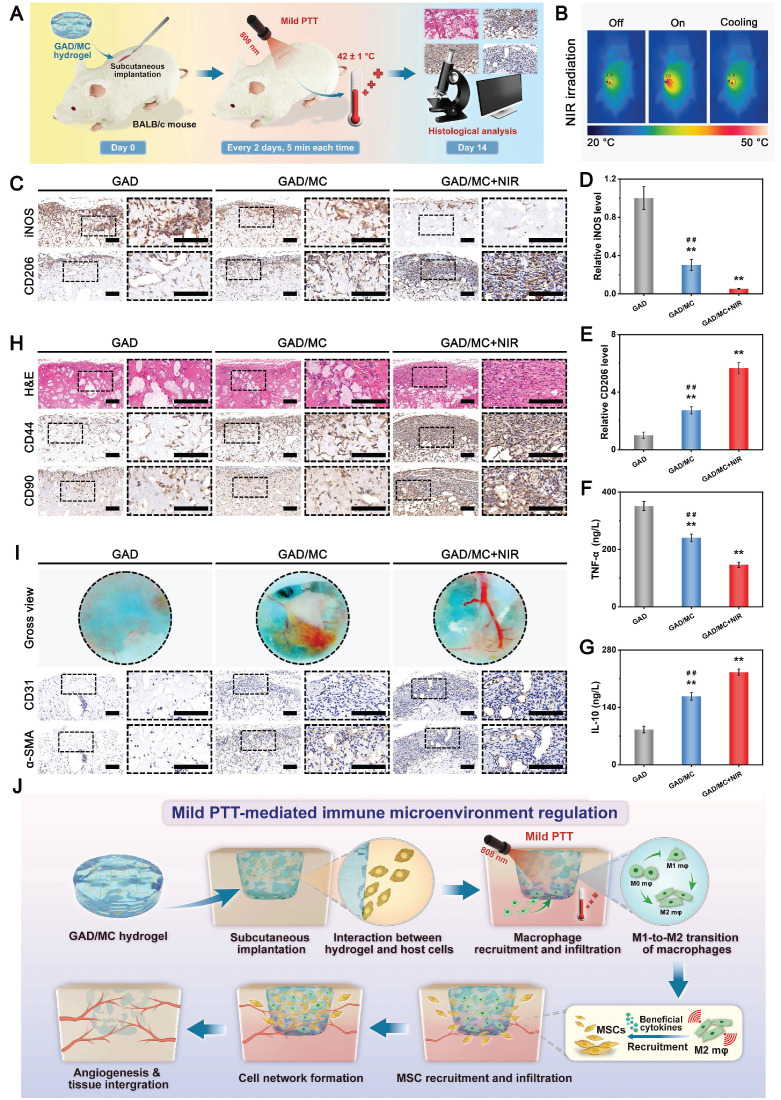
***In vivo* biological performance of the mild photothermal-reinforced hydrogel system in a mouse subcutaneous implantation model. (A)** Schematic illustration of the establishment of a subcutaneous implantation model in BALB/c mice and the experimental design used to evaluate the effects of the hydrogels on immune regulation. **(B)** Infrared thermal images of the implantation site in mice under NIR irradiation (808 nm, 1.5 W/cm^2^). **(C-E)** Representative immunohistochemical staining images and quantitative analysis of iNOS and CD206 after implantation for 2 weeks. Scale bar: 100 μm. **(F-G)** Quantitative analysis of inflammatory cytokines (TNF-α and IL-10) *in vivo* via ELISA. **(H)** Representative images of H&E staining and immunohistochemical imaging (CD44 and CD90) after implantation for 2 weeks. Scale bar: 100 μm. **(I)** Photographs of the hydrogel and surrounding tissue and representative images of immunohistochemical staining for CD31 and α-SMA after implantation for 2 weeks. Scale bar: 100 μm. **(J)** Schematic illustration of the mechanism of the GAD/MC hydrogel system in immunomodulation and subsequent tissue integration. Data are presented as the mean ± SD (n = 3). *P < 0.05 and **P < 0.01 indicate significant differences compared with the GAD group. ^#^P < 0.05 and ^# #^P < 0.01 indicate significant differences compared with the GAD/MC+NIR group.

On the basis of a series of *in vitro* experiments mentioned above, the GAD/MC hydrogel system exhibited evident advantages in modulating the immune microenvironment and promoting osteoblast and vascular endothelial cell migration and differentiation. To further validate the synergistic therapeutic effect of the GAD/MC hydrogel plus mild photothermal stimulation on accelerating endogenous tissue regeneration, a subcutaneous implantation model of BALB/c mice was established, followed by treatment with periodic NIR irradiation (808 nm, 1.5 W/cm^2^) for 5 min every 2 days. The whole process of the *in vivo* experiment is shown in **Figure 9A**. During the irradiation period, the local temperature reached a mild hyperthermal temperature range of 42 ± 1 °C **(Figure 9B)**, which has been shown to be favorable for tissue regeneration 85. This promising result indicated that the prepared hydrogel system still retained its photothermal effect, which was feasible for providing long-term thermal cues for osteogenesis *in vivo*.

A disordered immune microenvironment involves chronic inflammation with excessive M1 macrophages. Prolonged and excessive M1 macrophage accumulation leads to chronic inflammation, fibrous encapsulation, and delayed tissue healing. In contrast, M2 macrophages can recreate a favorable anti-inflammatory and pro-regenerative niche by secreting anti-inflammatory and tissue repair-related factors 86. Hence, we next investigated whether the hydrogel system could improve the immune microenvironment at the early stage of tissue regeneration. After 2 weeks of implantation, more CD206^+^ macrophages were observed in the GAD/MC+NIR group than in the GAD/MC and GAD groups **(Figure 9C)**, confirming the adequate anti-inflammatory effect *in situ*. Quantitative analysis further confirmed that upon treatment with the GAD/MC hydrogel plus NIR irradiation, the proportion of M2 (CD206^+^) macrophages markedly increased, whereas the proportion of M1 (iNOS^+^) macrophages decreased **(Figure 9D-E)**, accompanied by reduced secretion of the proinflammatory cytokine TNF-α **(Figure 9F)**. Hence, the synergistic utilization of periodic NIR irradiation neither damaged the surrounding normal tissues nor caused an obvious inflammatory response. Simultaneously, the ELISA results further confirmed the remarkable bioactivity of the hydrogel system on macrophage polarization and anti-inflammatory effects. Compared with that in the GAD group, the secretion of the anti-inflammatory factor IL-10 was dramatically elevated in the GAD/MC and GAD/MC+NIR groups, with the GAD/MC+NIR group exhibiting the most pronounced effects **(Figure 9G)**, indicating that the GAD/MC hydrogel system effectively mitigated the inflammatory response under periodic NIR irradiation. These findings suggested that the prepared hydrogel system could increase the levels of anti-inflammatory factors to inhibit inflammation and further promote M2 macrophage polarization, ensuring the course of regeneration. As previously described, IL-10 is one of the most important anti-inflammatory cytokines secreted by M2 macrophages 76. The coordination between the catechol groups and Cu^2+^ as well as mild PTT induced by on-demand NIR irradiation resulted in excellent synergistic anti-inflammatory and immunomodulatory properties owing to their powerful ROS-scavenging effects and enhanced M2 macrophage activation. These results were consistent with those of the *in vitro* experiments, indicating that enhanced M2 macrophage polarization and anti-inflammatory cytokine secretion were induced in the GAD/MC and GAD/MC+NIR groups, especially in the latter group, which would create a conducive microenvironment for subsequent cell function and tissue regeneration. Additionally, H&E staining revealed visible host cell infiltration around and inside the hydrogels without the formation of fibrous capsules **(Figure 9H)**, indicating that these hydrogels had good cellular compatibility and could be gradually degraded to provide the necessary space for cell infiltration. Unexpectedly, remarkable cellular networks with high cell density formed throughout the entire interior of the GAD/MC hydrogel, especially after being subjected to periodic NIR irradiation, while only a loose cellular network with much lower cell density was observed in the GAD hydrogel. These results suggested that the GAD/MC hydrogel facilitated integration with the surrounding tissue by promoting the migration and recruitment of endogenous host cells with the aid of mild thermal stimulation. The 3D ECM-mimicking porous structures allow extensive cell infiltration and nucleated cells rapidly penetrate throughout the hydrogel matrix after implantation 71. In addition, the good integration of the GAD/MC hydrogel system could be attributed to DA-mediated cell adhesion as well as improved blood circulation induced by mild heat. Consistent with the results of the *in vitro* anti-inflammation and biocompatibility, the GAD/MC-based therapeutic strategy was highly effective at ameliorating inflammation during the early stages of *in vivo* implantation, which was conducive to endogenous cell infiltration and tissue regeneration.

The ability of cell-free bone repair scaffolds to promote the recruitment and infiltration of endogenous stem cells is a prerequisite for bone regeneration. Immunohistochemical staining revealed that the expression of CD44 and CD90, two typical markers of mesenchymal stem cells, in the GAD/MC group was higher than that in the GAD group **(Figure 9H)**, indicating enhanced recruitment of tissue-repairing progenitor cells, which might be favorable for osteogenesis and subsequent bone mineralization. Furthermore, we found that this promoting effect became more evident after mild NIR treatment, as evidenced by the highest positive expression of CD44 and CD90 in the GAD/MC+NIR group **(Figure S23)**. This finding indicated that the favorable immune microenvironment induced by the GAD/MC hydrogel with NIR irradiation effectively guided the migration of endogenous stem cells to the hydrogel, revealing its great potential for promoting osteogenesis and tissue remodeling. Consequently, the photoactivated GAD/MC hydrogel system displayed good biocompatibility and integrated well with the surrounding tissue, allowing for cell recruitment and immunomodulation, which was beneficial for cell-material interactions and *in situ* osteointegration during bone repair. In addition, timely and adequate angiogenesis after bone scaffold implantation plays a fundamental role in bone regeneration 87. At 2 weeks, according to the macroscopic analysis, it was observed that the distribution of capillary vessels around and within the GAD group was limited, while the proangiogenic activities of the GAD/MC and GAD/MC+NIR groups were stronger, especially those of the latter group **(Figure 9I)**, displaying a much greater potential for inducing angiogenesis. The abundant vessels within the GAD/MC+NIR group are believed to provide sufficient nutrients and oxygen to effectively enhance bone regeneration. The results of the immunohistochemical analysis agreed with the macroscopic observations **(Figure 9I)**. The GAD/MC+NIR group exhibited more mature vascular networks and a significantly increasing density of microvessels stained by CD31 and ɑ-SMA, while only some individual CD31^+^ and α-SMA^+^ host vascular endothelial cells were observed within the GAD/MC group, and no CD31^+^ cells were observed within the GAD group **(Figure S23)**. These findings further verified the cooperative proangiogenic effect of mild thermal stimulation and Cu^2+^ ions, which were beneficial for cell infiltration and vascular ingrowth throughout the entire interior of the hydrogel **(Figure 9J)**.

Numerous studies have also demonstrated that the synergistic utilization of bioactive Cu ions and mild thermal stimulation has significant efficacy in activating vascular endothelial cells, resulting in enhanced proliferation, migration, and angiogenesis 56. Furthermore, the formation of new blood vessels in the early stage of tissue repair facilitates improved oxygen and nutrient transport, thus reducing the production of free radicals, facilitating growth factor secretion, and recruiting endogenous stem cells to reconstruct the injured tissue 73. All these results support the superiority of the photoactivated GAD/MC hybrid hydrogel system in alleviating local inflammation, boosting endogenous MSC infiltration and recruitment, and promoting angiogenesis, which is conducive to subsequent tissue regeneration and remodeling.

### *In vivo* bone repair with or without comorbidities

**Figure 10:**
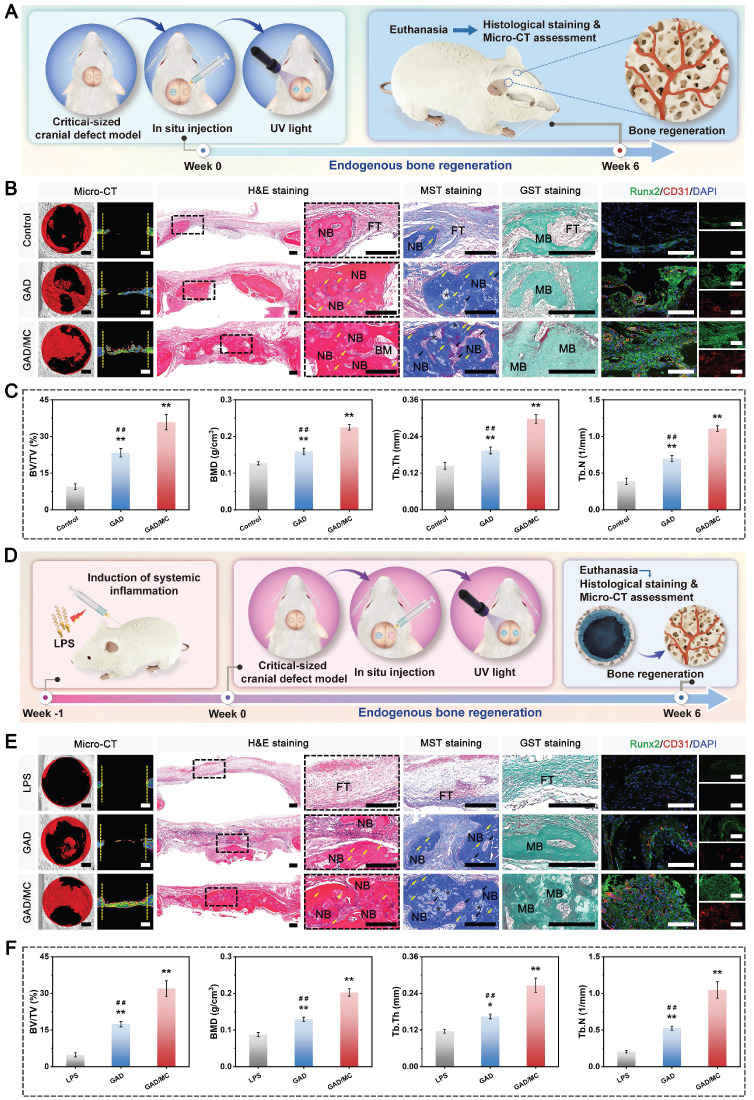
***In vivo* bone regeneration under acute trauma and inflammatory conditions. (A)** Schematic illustration of the establishment of a critical-sized calvarial defect model in SD rats and the experimental design used to evaluate the effects of the hydrogels on bone repair. **(B)** Representative images of micro-CT scanning, H&E staining, MST staining, GST staining, and immunofluorescence staining of Runx2 and CD31 in different treatment groups. FT: fibrous tissue. NB: newly formed bone tissue. BM: bone marrow. MB: mineralized bone tissue. The yellow arrows represent the bone lacunae. The black arrows represent the central canal. The black asterisks represent the residual hydrogel materials. Scale bar: 1 mm (micro-CT images), 200 μm (H&E staining, MST staining, and GST staining images), and 100 μm (immunofluorescence staining images). **(C)** Quantitative analysis of BV/TV, BMD, Tb.Th, and Tb.N in the different treatment groups. **(D)** Schematic illustration of the establishment of a critical-sized calvarial defect model in LPS-treated rats and the experimental design used to evaluate the effects of the hydrogels on bone repair. **(E)** Representative images of micro-CT scanning, H&E staining, MST staining, GST staining, and immunofluorescence staining of Runx2 and CD31 in different treatment groups. FT: fibrous tissue. NB: newly formed bone tissue. BM: bone marrow. MB: mineralized bone tissue. The yellow arrows represent the bone lacunae. The black arrows represent the central canal. The black asterisks represent the residual hydrogel materials. Scale bar: 1 mm (micro-CT images), 200 μm (H&E staining, MST staining, and GST staining images), and 100 μm (immunofluorescence staining images). **(F)** Quantitative analysis of BV/TV, BMD, Tb.Th, and Tb.N in different treatment groups. Data are presented as the mean ± SD (n = 3). *P < 0.05 and **P < 0.01 indicate significant differences compared with the control or LPS group. ^#^P < 0.05 and ^# #^P < 0.01 indicate significant differences compared with the GAD/MC group.

On the basis of these great performances both *in vitro* and *in vivo*, we established a critical-sized calvarial defect model (Φ = 5 mm) in SD rats to mimic the post-traumatic conditions of bone injury and evaluate the therapeutic effect of the GAD/MC hydrogel on endogenous bone regeneration. As shown in **Figure 10A**, the prepared hydrogel precursor could be conveniently injected into the circular bone defects, followed by *in situ* gelation induced by UV light irradiation. After 6 weeks of implantation, the collected cranial samples were processed for micro-CT scanning and subsequent histological analysis. As shown in **Figure 10B**, the micro-CT data revealed that more mineralized bone matrix formed in the GAD/MC group than in the other groups, which led to the apparent bridging of the defect within 6 weeks, suggesting its superior ability to promote bone repair. Bone regeneration capacity was further evaluated through quantitative analyses of morphological parameters, including bone volume/total volume (BV/TV), bone mineral density (BMD), trabecular thickness (Tb.Th), and trabecular number (Tb.N). After 6 weeks, the BV/TV of the GAD/MC group was 3.8-fold and 1.5-fold higher than that of the GAD and control groups, respectively (**Figure 10C**). Tb.Th and Tb.N also showed similar trends, with the best bone regeneration outcome detected in the GAD/MC group, indicating that the hydrogel system accelerated bone healing. In addition, the H&E, Masson's trichrome (MST), and Goldner's trichrome (GST) staining results within the bone defect areas were consistent with the micro-CT data. That is, much more newly formed neo-bone tissue and mineralized collagen deposition appeared in the GAD/MC group than in the other two groups **(Figure 10B)**, indicating excellent performance in inducing new bone formation and biomineralization. Furthermore, at 6 weeks post-implantation, the GAD/MC group displayed the highest expression of the osteogenic marker Runx2 and the angiogenic marker CD31, while minimal Runx2 and CD31 expression was observed in the control group **(Figure 10B)**. Collectively, these results confirmed the significant potential of the injectable GAD/MC hydrogel system for accelerating endogenous bone regeneration *in vivo*.

To further assess whether the GAD/MC hydrogel can facilitate endogenous bone regeneration under comorbidity conditions, we constructed a critical-sized cranium defect model in rats that were pretreated with LPS to induce systemic inflammation. LPS, an endotoxin obtained from bacteria, plays a pivotal role in triggering inflammation progression 88. Schematic diagrams of the construction and treatment of bone defects under LPS-induced inflammatory conditions are presented in **Figure 10D**. In contrast to that in the control group, an intraperitoneal injection of LPS elicited a systemic inflammatory microenvironment, as evidenced by the significantly higher average white blood cell (WBC) count in the LPS group **(Figure S24A)**. In addition, immunofluorescence staining of proinflammatory markers (IL-6 and TNF-α) at 6 weeks post-implantation showed that the expression levels of these inflammatory factors in the LPS group were higher than those in the control group without LPS treatment **(Figure S24B)**, further validating the successful establishment of the inflammation model. Subsequently, the *in vivo* osteoinductive activity of the hydrogels under inflammatory conditions was analyzed by micro-CT scanning. As illustrated in **Figure 10E**, the 3D reconstruction and coronal plane images of the defect area showed less new bone formation in the LPS group than in the control group due to the limited self-repairing capacity of the bone tissue in the persistent inflammatory microenvironment. This finding was identical to those of previous reports on bone osteogenesis repression caused by processive and uncontrolled inflammation 1, 89. Comparatively, both the GAD and GAD/MC groups, especially the latter, displayed a significant amount of regenerated new bone formation in the defect area, as evidenced by the elevated BV/TV, BMD, Tb.Th, and Tb.N values **(Figure 10F)**, which suggested that the injectable GAD/MC hydrogel system had superior osteoinductive activity under LPS-induced inflammatory conditions. The enhanced efficacy of the GAD/MC hydrogel in the healing of inflammation-related bone defects was also confirmed through histological staining, where substantial osteocytes/osteoblasts infiltrated into the defect areas, accompanied by the formation of high-density mineralized tissue **(Figure 10E)**. In contrast, only a small amount of newly formed collagen matrix was observed in the defect area of the LPS group, indicating delayed bone healing. Notably, the regenerated bone tissues of the GAD/MC group under comorbidity conditions achieved the same repair effect as those of the GAD/MC group under normal conditions. As a chronic tissue trauma characterized by abnormal osteo/angiogenesis and a severe inflammatory microenvironment, the effective healing of LPS-stimulated critical-sized cranial defects generally requires excellent anti-inflammation and osteo/angiogenesis promotion 10. Through immunofluorescence staining of Runx2 and CD31, it was observed that the GAD/MC group possessed a strong capability to promote osteogenesis and induce neovascularization **(Figure 10E)**, thereby contributing to the efficient healing of critical-sized bone defects in the inflammatory microenvironment. To further assess the inflammatory reaction induced by the GAD/MC hydrogel, immunohistochemical staining of TNF-α and IL10 was performed **(Figure S25)**. Due to severe inflammation, the LPS-treated group showed high expression of proinflammatory TNF-α, while the hydrogel-treated groups exhibited effectively reversed inflammatory expression. In particular, inflammatory responses were markedly reduced in the GAD/MC group, and immune homeostasis was restored by downregulating the expression of the proinflammatory factor TNF-α and upregulating the expression of the anti-inflammatory factor IL-10, suggesting potent anti-inflammatory activity. Consequently, these results showed that the GAD/MC hydrogel system can efficiently induce osteogenesis and promote bone defect healing in cases of excessive inflammation, demonstrating its potential to treat bone defects in complex situations such as diabetes or infections.

**Figure 11:**
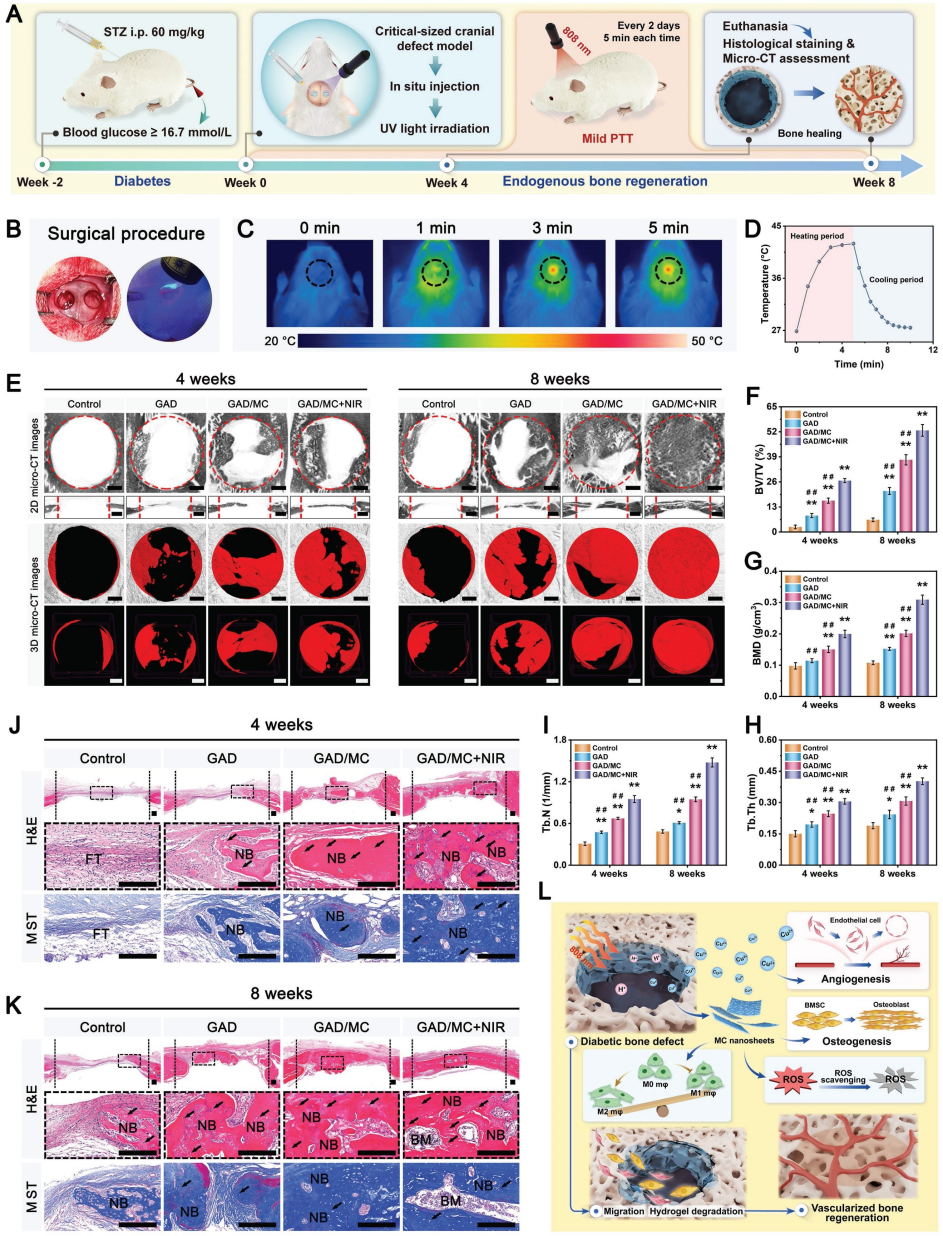
***In vivo* therapeutic effect of the mild photothermal-reinforced hydrogel system on critical-sized cranial defects in diabetic rats. (A)** Schematic illustration of the establishment and treatment of the critical-sized cranial defect model in diabetic rats.** (B)** Surgical construction and hydrogel injection of the critical-sized cranial defect model. **(C)** Real-time NIR thermal images and **(D)** temperature changes in the defect area after local injection of the GAD/MC hydrogel under NIR laser irradiation (1.5 W/cm^2^, 808 nm) during *in vivo* treatment. **(E)** Representative 3D reconstructed micro-CT images of calvarial defects after different treatments at 4 and 8 weeks. Scale bar: 1 mm. Quantitative analysis of bone regeneration-related parameters, including **(F)** BV/TV, **(G)** BMD, **(H)** Tb.Th, and **(I)** Tb.N, from the micro-CT images. **(J-K)** Representative H&E and MST staining images of the decalcified defect region at 4 and 8 weeks. FT: fibrous tissue. NB: newly formed bone tissue. BM: bone marrow. The black arrows represent the bone lacunae. Scale bar: 200 μm. **(L)** Schematic diagram showing the potential therapeutic mechanism of bone healing by the mild photothermal-reinforced hydrogel system. Data are presented as the mean ± SD (n = 3). *P < 0.05 and **P < 0.01 indicate significant differences compared with the control group. ^#^P < 0.05 and ^# #^P < 0.01 indicate significant differences compared with the GAD/MC+NIR group.

The hydrogel system promoted the acceleration of bone defect repair and reconstruction under both acute trauma and persistent inflammatory conditions. However, given the complexity and variability of the pathological microenvironment in diabetes, immune regulation and bone regeneration *in vivo* need further research. Accordingly, to evaluate the therapeutic potential and possible mechanisms of the engineered hydrogel system in modulating the immune microenvironment and promoting bone healing, we used a streptozotocin (STZ)-induced cranial defect model in diabetic SD rats, as schematically illustrated in** Figure 11A**. Then, different groups of hydrogels could readily fill the circular defect cavity via minimally invasive injection and achieve rapid gelation *in situ* upon UV irradiation **(Figure 11B)**, with the blank group serving as the control. During the entire experimental process, blood glucose levels and bone formation were monitored, and the results showed that blood glucose levels remained within 18-30 mmol/L **(Figure S26)**. After 3 days of hydrogel implantation, animals in the GAD/MC+NIR group received mild NIR irradiation (808 nm, 1.5 W/cm^2^) for 5 min every 2 days, and the temperature at the defect site was maintained at 42 ± 1 °C **(Figure 11C-D)**. The temperature variation trend was recorded using an infrared thermal imaging camera. After 4 and 8 weeks of treatment, rat cranial samples were obtained and scanned using micro-CT, and bone tissue reconstruction was performed. Compared with those in the GAD/MC group, the 3D micro-CT reconstructed images of the defect area revealed enhanced new bone formation around both the defect edges and the center in the GAD/MC group at both time points, which became more prominent with the aid of mild heat stimulation **(Figure 11E)**. The best bone regeneration performance observed in the GAD/MC+NIR group could be attributed to MC incorporation and NIR-assisted mild hyperthermia, which jointly promoted the proliferation and osteogenic differentiation of osteoblasts. The examination of 2D sectional images further validated the result, illustrating that a complete bone bridge connecting the defects appeared in the GAD/MC+NIR group. Conversely, minimal bone matrix formation and even bone mass reduction were observed at the edges of the bone cavity in the untreated blank control group, indicating that the presence of diabetic conditions seriously impeded new bone formation. Notably, the amount of newly regenerated bone tissue along the boundaries of the bone defect significantly increased after 4 weeks and approximately occupied all the residual defect regions after 8 weeks in the GAD/MC+NIR group.

Quantitative analysis based on micro-CT data also verified the increase in new bone formation by the photoactivated GAD/MC hydrogel system (GAD/MC+NIR group), as indicated by elevated BV/TV, BMD, Tb.Th, and Tb.N values, followed by the GAD/MC group and GAD group at both weeks 4 and 8 **(Figure 11F-I)**. The above results showed that the GAD/MC hydrogel in combination with mild PTT could effectively promote endogenous bone repair and reconstruction under DM conditions, which was correlated with the capacity of the photoactivated hydrogel system to create favorable osteogenic and anti-inflammatory microenvironments.

To further investigate the details of the bone healing progress, histological examination, including H&E and MST staining, was performed. As displayed in **Figure 11J-K**, the bone sections showed that a significantly greater amount of calcified new bone and bone marrow cavities formed in the GAD/MC+NIR group than in the GAD/MC and GAD groups at both weeks 4 and 8, which was consistent with the results of the micro-CT analysis. Additionally, a large strip of newly formed bone lacunae and abundant central canals coupled with well-structured lamellar bone tissue were detected in the GAD/MC+NIR group, demonstrating its remarkable performance in accelerating bone regeneration and reconstruction. More importantly, the whole defect area was almost completely filled with newly formed osseous and collagen tissues at 8 weeks with a thickness close to that of the host bone, achieving outstanding therapeutic efficacy in promoting diabetic bone defect healing. In contrast, a considerable amount of fibrous connective tissue, accompanied by the invasion of inflammatory cells, appeared in the blank control group, with no signs of healing at all. The second-best reparative effect was observed in the GAD/MC hydrogel group, followed by the GAD hydrogel group. It was noted that the GAD/MC hydrogel injected into the bone defect was no longer visible after 8 weeks, suggesting suitable degradation properties, which allowed the hydrogel to match the bone repair process. The above validation results of bone repair *in vivo* are consistent with the *in vitro* cellular experiments, suggesting that the photoactivated hydrogel system is a promising injectable therapeutic platform for resolving the complex pathological environment of diabetic bone defects, which may be closely related to its remarkable antioxidant, anti-inflammatory, osteoimmunomodulatory, pro-osteogenic and proangiogenic bioactivities (**Figure 11L**). Additionally, the high porosity and 3D ECM-like features of the hydrogel may also contribute to its notable osteogenic efficacy in bone defect repair. In summary, these radiological and histological results above underscore the potential of the photoactivated GAD/MC hydrogel system to enhance endogenous bone regeneration, even in the presence of diabetic inflammatory conditions.

### *In vivo* modulation of inflammation and the regenerative microenvironment

The key to the success of bone repair under DM conditions lies in modulating the balance of the M1/M2 phenotypes of macrophages, suppressing ROS-induced inflammation, and coordinating subsequent osteoblast differentiation, revascularization, and bone remodeling 90. During the acute stage of diabetic bone healing, the harsh microenvironment formed by the elevated levels of proinflammatory cytokines resulting from the overactivation of M1-type macrophages and the excessive accumulation of ROS at the injury site caused by various mechanisms is the main reasons for cellular damage and disrupted signaling, as well as the impaired differentiation ability of resident cells and disordered immune homeostasis. The resulting vicious cycle of inflammation and oxidative stress usually creates an unfavorable environment for vascularization and tissue repair, ultimately leading to delayed healing and nonunion of bone defects. In contrast, M2-type macrophages have positive effects on cytoprotection and inflammation reduction by secreting anti-inflammatory and pro-regenerative cytokines, such as IL-10, BMP-2, and VEGF, which contribute to tissue regeneration and osteo/angiogenesis promotion 24.

**Figure 12:**
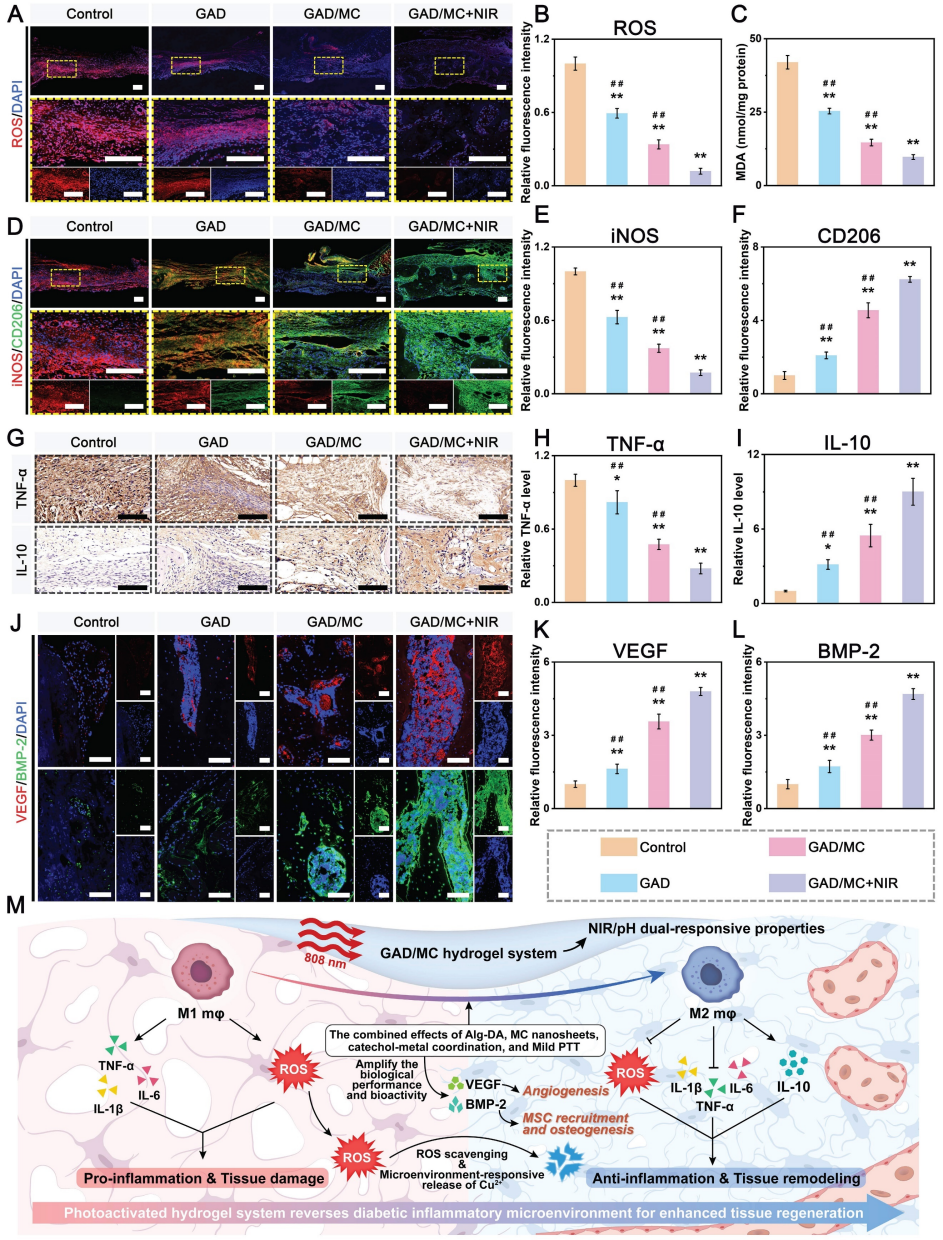
***In vivo* immune regulation and regenerative microenvironment remodeling of the defect region under DM conditions. (A-B)** Representative images and quantitative analysis of DHE staining in different treatment groups. Scale bar: 200 μm.** (C)** MDA levels in the different treatment groups. **(D-F)** Representative immunofluorescence staining images and quantification of iNOS and CD206 in different treatment groups. Scale bar: 200 μm.** (G-I)** Representative immunohistochemical staining images and quantification of TNF-α and IL-10 in different treatment groups. Scale bar: 100 μm.** (J-L)** Representative immunofluorescence staining images and quantification of BMP-2 and VEGF in the different treatment groups. Scale bar: 100 μm.** (M)** Schematic illustration of the mild photothermal-reinforced hydrogel system breaking the vicious cycle in the diabetic microenvironment and achieving optimized tissue regeneration. Data are presented as the mean ± SD (n = 3). *P < 0.05 and **P < 0.01 indicate significant differences compared with the control group. ^#^P < 0.05 and ^# #^P < 0.01 indicate significant differences compared with the GAD/MC+NIR group.

Therefore, to comprehensively investigate the bioregulatory effects of the GAD/MC hydrogel system on ROS scavenging, the immune response, and osteogenesis and angiogenesis, a series of immunofluorescence and immunohistochemical staining experiments were performed. Herein, we first evaluated ROS levels and the inflammatory response within the defect site after different treatments. Dihydroethidium (DHE, a ROS probe) staining was employed for intracellular superoxide labeling to monitor the levels of intracellular ROS in the defect site of diabetic rats **(Figure 12A)**. Compared with the strong fluorescence observed in the control group, the results revealed dramatically reduced ROS levels in the hydrogel-treated groups, indicating that the composite hydrogels effectively mitigated ROS accumulation. In particular, for the GAD/MC group, incorporating MC nanosheets was considered pivotal for reducing ROS levels and alleviating oxidative stress in diabetic rats, indicating its remarkable antioxidant capacity *in vivo*. Furthermore, when subjected to NIR irradiation, the GAD/MC+NIR group displayed even lower levels of ROS **(Figure 12B)**, suggesting exceptional ROS scavenging capabilities with the aid of NIR-mediated mild thermal effects, consistent with the *in vitro* results. It is worth noting that the long-term inflammatory environment in the state of diabetes, along with acute trauma, induces excessive ROS accumulation and oxidative stress, thus aggravating local inflammation and impairing cellular activity 91. Accordingly, antioxidative therapy for resistance to oxidative stress-induced damage has attracted substantial attention for bone regeneration under diabetic conditions 52. To further investigate the oxidative stress state at the defect sites, the level of MDA, an important indicator of cellular oxidative stress damage, was evaluated, and the results are shown in **Figure 12C.** Interestingly, the application of the GAD/MC hydrogel remarkably alleviated the state of local oxidative stress, and this antioxidant ability was further enhanced by on-demand mild photothermal effect, with a more pronounced decrease in MDA levels observed in the GAD/MC+NIR group. Altogether, these above results demonstrated that our prepared GAD/MC hydrogel system could effectively mitigate ROS accumulation and ameliorate oxidative stress under periodic NIR irradiation, showing great potential for protecting cells and tissues against damage in the high-ROS and inflammatory microenvironments of diabetic bone defect areas. Research has shown that excessive ROS and uncontrolled inflammation prevent the polarization of proinflammatory macrophages (M1) to anti-inflammatory macrophages (M2), especially when dealing with diabetic bone defects 90. To further verify the immunomodulatory effects of the hydrogel system on diabetic bone regeneration, immunofluorescence staining of iNOS and CD206 was performed **(Figure 12D)**. The results revealed a negligible M1 macrophage (iNOS^+^) population in the GAD/MC and GAD/MC+NIR groups compared with the GAD group. However, the majority of M1 macrophages (iNOS^+^) were predominantly in the control group, suggesting a pronounced inflammatory response caused by diabetes and bone injury. Meanwhile, a substantially higher population of M2 macrophages (CD206^+^) was observed in the GAD/MC+NIR group than in the GAD/MC and GAD groups **(Figure 12E-F)**. These findings were in good agreement with the *in vitro* flow cytometry and immunofluorescence staining analyses, which suggested that the NIR-assisted hydrogel system had the best induction effect on M2 macrophages and reversed M1 macrophage polarization in hyperglycemic and inflammatory environments. In the initial stage of bone repair, macrophages can polarize toward the proinflammatory M1 phenotype for immune activation and then transform toward the anti-inflammatory M2 phenotype to participate in bone repair. However, the abnormal proinflammatory microenvironment in diabetic bone defect sites severely hinders bone repair by enhancing osteoclast-mediated bone resorption and inhibiting osteoblast-mediated bone formation 92. The promising anti-inflammatory effect detected in the hydrogel system was beneficial for alleviating tissue damage caused by inflammation and recruiting functional progenitor cells to promote tissue regeneration. For this reason, immunohistochemical staining of TNF-α and IL-10 was performed to further explore the inflammatory state at the defect site in diabetic rats **(Figure 12G)**.

Among various proinflammatory cytokines, TNF-α is known as a major inflammatory cytokine and is expressed at the apex of the inflammatory cascade. TNF-α, which is mainly secreted by M1 macrophages at the defect site in diabetes, is associated with a persistent excessive inflammatory response and hinders the healing process. Conversely, IL-10 is a potent anti-inflammatory factor produced primarily by M2 macrophages that attenuates inflammation and inhibits oxidation, indicating a promising effect on tissue repair 65. Compared with those in the control and GAD groups, the lowest number of proinflammatory TNF-α^+^ cells and the highest number of anti-inflammatory IL-10^+^ cells were detected in the GAD/MC+NIR group **(Figure 12H-I)**, thus revealing the potent anti-inflammatory effects of the GAD/MC hydrogel system during the process of diabetic bone defect healing. These findings strongly supported that the GAD/MC hydrogel system, which possessed the ability to scavenge ROS, effectively alleviated the progressive inflammatory environment of the injured site by upregulating the ratio of M2 macrophages, accompanied by the production of anti-inflammatory cytokines and chemokines, which is promising for promoting the transition of inflammation to the proliferation phase and subsequent bone repair and remodeling under diabetic conditions. In addition, at 4 weeks post-implantation, the expression levels of multiple factors involved in tissue repair, e.g., BMP-2 and VEGF, significantly increased in the GAD/MC+NIR group **(Figure 12J)**, underscoring its potential to promote osteogenesis and angiogenesis. Further quantitative analysis of BMP-2 and VEGF revealed that the GAD/MC hydrogel exhibited outstanding pro-regenerative properties under NIR radiation, dramatically promoting the secretion of osteo/angiogenesis-related factors (BMP-2 and VEGF) **(Figure 12K-L)**, which is in accordance with its powerful *in vitro* antioxidant and immunomodulatory capacities. Studies have shown that M2 macrophages not only secrete anti-inflammatory factors, such as IL-4 and IL-10, effectively alleviating the local inflammatory milieu within the defective area but also produce beneficial cytokines that promote cell migration and osteogenic-angiogenic differentiation, including BMP-2 and VEGF 38. Together, these results demonstrated the good antioxidant and anti-inflammatory activities of the GAD/MC hydrogel system, which could reduce prolonged inflammation and high ROS levels in diabetic bone defect areas, foster macrophage M2 polarization, and simultaneously modulate the secretion of inflammatory factors and growth factors, thereby constructing an instructive osteoimmune microenvironment for subsequent osteogenesis and neo-tissue mineralization **(Figure 12M)**. More importantly, H&E staining of major metabolic organs, including the heart, liver, spleen, lung, and spleen, as well as the serum biochemical analysis results, revealed no apparent pathological abnormalities in the different treatment groups **(Figure S27)**, which further suggested the safety and reliability of the comprehensive treatment strategy based on the GAD/MC hydrogel system.

**Figure 13:**
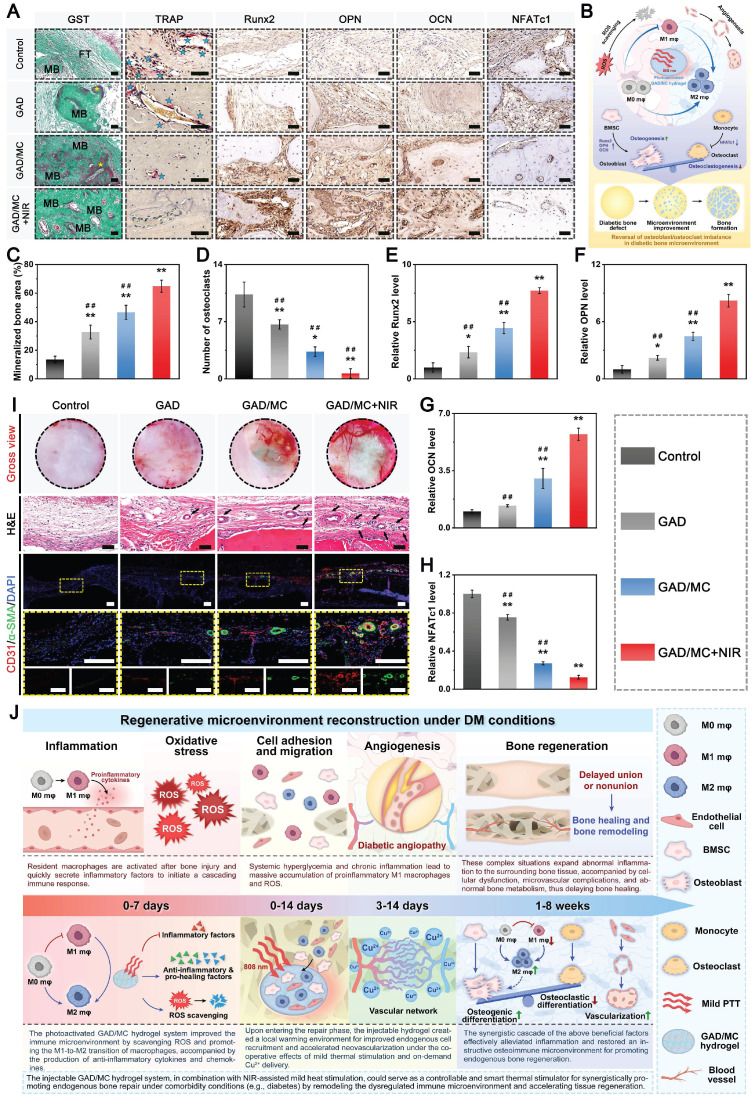
***In vivo* osteogenesis, osteoclastogenesis, and angiogenesis evaluation. (A)** Representative histopathological staining (GST and TRAP) and immunohistochemical staining (Runx2, OPN, OCN, and NFATc1) images. The yellow asterisks represent the osteoid. The blue asterisks represent TRAP-positive cells. Scale bar: 50 μm. **(B)** Schematic illustration of the mechanism of the mild photothermal-reinforced hydrogel system in bone homeostasis under diabetic conditions. **(C)** Quantitative analysis of the mineralized bone area. **(D)** Quantitative analysis of TRAP-positive cells. **(E-H)** Quantitative analysis of Runx2, OPN, OCN, and NFATc1. **(I)** Representative images of neovascularization, H&E staining, and immunofluorescence staining for CD31 and a-SMA in the different treatment groups. The black arrows represent the newly formed blood vessels. Scale bar: 50 μm (H&E staining images) and 200 μm (immunofluorescence staining images). **(J)** Microenvironmental changes after bone defects are disrupted under DM conditions, and the photoactivated GAD/MC hydrogel system alleviates inflammation and restores the regenerative microenvironment for accelerated bone repair *in situ*. After bone injury under DM conditions, the bone defect microenvironment faces adverse conditions characterized by persistent inflammation, excessive oxidative stress, and impaired osteo/angiogenesis, all of which hinder the process of bone defect repair and reconstruction. The application of an injectable and biodegradable hydrogel therapeutic system (GAD/MC), along with adjunct NIR-mediated mild PTT, remarkably mitigates these challenges by reducing inflammation, restoring an imbalanced immune microenvironment, scavenging ROS, inducing the polarization of macrophages from the proinflammatory (M1) phenotype to the anti-inflammatory (M2) phenotype, producing beneficial cytokines for enhanced vascularization and osteogenic differentiation while inhibiting osteoclast function, and ultimately boosting bone regeneration and remodeling. The synergistic cascade of the above beneficial factors meets specific needs across different stages of bone healing, significantly accelerating the healing process of critical-sized bone defects under diabetic conditions. Data are presented as the mean ± SD (n = 3). *P < 0.05 and **P < 0.01 indicate significant differences compared with the control group. ^#^P < 0.05 and ^# #^P < 0.01 indicate significant differences compared with the GAD/MC+NIR group.

As mentioned earlier, bone defect healing and regeneration under diabetic conditions are often compromised by excessive ROS, persistent inflammation, compromised osteo/angiogenesis, and enhanced osteoclast activity. Therefore, eliminating ROS, reducing inflammation, and inducing osteoblast differentiation as well as new blood vessel formation while inhibiting osteoclast function will be conducive to promoting bone repair in patients with diabetes. After confirming the excellent biocompatibility and immunomodulatory function of the hydrogel system and its efficient ROS scavenging capacity, we investigated the effects of the GAD/MC hydrogel on *in vivo* osteogenesis, osteoclastogenesis, and angiogenesis. In particular, the equilibrium between osteoclast-mediated resorption and osteoblast-mediated bone formation plays a pivotal role in maintaining bone homeostasis and promoting bone healing 92. Hence, to further demonstrate the treatment effect of the hydrogel system, we conducted GST staining, TRAP staining, and immunohistochemical analysis to investigate osteoblast and osteoclast function during the process of bone regeneration and remodeling **(Figure 13A)**. At 8 weeks, much more green-stained mature bone tissue was observed in the defect area in the GAD/MC and GAD/MC+NIR groups than in the control and GAD groups. In particular, a larger amount of mineralized tissue with dense and continuous native bone-like structures was observed in the GAD/MC+NIR group, revealing that GAD/MC combined with NIR-mediated mild PTT could provide an optimal microenvironment for osteogenesis and bone mineralization. Quantitative analysis of the mineralized bone tissue area supported the beneficial effects of MC nanosheets and mild PTT on bone regeneration, with the highest proportion of mineralized bone tissue detected in the GAD/MC+NIR group **(Figure 13C)**. TRAP staining was further performed to evaluate the differences in bone resorption among the groups **(Figure 13A)**. As a marker enzyme of osteoclasts, TRAP was significantly upregulated in the control group compared with the other groups, indicating that bone resorption was extraordinarily active under DM conditions.

In contrast, the number of osteoclasts near the bone defect margins in the hydrogel-treated groups was significantly lower than that in the control group, with the highest anti-osteoclast activity found in the GAD/MC+NIR group **(Figure 13D)**, illustrating that the hydrogel system could inhibit the formation of osteoclasts. The data of immunohistochemical staining based on Runx2, OPN, OCN, and NFATc1 further supported this finding **(Figure 13A)**, which showed the ability of the GAD/MC hydrogel system to orchestrate osteoblast/osteoclast balance. Our findings supported the results of GST and TRAP staining, i.e., the positive expression of osteogenic markers (Runx2, OPN, OCN) was more pronounced in the GAD/MC+NIR group, whereas the expression level of NFATc1 was significantly lower **(Figure 13E-H)**. NFATc1, a crucial transcription factor of osteoclastogenesis and osteoclast differentiation, regulates the expression level of TRAP 60. Notably, on-demand NIR irradiation further promoted bone regeneration and increased the expression of osteogenic markers, consistent with the *in vitro* osteogenesis experiment results. Therefore, these results collectively confirmed the potential of the GAD/MC hydrogel as a photoactivated multifunctional therapeutic platform to effectively inhibit bone resorption and enhance bone formation by restoring the osteoblast/osteoclast balance during systemic chronic inflammation-associated bone defects **(Figure 13B)**.

Vascular injury and poor blood supply resulting from diabetic microangiopathy can lead to a lack of oxygen and nutrients, which significantly impairs the tissue regeneration process. Therefore, the activation of endothelial cells and accelerated neovascularization can not only ensure an effective and long-term oxygen supply but also support tissue regeneration by providing adequate nutrients for the cells involved in the healing process 91. There have been reports suggesting that mild heat stimulation can enhance endothelial cell proliferation and migration and subsequent angiogenesis through the VEGF and HSP90/eNOS signaling pathways 93. Simultaneously, Cu elements have also been extensively documented for their ability to stimulate angiogenesis and endothelial cell migration by targeting various proangiogenic markers, including VEGF, HIF-1ɑ, bFGF, Ang-1, and eNOS 73. Encouraged by the proangiogenic effect of the hydrogel system *in vitro*, we further applied immunofluorescence staining of CD31 and a-SMA to characterize neovascularization *in vivo* at 8 weeks **(Figure 13I)**. CD31 and a-SMA serve as important markers for smooth muscle cells and endothelial cells of mature blood vessels, respectively, and were selected to demonstrate the development of blood vessels at the local injury site 87. In accordance with the results of the *in vitro* angiogenic assay, the expression of CD31 and α-SMA dramatically increased after hydrogel treatment, with accelerated neovascularization observed in the GAD/MC group, which was mainly attributed to the stimuli-responsive release of proangiogenic Cu^2+^ ions in a local weakly acidic environment. Notably, this stimulatory effect was further enhanced with the help of mild hyperthermia, and a significant increase in blood vessel density within the defect areas was generated in the GAD/MC+NIR group, as demonstrated by well-defined circular mature vessel structures. The results of the quantitative analysis indicated that the expression of CD31 and α-SMA was significantly increased by the GAD/MC hydrogel plus on-demand NIR irradiation, which is consistent with the results of subcutaneous implantation in mice. According to the results of the macroscopic observation and H&E staining, we found the same angiogenesis tendency as that in the immunofluorescence staining assay. The GAD/MC+NIR group had obvious neovascularization with vascular branches and improved blood supply in the defect area, indicating that the GAD/MC hydrogel combined with mild PTT created an instructive microenvironment for inducing angiogenesis in the process of bone repair. The above *in vivo* results provide us with a more comprehensive understanding of the essential role of the photoactivated hydrogel system in accelerating bone tissue regeneration *in situ*: the GAD/MC hydrogel in conjunction with on-demand NIR irradiation induced substantial mature woven bone tissue (green-stained area) deposition, coupled with abundant osteocytes/osteoblasts and enhanced neovascularization as well as few TRAP-positive osteoclasts in the defective areas after implantation. These results also suggested that the hydrogel system with the function of improving the harsh microenvironment and restoring the osteoblast/osteoclast balance could promote the repair and reconstruction of critical-sized bone defects under DM conditions.

Prior research has demonstrated that mild photothermal stimulation at around 43 °C is instrumental in promoting blood circulation and endothelial cell angiogenesis, leading to enhanced bone defect repair 85. The synergistic action of the responsive release of Cu^2+^ ions and mild thermal stimulation substantially improved local cellular activity and vascularization, resulting in an optimal proangiogenic effect at defect sites. Besides, the GAD/MC hydrogel system could play an osteoimmunomodulatory role by inducing the polarization of macrophages toward the M2 phenotype and then promoting the expression of anti-inflammatory and pro-healing cytokines (e.g., BMP-2 and VEGF), which may be potentially beneficial triggers for enhanced osteogenesis and angiogenesis during the process of diabetic bone defect healing. More importantly, the improved blood supply to the injury sites further facilitated osteoblast function, including proliferation, differentiation, and subsequent biomineralization, which was in accordance with the results of the cellular-level assays. Collectively, these above results revealed that the GAD/MC hydrogel system has a robust ability to accelerate angiogenesis and is more conducive to promoting the reconstruction of the functional vascular network, which can provide sufficient nutrients and oxygen transmission pipelines for the regeneration of large bone defects under diabetic conditions. The potential promoting effect of the photoactivated GAD/MC hydrogel system on diabetic bone regeneration can be attributed to the following factors **(Figure 13J)**: 1) The injectable GAD/MC hydrogels with multiple functionalities are porous and biodegradable materials that can provide a 3D ECM-mimicking microenvironment for cell adhesion, growth, migration, spreading, and differentiation. 2) During the initial phase of bone repair, the photothermal GAD/MC hydrogel system produces mild hyperthermia under on-demand NIR irradiation, which efficiently eliminates excessive ROS, polarizes macrophages toward the pro-regenerative M2 phenotype, enhances the expression of anti-inflammatory and pro-healing cytokines (IL-10, BMP-2, and VEGF) and inhibits the expression of proinflammatory cytokines (TNF-α and IL-6). Additionally, previous studies have shown that mild hyperthermia-assisted ROS scavenging can block the activation of the NF-κb signaling pathway under severe diabetic conditions, which can reduce inflammation and remodel the imbalanced immune microenvironment, thus ending the vicious bone microcirculation induced by diabetes-related chronic inflammation and oxidative stress 64. The synergistic cascade of the above beneficial factors actively induces chemotaxis of various progenitor cells and macrophages in angiogenesis and immunomodulation processes, thus driving the M1-to-M2 transition and tissue regeneration. 3) Upon implantation *in vivo*, the GAD/MC hydrogel system achieves the intelligent release of bioactive Cu^2+^ ions in response to the weak acidity of the diabetic microenvironment and the thermal stimulation generated by on-demand NIR irradiation. The combined impact of intelligent Cu^2+^ ion release and mild heat stimulation could help prevent bacterial infection, stimulate endothelial cell migration, and upregulate the expression of angiogenic factors, thereby promoting angiogenesis and facilitating the transition from the inflammation phase to the repair phase. 4) During the repair and remolding phase, the combined application of the GAD/MC hydrogel with mild heat stimulation synergistically promoted endogenous stem cell recruitment and differentiation and maintained bone homeostasis through accelerating ECM deposition and bone mineralization while suppressing osteoclastogenesis at the defect site. Studies have indicated that mild hyperthermia is conducive to the uptake of blood circulation drugs for cell function, induces the upregulation of HSPs, evokes autoimmune modulation, blocks proinflammatory cascade reactions, and cooperates with osteoblasts and endothelial cells for bone regeneration and reconstruction 30. Moreover, the synergistic effect of the GAD/MC hydrogel and mild PTT accelerated bone healing, thus minimizing the duration of wound exposure and the risk of reinfection. Overall, the comprehensive repair strategy based on the mild photothermal-assisted GAD/MC hydrogel system provides a new option for the clinical treatment of diabetic bone defects, and this strategy can also be used to treat other bone defects and inflammatory diseases caused by diabetes. We demonstrated that it disrupted the vicious cycle of excess ROS and inflammation in the diabetic microenvironment; induced rapid cell recruitment; reversed the early imbalanced immune response by facilitating the M1-to-M2 transition of macrophages; promoted the upregulation of anti-inflammatory and pro-healing cytokines and the downregulation of proinflammatory cytokines; and stimulated angiogenesis, osteogenesis, and mineralization, while inhibiting bone resorption, ultimately achieving accelerated bone defect healing. In the future, we will further investigate the application of a comprehensive repair strategy based on mild photothermal-assisted GAD/MC hydrogels for treating infected bone defects and diabetic-infected bone defects.

## Conclusion

In summary, a multifunctional injectable hydrogel system (GAD/MC) with favorable immunomodulation and an improved regenerative microenvironment was rationally designed and successfully fabricated by considering the characteristics of critical-sized bone defects under comorbidity conditions (e.g., diabetes), clinical requirements, and existing problems associated with current materials. The results revealed that the GAD/MC hydrogels synthesized from GelMA, Alg-DA, and MC nanosheets through multiple covalent and noncovalent interactions could exhibit unique functional characteristics (injectable, self-healing, adhesive, and photothermal properties) and biological functions (antioxidant, anti-inflammatory, antibacterial properties). This injectable hydrogel system exhibited stimuli-responsive release of bioactive Cu^2+^ under a slightly weakly acidic milieu and NIR laser irradiation, as well as remarkable pro-osteogenic and proangiogenic effects in hyperglycemic and inflammatory environments *in vitro*. In addition, the engineered hydrogel system could reverse the harsh microenvironment both *in vitro* and *in vivo* by eliminating excessive intracellular ROS and reducing oxidative stress, as well as by polarizing macrophages toward the M2 phenotype, ultimately leading to a dysregulated immune response and accelerating tissue regeneration. Through the chemoattraction and M2 polarization of macrophages, the factor-free hydrogel system effectively enhanced the crosstalk between macrophages and osteoblasts/endothelial/osteoclast cells, thus forming a positive feedback loop to mitigate the pathological milieu. More significantly, NIR-mediated on-demand mild hyperthermia can further amplify the biological performance and bioactivity of the hydrogel system, thereby enhancing the regeneration and healing process of diabetic bone defects. The convenient preparation process and simple composition of GAD/MC without additional drug or growth factor loading highly facilitate its clinical application. Overall, these findings provide an effective and safe strategy for designing and fabricating multifunctional injectable hydrogels with favorable immunomodulatory activity and accelerated tissue regeneration, which may have significant application prospects in the field of bone tissue engineering.

## Materials and methods

### Materials

Gelatin, sodium alginate, methacrylic anhydride, lithium phenyl (2,4,6-trimethylbenzoyl) phosphinate (LAP), dopamine hydrochloride (DA), layered ternary carbide (Ti_3_AlC_2_) powder, hydrochloric acid (HCl), lithium fluoride (LiF), lipopolysaccharide (LPS), D-glucose, and tris(hydroxymethyl) aminomethane (Tris) were purchased from Sigma-Aldrich Trading Co., Ltd. (Shanghai, China). 1-Ethyl-3-(3-dimethylaminopropyl) carbodiimide hydrochloride (EDC) and N-hydroxysuccinimide (NHS) were purchased from Aladdin Co., Ltd. (Shanghai, China). Copper sulfate (Cu(NO_3_)_2_) and streptozotocin (STZ) were obtained from Macklin Biochemical Technology Co., Ltd. (Shanghai, China). Fetal bovine serum (FBS), high-glucose Dulbecco's modified Eagle's medium (DMEM), alpha-modified Eagle's medium (α-MEM), phosphate-buffered saline (PBS), trypsin-EDTA, and penicillin/streptomycin (P/S) were purchased from Gibco Life Technologies Co. (Grand Island, USA) for *in vitro* cell culture. Mouse calvaria-derived MC3T3-E1 preosteoblastic cells, human umbilical vein endothelial cells (HUVECs), and macrophages (RAW264.7 cells) were obtained from the Institute of Biochemistry and Cell Biology of the Chinese Academy of Sciences (Shanghai, China). A cell counting kit-8 (CCK-8) was acquired from Dojindo Laboratories (Kumamoto, Japan). *Staphylococcus aureus* (*S. aureus*, ATCC 6538) and *Escherichia coli* (*E. coli*, ATCC 25922) were obtained from the Guangdong Microbial Culture Collection Center. The live/dead cell staining kit was purchased from BestBio Biotechnologies (Shanghai, China). Triton X-100 (Sigma-Aldrich), DAPI (Sigma-Aldrich), and TRITC-labeled phalloidin (Invitrogen) were used for cell staining. 2,2-Diphenyl-1-picrylhydrazyl (DPPH) and 2,2′-azino-bis (3-ethylbenzothiazoline-6-sulfonic acid) (ABTS), dihydroethidium (DHE), 2′,7′-dichlorodihydrofluorescein diacetate (DCFH-DA) assay kits, TRIzol RNA extraction kits, 5-bromo-4-chloro-3-indolyl phosphate/nitro blue tetrazolium (BCIP/NBT) alkaline phosphatase (ALP) color development kits, BeyoClick™ EdU-555 imaging kits, bicinchoninic acid (BCA) protein assay kits, and lipid peroxidation malondialdehyde (MDA) assay kits were purchased from Beyotime Biotechnology Co., Ltd. (Jiangsu, China). The ALP assay kit was obtained from Jiancheng Biotech Institute (Nanjing, China). All the chemicals were used without further purification. The water used in all the experiments was purified via a Milli-Q cycle purification system (Millipore, USA).

### Synthesis and characterization of GelMA, Alg-DA, and MC nanosheets

Both GelMA and Alg-DA were synthesized according to our previously reported procedures 33. For the preparation of GelMA, 5 g of gelatin was dissolved in 50 mL of deionized (DI) water and then stirred in an oil bath at 50 °C until a homogeneous gelatin solution (10% w/v) was obtained. Subsequently, 8 mL of methacrylic anhydride was introduced dropwise into the above gelatin solution and allowed to react at 50 °C under constant stirring. The reaction mixture was subsequently transferred to a dialysis bag at 40 °C and dialyzed against DI water to remove the unreacted methacrylic anhydride. After 5 days, GelMA was obtained by lyophilization and stored for further use. The grafting rate was determined to be 45.45% by proton-1 nuclear magnetic resonance (^1^H-NMR).

For the preparation of Alg-DA, sodium alginate (2 g) was dissolved in 100 ml of DI water at a concentration of 2% (w/v) to obtain a homogeneous solution. After that, NHS (0.5755 g), EDC (0.9585), and DA (1.5473 g) were successively added to the above solution. The reaction was stirred for 24 h at room temperature. Afterward, the above mixture was exhaustively dialyzed against DI water at 4 °C for 3 days. Ultimately, the resultant product was obtained via lyophilization and stored for further use. The grafting rate was determined to be 57.52% via ^1^H-NMR.

For the preparation of the MXene nanosheets, 3.2 g of LiF was dissolved in 40 mL of 9 M HCl and stirred in a Teflon agitator at 40 °C for 40 min to ensure the dissolution of LiF. Afterward, 2 g of Ti_3_AlC_2_ powder was gradually dispersed into the above solution with continuous stirring at 40 °C for 48 h. The reaction mixture was repeatedly rinsed with DI water until the pH of the solution reached neutral. Subsequently, the raw MXene nanosheets were obtained via centrifugation at 3000 rpm for 25 min, washed with DI water three times, and freeze-dried. To synthesize the Cu-decorated MXene nanosheets, an aqueous solution of Cu(NO_3_)_2_ (0.5%, w/v) and the as-prepared MXene suspension (0.5 mg/mL) were mixed at equal volume ratios, and the above mixture was sonicated for 5 min, followed by continuous stirring for 12 h at room temperature. Afterward, the resulting products were collected by centrifugation, washed repeatedly with DI water to remove unattached Cu^2+^ ions, and then freeze-dried for further use and characterization.

The chemical structures of the modified gelatin and alginate were evaluated via ^1^H-NMR (NMR, AV500 MHz, Bruker, Switzerland) using deuterium oxide (D_2_O) as the solvent. The morphology and elemental distribution of the nanosheets were determined via field emission scanning electron microscopy (FE-SEM, Zeiss, SIGMA, Germany) and transmission electron microscopy (TEM, JEM2100, Hitachi, Tokyo, Japan) equipped with energy-dispersive spectroscopy (EDS). The thickness of the nanosheets was observed via atomic force microscopy (AFM, Dimension Icon, USA). The chemical composition and state were analyzed via X-ray diffraction (XRD, D8A A25 X, Bruker, Germany) and X-ray photoelectron spectroscopy (XPS, ESCALAB 250XI, Thermo Scientific, New York). The -ultraviolet-visible-near-infrared (-UV-vis-NIR) absorption spectra were obtained with a UV-vis spectrophotometer (UV-2600i, Shimadzu, Japan). Thermogravimetric (TG) analysis was conducted via a thermal analyzer (Diamond TG/DTA; PerkinElmer Instruments, Shanghai, China). To investigate the photothermal-responsive ion release from the MC nanosheets, the prepared samples were dispersed in 1 mL of DI water and incubated at 37 °C on a shaker bed at 70 rpm. For NIR treatment, the MC nanosheet suspension was exposed to daily periodic NIR irradiation (808 nm, 1 W/cm^2^) for 5 min. At predetermined time points, the suspension was centrifuged, and the concentrations of Cu^2+^ ions were measured via an inductively coupled plasma optical emission spectrometer (ICP-OES, Agilent 5110).

### Screening of the optimal MC concentration

In this study, MC3T3-E1 preosteoblastic cells and HUVECs were used to investigate the biocompatibility and biological performance of synthesized MC nanosheets. First, MC3T3-E1 cells and HUVECs were cultured in complete DMEM supplemented with 10% (v/v) FBS and 1% P/S. All the cells were incubated at 37 °C under 5% humidified CO_2,_ and the medium was changed every 2 days. When the cells reached 80-90% confluence, they were trypsinized for subsequent experiments. To evaluate the cytotoxicity of the nanosheets, MC3T3-E1 cells and HUVECs were seeded in 96-well plates at a density of 3×10^3^ cells/well and incubated for 24 h. Then, the medium was replaced with 200 μL of culture medium containing different concentrations of MC nanosheets (0-120 μg/mL). At predetermined time points, the medium was replaced with a CCK-8 mixture (10% CCK-8 solution and 90% DMEM) and incubated at 37 °C in the dark for 2 h. Finally, the absorbance of the solution at 450 nm was measured via a microplate reader (Multiskanfc, Thermo Scientific). After being incubated for 3 days, the cells were washed gently and stained via a live/dead cell staining kit to observe the live and dead cells. Fluorescence images were acquired with an inverted fluorescence microscope (Olympus IX73, Tokyo, Japan).

To evaluate the osteogenic activity of the nanosheets, MC3T3-E1 cells were seeded in 24-well plates at a density of 4×10^4^ cells/well and cultured for 24 h to allow them to attach fully. The culture medium was then changed to an osteogenic induction medium containing different concentrations of MC nanosheets (0-120 μg/mL). The medium was refreshed every 2 days. After being treated with or without MC nanosheets for 7 days, the cells were stained with a BCIP/NBT ALP color development kit, and ALP activity was also detected with an ALP assay kit following the manufacturers' instructions. After 14 days, the fixed cells were treated with ARS staining solution, and images were obtained under an optical microscope. Then, 10% cetylpyridinium chloride was added to each well, and the absorbance of the resulting solution at 562 nm was detected via an ultraviolet-visible (UV-vis) spectrophotometer (Perkin Elmer, USA).

The proangiogenic activity of the MC nanosheets was evaluated via a tube formation assay. Briefly, HUVEC suspensions containing different concentrations of MC nanosheets (0-120 μg/mL) were seeded in Matrigel-coated plates and incubated at 37 °C for 8 h. Subsequently, the tube structures were observed and imaged under an inverted microscope (Leica, Germany). The number of junctions and total branching length were quantified via ImageJ software. Additionally, a Transwell assay was conducted following a previous study to evaluate the migration of HUVECs after various treatments. Briefly, HUVECs were seeded in the upper chamber with a serum-free medium. Then, 500 μL of complete medium containing different concentrations of MC nanosheets (0-120 μg/mL) was added to the lower chamber. After incubation for 24 h, the HUVECs on the lower surface of the membrane were fixed with 4% paraformaldehyde, stained with 0.1% crystal violet, and then observed under an optical microscope. The number of migrated HUVECs was analyzed via ImageJ software.

### Synthesis and characterization of GAD/MC hybrid hydrogels

To prepare the GAD/MC hydrogel, GelMA (700 mg) and Alg-DA (300 mg) were dissolved in DI water (10 mL) to obtain a homogeneous GAD solution. Then, the LAP photoinitiator (0.5% w/v) was added to this mixture and thoroughly mixed while ensuring that the mixture was protected from light. According to preliminary screening results, the as-fabricated MC nanosheets (300 μg) were mixed with the above mixture, followed by ultrasonication to obtain a GAD/MC suspension. Afterward, the hydrogel precursor solution was transferred to customized reaction models and then stirred until the hydrogel formed. The resulting GAD/MC hydrogel was subsequently placed into specific crosslinking ovens and exposed to UV irradiation for photo-polymerization. Meanwhile, the hydrogel precursor solution without the addition of MC nanosheets was used to prepare the GAD hydrogel as a control group via the same process described above.

**Physicochemical characterization:** The morphology and microstructure of the materials were examined via FE-SEM. EDS (UltimMax 40, Oxford, UK) was used to obtain elemental mapping images. A microcomputed tomography system (micro-CT, SkyScan 1276, Bruker, Germany) was used to characterize the porous structure in 3D to analyze the porosity and pore size distribution 60. The mechanical properties of the hydrogels were measured via a universal testing machine (CMT6503, Shenzhen SANS Test Machine, China) with a compression rate of 1 mm/min. Rheological tests were performed with a rheometer (Kinexus, Malvern) using parallel plates 25 mm in diameter. TG analysis was implemented via a thermal analyzer with a heating rate of 10 °C/min under a N_2_ atmosphere. The hydrophilicity of the hydrogel was determined by an optical contact angle measuring device (PT-7058, Precise Test, China).

**Photothermal performance:** The photothermal properties of the hydrogels were evaluated by an 808 nm NIR laser (KS-810F-8000, Kai Site Electronic Technology, China). First, all the samples were placed at the bottom of a 24-well plate and immersed in 1 mL of PBS solution. After irradiation at different power densities (0, 0.5, 1.0, and 1.5 W/cm^2^), the real-time temperature changes in the hydrogel samples were recorded by a FLIR ONE thermal imager (FLIR Systems, Inc., Wilsonville, OR). To evaluate the photothermal stability, the hydrogel samples were irradiated with an 808 nm NIR laser at different power densities (0, 0.5, 1.0, and 1.5 W/cm^2^) for five consecutive cycles, after which the thermal curves were recorded. For *in vivo* evaluation of the photothermal properties, the GAD/MC hydrogel was injected into the backs of BALB/c mice (male, 8 weeks old) and irradiated with an 808 nm NIR laser at a power density of 1.5 W/cm^2^ for 5 min. Photothermal photographs and cooling curves were recorded with an infrared thermal imaging camera.

**Responsive release of Cu^2+^ ions *in vitro*:** The release kinetics of Cu^2+^ from the hydrogel were investigated under various conditions. Briefly, the GAD/MC hydrogels were incubated in PBS at different pH values (6.5 and 7.4) and maintained at 37 °C. At different time points, the supernatant was collected and replenished with the same volume of fresh buffer. Moreover, the GAD/MC hydrogel suspension was treated daily with periodic NIR irradiation (808 nm, 1.5 W/cm^2^) for 5 min. The samples not exposed to NIR irradiation served as controls. The release of Cu ions from the hydrogels was determined via ICP-OES.

**Mechanical performance:** The rheological properties of the hydrogel samples were measured via a rheometer (Kinexus, Malvern) under an oscillation-frequency sweep model. To obtain the relevant storage modulus of the hydrogel samples (G') and loss modulus (G''), the samples were prepared into suitable sizes and placed on parallel plates, which were carried out at frequencies of 0.1 to 20 Hz with a constant strain of 0.5% at room temperature. For compression analysis, the samples were tested by a universal testing machine (CMT6503, Shenzhen SANS Test Machine, China) with a compression rate of 1 mm/min. The compressive strength was obtained from stress-strain curves, and the compressive modulus was calculated from the slope of the linear region (i.e., 0%-15%) of the stress-strain curve.

***In vitro* swelling and biodegradation behaviors:** To study the swelling properties of the hydrogels, all the prepared hydrogel samples were weighed (W0) before being immersed in PBS (pH = 7.4) at 37 °C. At different time points, the swollen hydrogel samples were removed from the solution and weighed (W1) after the excess water on the surface of the hydrogels was removed via filter paper. The swelling ratio was calculated via the following equation: Swelling ratio = (W1-W0)/W0 × 100%. The change in the size of the hydrogels in the wet state was calculated by measuring the radius before (D0) and after (D1) they reached swelling equilibrium. The swelling ratio was calculated via the following equation: size change ratio = (D1-D0)/D0 × 100%. For the biodegradation test, the lyophilized hydrogel samples were immersed in PBS (pH = 7.4) containing 2 U/mL protease (Type XIV from Streptomyces griseus (3.5 U/mg)) at 37 °C with constant shaking (100 rpm). The initial hydrogel sample was accurately weighed (M0). At predetermined time intervals, the hydrogels were removed from PBS and weighed (M1). The weight remaining of the hydrogel was calculated via the following formula: mass remaining = M1/M2 × 100%.

**Injectability, moldability, and self-healing ability assays:** To investigate injectability and moldability, the hydrogel was loaded into a syringe with a 26-gauge needle and then extruded through the needle directly into molds of different shapes. To evaluate the moldability of the hydrogels, the prepared hydrogel precursor solution was injected into molds of various shapes to produce complex geometries under UV exposure. Photos were taken to record the process of injection and the appearance of the hydrogel. The self-healing performance of the GAD/MC hydrogel was investigated by both macroscopic observation and SEM. Briefly, the hydrogels were cut in half along the middle with a clean blade, and then their cut surfaces were put together to heal without any external stimuli. After being maintained at room temperature overnight, the self-healing behaviors of the hydrogels were confirmed through repeated stretching performance and SEM evaluation. To further evaluate the self-healing efficiency of the GAD/MC hydrogel, rheological tests were performed as previously described 5.

**Bone tissue adhesion test:** The bone adhesive capacity of the hydrogels was examined by using fresh bone pieces. Briefly, cranial bone pieces were obtained from the rats and further cut into irregular smaller pieces, followed by immersion in PBS before use. The contact portion of each piece was coated with a thin layer of hydrogel precursor around it and overlapped in close contact with the rest of the pieces, which were then placed under UV irradiation for polymerization and photographed. The classical lap shear test was used to evaluate the adhesive strength of the hydrogels at a speed of 10 mm/min 19.

### *In vitro* evaluation of antibacterial activity

In this work, Gram-positive *S. aureus* and Gram-negative *E. coli* were selected as model bacteria for the following antibacterial experiments. First, to achieve the desired concentration of bacterial suspension, *S. aureus* and *E. coli* were cultivated in Luria-Bertani (LB) liquid media for 24 h at 37 °C in a shaker. For the standard plate experiment, 200 μL of hydrogel was injected into 24-well cell culture plates, and then 50 μL of the bacterial solution (1 × 10^5^ CFU/mL) was added to each hydrogel surface by dropping, followed by the addition of 950 μL of liquid medium. In addition, for the NIR group, the sample was irradiated with an NIR laser (808 nm, 1.5 W/cm^2^) for 5 min every 8 h. After incubation for 24 h, the supernatants from the different groups were diluted 10^4^ times and then evenly spread onto LB plates (1.5% agar). The colonies on the plate were counted and photographed after 24 h of incubation. Moreover, to evaluate bacterial viability, the supernatants of the treated bacterial suspensions in different groups were collected, and the absorbance at 600 nm was measured via a UV-vis spectrophotometer (Perkin Elmer, USA). The turbidity changes in the treated bacterial suspensions were photographed.

For live/dead bacterial staining, the bacteria collected after various treatments were stained with the LIVE/DEAD BacLight Bacterial Viability Kit (Invitrogen, USA) for 20 min and then observed under a confocal laser scanning microscope (CLSM; TCS SP8, Leica, Germany). For SEM observation, the treated bacteria were fixed with 2.5% glutaraldehyde at 4 °C overnight and then dehydrated through a graded ethanol series, followed by drying in a vacuum oven for 24 h. After being coated with gold, the bacterial morphologies were observed via SEM.

For the anti-biofilm assay, the hydrogels were incubated with bacterial suspensions in a 24-well plate at 37 °C to form mature biofilms. After various treatments, the samples were gently washed twice with PBS and stained with 0.1% crystal violet solution for 20 min. To quantify the anti-biofilm ability, the stained samples were dissolved in 95% ethanol solution and measured via a UV-vis spectrophotometer at an absorbance of 590 nm. To observe the 3D structure of the bacterial biofilm, the samples were stained with the LIVE/DEAD BacLight Bacterial Viability Kit and directly observed via CLSM.

### *In vitro* evaluation of cytocompatibility and osteoinductivity

The cytocompatibility of the hydrogels was evaluated via the indirect contact method and direct contact method 94. For the indirect coculture system, MC3T3-E1 cells at a density of 2 × 10^4^ cells/well were seeded in the lower chamber of a 24-well Transwell plate with a 0.4 μm pore size (Corning, USA), and the hydrogels (100 μL) were placed in the upper chamber. The well without the upper chamber was used as the blank control. For the direct coculture system, sterilized thin hydrogels (100 μL) were spread on the bottom of 24-well plates, and MC3T3-E1 cells in a good growth state were seeded onto the surface of the hydrogels at a density of 2 × 10^4^ cells/well. During the incubation period, the cells in the GAD/MC+NIR group were exposed to periodic NIR laser irradiation (808 nm, 1.5 W/cm^2^) for 5 min every day to maintain the peak irradiation temperature at 42 ± 1 °C, whereas the other groups were not subjected to NIR irradiation. At predetermined time points, a CCK-8 assay was used to evaluate cell proliferation by measuring the absorbance at 450 nm as described above. After incubation for 3 days, a live/dead staining assay was carried out to assess the viability of the MC3T3-E1 cells. Moreover, the apoptosis assay was performed by using an Annexin V-FITC apoptosis detection kit according to the instructions. The percentage of apoptotic and live cells was detected by flow cytometry (FC500, Beckman Coulter, Fullerton, CA, USA), and the data were analyzed with FlowJo software. To assess cell adhesion, spreading, and infiltration, MC3T3-E1 cells that adhered to the hydrogels were stained with TRITC-labeled phalloidin and DAPI according to the manufacturer's protocol and then imaged via CLSM. Additionally, the adhesion and spreading of MC3T3-E1 cells were further investigated via SEM after CO_2_ critical point drying, as previously described 57.

To evaluate the osteogenic potential of the hydrogels under hyperglycemic and inflammatory conditions, MC3T3-E1 cells were cocultured with various hydrogels in osteogenic induction medium supplemented with D-glucose (25 mM) and LPS (200 ng/mL) as previously described 11. The medium was replaced every 3 days, and cells cultured without the hydrogels were used as controls. In addition, the NIR group was irradiated with 808 nm NIR laser light at 1.5 W/cm^2^ for 5 min following the same protocol as previously described. At scheduled time points, the osteogenic activity of MC3T3-E1 cells was investigated via ALP staining, ECM mineralization, extracellular collagen secretion, and calcium deposition. Briefly, after coculturing for 7 days, the cells in the different groups were washed and fixed with 4% paraformaldehyde. The intracellular ALP activity was evaluated with a BCIP/NBT ALP color development kit according to the manufacturer's instructions. After coculturing for 14 days, ECM mineralization and collagen secretion were detected via ARS and Sirius Red staining, respectively. After coculturing for 21 days, calcium deposition was analyzed via Von Kossa staining under the same culture conditions as those used for ALP staining. Furthermore, BMSCs isolated from the tibiae and femora of 4-week-old male SD rats were incubated in α-MEM supplemented with 10% FBS and 1% P/S at 37 °C in a humidified incubator with 5% CO_2_ as previously described 95. BMSCs at passages 3-5 with good growth (80-90% confluency) were used for subsequent experiments. For *in vitro* osteogenic activity assessment, ALP, Sirius Red, ARS, and Von Kossa staining were performed in the hydrogel-treated cells under DM-related conditions via the same process as previously described.

Furthermore, the expression of osteogenesis-related genes, including ALP, Col-1, Runx2, OPN, and OCN, was assessed by quantitative real-time polymerase chain reaction (qRT-PCR). Total RNA was extracted from cells and reverse transcribed to complementary DNA (cDNA) via the HiScript III RT SuperMix Kit following the manufacturer's protocol. Next, the qRT-PCR experiment was carried out using a 7500 real-time PCR system (Applied Biosystems, USA). The relative gene expression levels were normalized to those of the housekeeping gene GAPDH and calculated via the 2^-ΔΔCT^ method. The sequences of the primers used for each gene are shown in **Table S1**. The expression of Runx2 and OPN was also evaluated by immunofluorescence staining. Briefly, after various treatments, the cells were collected, fixed, and blocked before being stained with antibodies against Runx2 (Cell Signaling, D1L7F) and OPN (ProteinTech, 22952-1-AP) at 4 °C overnight. The cells were subsequently incubated with the corresponding secondary antibodies and counterstained with DAPI for 5 min. Finally, fluorescence images were obtained via CLSM.

### *In vitro* evaluation of antioxidant and immunomodulatory functions

**Antioxidant capacity of the hydrogels:** Both DPPH and ABTS radical scavenging assays were used to evaluate the *in vitro* antioxidant capacity of the hydrogels. For the DPPH radical scavenging capacity assay, the prepared hydrogel samples were mixed with a DPPH ethanol solution (100 µM), followed by incubation for 20 min in the dark at 37 °C. The wavelength of the solution was subsequently determined via a UV-vis spectrophotometer at 517 nm. For the ABTS radical scavenging ability assay, the prepared hydrogel samples were submerged in the ABTS solution, followed by incubation at 37 °C for 10 min. Then, the wavelength of the solution was determined via a UV-vis spectrophotometer at 414 nm.

**Intracellular ROS detection and cytoprotective effects**: To imitate hyperglycemia and the inflammatory environment in individuals with diabetes, RAW264.7 macrophages seeded on hydrogels were incubated in DMEM containing D-glucose (25 mM) and LPS (200 ng/mL) 11. To assess the intracellular ROS scavenging activity, the above-treated RAW264.7 cells were incubated with 10 μM DCFH-DA for 20 min in the dark at 37 °C. Cells grown without hydrogel treatment served as controls. After being rinsed with PBS, the stained cells were observed via CLSM and analyzed via flow cytometry. In addition, MDA, a metabolite of lipid peroxidation, was chosen as an indicator of lipid peroxidation. The concentration of MDA in the treated RAW264.7 cells was measured via an MDA detection assay kit according to the manufacturer's protocol.

The cytoprotective effect of the hydrogel system on cells against oxidative stress was also evaluated. Briefly, except for those in the blank group, RAW264.7 cells were cultured under hyperglycemic and inflammatory conditions and stimulated with D-glucose (25 mM) and LPS (200 ng/mL) added to the culture medium. The treated cells were subsequently seeded on the hydrogel with or without NIR irradiation, and the cells grown without hydrogels served as controls. After coculturing for 1, 2, and 3 days, the absorbance at 450 nm was measured via a CCK8 assay following the instructions provided by the manufacturer. Cell viability was calculated as a percentage relative to the absorbance of the control group. Similarly, live/dead staining and EdU staining assays were further applied to investigate cell proliferation after 3 days according to the manufacturers' protocols.

**Immune response analysis:** After treatment with D-glucose and LPS as mentioned above, macrophage (RAW264.7) suspensions (2 × 10^5^ cells per well) were seeded on various hydrogels with or without NIR irradiation for 3 days. To observe the spreading and infiltration of macrophages cocultured on the hydrogels, the cells were stained with TRITC-labeled phalloidin and DAPI and examined via CLSM as described above. For further observation, the fixed samples were dehydrated through a graded ethanol series and imaged via SEM. Flow cytometry was used to detect the phenotype of the macrophages *in vitro*. Briefly, macrophages were collected and incubated with anti-C86 antibody (BioLegend, 105011) and anti-C206 antibody (BioLegend, 141703) according to the manufacturer's protocol. Finally, macrophage polarization was quantified via flow cytometry and analyzed via FlowJo software. For the immunofluorescence staining assay, the cells were stained with iNOS and CD206 overnight at 4 °C. The stained samples were subsequently observed via CLSM. Furthermore, qRT-PCR was used to detect the expression levels of proinflammatory genes of the M1 type (CD86, IL-6, TNF-α, and iNOS) and anti-inflammatory genes of the M2 type (IL-4, IL-10, Arg-1, and CD206) in RAW264.7 cells after different treatments through the same protocol as described above. The housekeeping gene GAPDH was chosen as the reference gene, and the expression levels of the target genes were analyzed via the 2^-ΔΔCt^ method. The forward and reverse primer sequences are listed in **Table S1**. Inflammation-related cytokines were measured via commercial ELISA kits following the manufacturer's instructions.

### *In vitro* evaluation of angiogenic activity

**Intracellular ROS detection and cytoprotective effects**: To imitate hyperglycemia and the inflammatory environment in individuals with diabetes, HUVECs seeded on hydrogels were incubated in DMEM containing D-glucose (25 mM) and LPS (200 ng/mL) as described above. To assess the intracellular ROS scavenging activity and cytoprotective effect of the hydrogel system, DCFH-DA staining, live/dead cell staining, EdU staining, and CCK-8 assays were subsequently performed according to the manufacturer's instructions.

**Evaluation of angiogenesis:** To investigate the impacts of the hydrogel system on vascularization *in vitro*, a coculture model was established via a Transwell chamber system, as previously described 96. For the Transwell migration assay, 1 × 10^4^ HUVECs were added to the upper chamber of a 24-well Transwell plate and incubated in complete DMEM supplemented with LPS (200 ng/mL) and D-glucose (25 mM) to simulate hyperglycemic and inflammatory conditions. The hydrogels were subsequently placed in the lower chamber containing the same medium as described above. After coculturing for 24 h with or without NIR irradiation (808 nm, 1.5 W/cm^2^), the migrated HUVECs were washed with PBS and fixed with 4% paraformaldehyde for 15 min at room temperature. Finally, the cells were stained with crystal violet for 30 min and observed under an optical microscope. For the wound healing assay, 5 × 10^4^ HUVECs were seeded in 24-well plates and incubated in complete DMEM supplemented with LPS (200 ng/mL) and D-glucose (25 mM). After 24 h, a 20 μL pipette tip was used to create scratches on the cell monolayer. The hydrogels were placed in the upper chambers and cultured in 200 μL of complete DMEM supplemented with LPS and D-glucose. Images of the migrated cells were captured with an inverted fluorescence microscope at 0 and 24 h, and statistical analysis was performed via ImageJ software. The proangiogenic effect was further evaluated via an *in vitro* tube formation assay. Briefly, HUVECs were added to the Matrigel-coated lower chamber of a 24-well Transwell plate and cultured in complete DMEM supplemented with LPS and D-glucose as described above. Simultaneously, the hydrogels were placed in the upper chambers and cultured under the same conditions. After 8 h, the cells were stained with calcein-AM to observe tube formation under an inverted fluorescence microscope. Furthermore, newly formed junctions and meshes were counted to quantitatively analyze the angiogenic effect of the hydrogel system. The expression levels of angiogenesis-related genes, including Ang-1, bFGF, HIF-α, and VEGF, in HUVECs after different treatments were subsequently assessed via qRT-PCR following the above protocols. The 2^-ΔΔCT^ method was used to calculate relative gene expression levels. The sequences of the primers used for this analysis are provided in **Table S1**. Immunofluorescence staining of CD31, VEGF, and HIF-1α was also performed to evaluate the angiogenic effect. Briefly, after various treatments, HUVECs were fixed, permeabilized, and blocked before being stained with antibodies against CD31 (ProteinTech, 11265-1-AP), VEGF (ProteinTech, 66828-1-Ig), and HIF-1α (20960-1-AP) in the dark at 4 °C overnight. Next, the samples were incubated with the corresponding secondary antibodies and DAPI. Finally, the stained cells were observed and photographed via CLSM.

### *In vitro* evaluation of the influence of macrophage polarization on osteogenesis, angiogenesis, and osteoclastogenesis

Conditioned medium was prepared as previously reported 57. Briefly, fresh supernatant from RAW264.7 cells treated with hydrogels with or without NIR irradiation for 3 days, as mentioned above, was initially collected and centrifuged at 3000 rpm for 5 min. Subsequently, the supernatants were subjected to filtration via a 0.22 μm pore size filter to remove any residual cells or hydrogel debris. The concentrations of inflammatory and pro-healing cytokines in the extracts were detected via ELISA according to the manufacturer's protocols. For osteogenic differentiation experiments, MC3T3-E1 cells were cocultured in media composed of collected macrophage culture supernatants and osteogenic induction medium at a ratio of 1:2. After coculturing for 7 and 14 days, ALP activity and ARS staining were conducted following the same process described above. For angiogenic differentiation experiments, HUVECs were cocultured in media composed of collected macrophage culture supernatants and complete DMEM at a ratio of 1:2. Transwell migration and tube formation assays were performed via the same methods described above. For osteoclastic differentiation experiments, mouse bone marrow-derived macrophages (BMMs) isolated from 4-week-old C57BL/6 male mice were cocultured in media composed of collected macrophage culture supernatants and complete α-MEM supplemented with 50 ng/mL receptor activator of nuclear factor-κ B ligand (RANKL) and 30 ng/mL recombinant mouse macrophage colony-stimulating factor (M-CSF) at a ratio of 1:2. After coculturing for 6 days, the cells were fixed with 4% paraformaldehyde and then subjected to F-actin ring fluorescence staining and TRAP staining as previously described. Bone resorption activity was also evaluated on the surface of bovine bone discs as described previously 97. Briefly, BMMs were seeded onto the surface of bovine bone discs and then incubated with the conditioned medium as described above. After 6 days, the bovine bone discs were sonicated for 5 min to remove the cell pellet and then imaged with an optical microscope.

For the immunofluorescence staining assay, polarized macrophages were fixed with 4% paraformaldehyde for 30 min, permeabilized with 0.2% Triton X-100 for 10 min, and then blocked with 5% bovine serum albumin at room temperature for 1 h. Afterward, the treated cells were incubated with primary antibodies against CD206, BMP-2, and VEGF at 4 °C overnight. The cells were subsequently incubated with the corresponding secondary antibodies and counterstained with DAPI for 5 min. Finally, fluorescence images were obtained via CLSM. Finally, fluorescence images were obtained via CLSM. The detailed protocols for western blotting were described in a previous study 30. Briefly, after various treatments, RAW264.7 cells were lysed, and the total protein levels were quantified via a BCA protein assay kit. Equal concentrations of protein were subjected to sodium dodecyl sulfate (SDS)-polyacrylamide gel electrophoresis and then transferred to a polyvinylidene fluoride (PVDF) membrane. The membranes were blocked and incubated with primary antibodies at 4 °C overnight. After being incubated with the HRP-conjugated secondary antibody, protein signals were visualized by a Tanon-5200 chemiluminescent imaging system (Tanon, Shanghai, China).

### *In vivo* subcutaneous implantation study

To evaluate *in vivo* biocompatibility and biological activity, 8-week-old male BALB/c mice were selected for the subcutaneous implantation study. All animal experimental procedures were conducted under supervision and approved by the Animal Care and Use Committee of Wuhan University, and the protocols were in accordance with the Guide for the Care and Use of Laboratory Animals. Briefly, the mice were anesthetized via inhalation of isoflurane. The dorsal skin of each mouse was then shaved and cleaned with povidone/iodine solution. After the dorsal skin was exposed, the prepared hydrogel samples were then implanted into the subcutaneous tissue of the mice, and the overlying muscle and skin were sutured. The animals were randomly assigned to the following treatment groups (n = 3 per group): (1) the GAD group (implanted with GAD hydrogel), (2) the GAD/MC group (implanted with GAD/MC hydrogel), and (3) the GAD/MC+NIR group (implanted with GAD/MC hydrogel plus periodic NIR irradiation). The mice in the GAD/MC+NIR group were exposed to NIR laser irradiation (808 nm, 1.5 W/cm^2^) for 5 min every 2 days to maintain the temperature at 42 ± 1 °C, followed by a natural cooling period. The animals were sacrificed at 2 weeks after treatment, and the *in vivo* implanted hydrogels and surrounding tissue were collected for histological assessment and immunohistochemical staining. After being fixed in 4% paraformaldehyde for 48 h, the collected samples were embedded in paraffin and sectioned for hematoxylin and eosin (H&E) staining. Moreover, to detect inflammation, stem cell recruitment, and blood vessel formation, immunohistochemical staining of iNOS, CD206, CD44, CD90, CD31, and α-SMA was performed. Furthermore, the secretion of immunomodulation-related cytokines from the collected samples was assessed via ELISA following the manufacturer's instructions.

### *In vivo* bone repair study

The *in situ* bone repair capacity of the hydrogels was tested in a critical-sized rat calvarial bone defect model. The surgical procedures were conducted under sterile conditions. Briefly, 8-week-old male SD rats were anesthetized via isoflurane inhalation and randomly divided into control, GAD, and GAD/MC (n = 3 per group) groups. After the dorsal cranium was exposed, two symmetrical full-thickness critical-sized calvarial bone defects (5 mm in diameter) were constructed via a saline-cooled dental trephine under sterile conditions. Then, the prepared hydrogel samples were injected into the defect sites, followed by solidification through UV light irradiation. Finally, the incision was carefully closed layer-by-layer with 4-0 nylon sutures. The untreated group (without hydrogel) served as the control group. To evaluate bone repair under systemic inflammatory conditions, the rats were treated with an intraperitoneal injection of LPS (1 mg/kg) four times at 24 h intervals 1. After 7 days of inflammation induction, the rats were anesthetized with isoflurane gas, followed by disinfection and exposure to the dorsal cranium. A critical-sized cranial defect model was subsequently established, in which the procedures and groups were the same as those used for the animal experiments described above. At 6 weeks after implantation, the rats were sacrificed, and the harvested calvarial bones were fixed in 4% paraformaldehyde for 24 h and then scanned via a micro-CT system. The scanning resolution was 6 μm, and 3D reconstruction was achieved with CTVol software (SkyScan). For quantitative analysis of new bone formation within the defects, the bone mineral density (BMD), bone tissue volume/total tissue volume (BV/TV), bone trabecular thickness (Tb.Th), and trabecular number (Tb.N) were measured. After micro-CT scanning, the samples were decalcified, embedded in paraffin, and sectioned for histological analysis via H&E, Masson's trichrome (MST), and Goldner's trichrome (GST) staining, according to the manufacturer's protocols. The stained sections were examined via a light microscope, and images were captured.

To further study the bone regeneration capacity under comorbidity conditions (e.g., DM), a diabetic rat calvarial bone defect model was established according to previous methods 98. Briefly, 6-week-old SD rats received intraperitoneal injections of STZ (60 mg/kg) until the plasma glucose levels consistently exceeded 16.7 mmol/L for 2 weeks; these rats were considered successful models of diabetes. The animals were maintained in a diabetic state for the bone repair experiment. A total of forty diabetic rats were used in the following study. To establish calvarial defect models, diabetic rats were anesthetized via continuous inhalation of isoflurane. After the surgical field was exposed, two symmetrical calvarial bone defects in diabetic rats were created as described above. The diabetic rats were randomly divided into four groups (10 rats per group): (1) the control group (bone defects without hydrogel implantation), (2) the GAD group (implanted with GAD hydrogel), (3) the GAD/MC group (implanted with GAD/MC hydrogel), and (4) the GAD/MC+NIR group (implanted with GAD/MC hydrogel plus periodic NIR irradiation). The pregel samples were injected into the bone defect site, and gelation was initiated through UV irradiation. For the GAD/MC+NIR group, the defect sites of the rats were subjected to periodic NIR treatment (808 nm, 1.5 W/cm^2^) for 5 min every 2 days. After the successful implantation of the hydrogels, the incision was carefully sterilized and sutured. At 4 and 8 weeks after treatment, the rats were euthanized, and cranial samples were harvested. To evaluate new bone formation within the defects, the samples were fixed in 4% paraformaldehyde for 24 h and then scanned via a micro-CT system as described above. Following the micro-CT scan, to assess bone formation, remodeling, and residual materials, the decalcified samples were dehydrated in a graded ethanol series, embedded in paraffin, and sectioned for histological evaluation, including H&E, MST, GST, and TRAP staining. At 4 weeks after implantation, ROS levels within the defect areas were evaluated with a dihydroethidium (DHE) fluorescence probe on frozen slices of collected bone tissues. In addition, some deparaffinized sections were subjected to immunohistochemical and immunofluorescence staining to investigate macrophage polarization and inflammation-related indicators, including iNOS, CD206, TNF-α, and IL-10. Furthermore, immunofluorescence staining was carried out to evaluate the expression of osteogenic and angiogenic marker proteins, including BMP-2 and VEGF. At 8 weeks after implantation, immunohistochemical staining of Runx2, OPN, OCN, and NFATc1 was conducted to analyze the expression of osteogenesis- and osteoclastogenesis-related markers within the defect areas. Immunofluorescence staining for the angiogenesis-related markers CD31 and α-SMA was performed to evaluate vascularization *in vivo*. Finally, to assess the potential toxicity of the implanted hydrogels *in vivo*, the major organs from the different groups, including the heart, liver, spleen, lung, and kidney, were collected for H&E staining. Serum biochemistry tests were conducted via commercial kits (Mindray, Shenzhen, China) and their accompanying automatic analyzers (Model BC-5380 and BS-420; Mindray, Shenzhen, China).

### Statistical analysis

The data are expressed as the mean ± standard deviation (SD) of three representative experiments. Statistical analysis in this research was carried out via one-way ANOVA with Tukey's test via Origin 2018 software (Origin Lab Corporation, USA). In all figures, the values were considered significant at p* or p^#^, corresponding to p < 0.05, and highly significant at p** or p^##^, corresponding to p < 0.01.

### Data availability

The data supporting this article are found within the text and the supplementary information file. Any additional data and the data that support the plots within this paper are available from the corresponding author upon reasonable request.

## Supplementary Material

Supplementary figures and table.

Supplementary movie 1.

Supplementary movie 2.

Supplementary movie 3.
