# Supplementary material for: Multifunctional injectable hydrogel system as a mild photothermal-assisted therapeutic platform for programmed regulation of inflammation and osteo-microenvironment for enhanced healing of diabetic bone defects in situ
Source: Theranostics. 2024 Oct 28;14(18):7140–98. doi: 10.7150/thno.102779 (PMC11610133; doi:10.7150/thno.102779)
Supplement: Supplementary file 1 — Supplementary figures and table. [file thnov14p7140s1.pdf]

Supporting Information

Multifunctional injectable hydrogel system as a mild photothermal-assisted therapeutic platform for programmed regulation of inflammation and osteo-microenvironment for enhanced healing of diabetic bone defects in situ

Yufan Zhu[#a], Huifan Liu[#b], Ping Wu[#c], Yun Chen[d], Zhouming Deng[*a], Lin Cai[*a], Minhao Wu[#a]

[a] Department of Spine Surgery and Musculoskeletal Tumor, Zhongnan Hospital of Wuhan University, 168 Donghu Street, Wuchang District, Wuhan 430071 Hubei, People's Republic of China

[b] Department of Anesthesiology, Research Centre of Anesthesiology and Critical Care Medicine, Zhongnan Hospital of Wuhan University, Wuhan, Hubei, China

[c] National Key laboratory of macromolecular drug development and manufacturing, School of Pharmaceutical Science, Wenzhou Medical University, Wenzhou, 325035, China

[d] Department of Biomedical Engineering and Hubei Province Key Laboratory of Allergy and Immune Related Disease, TaiKang Medical School (School of Basic Medicine Sciences), Wuhan University, Wuhan 430071, China

[#] These authors contributed equally to this work
* Correspondence should be addressed to:

Dr. Minhao Wu. Email: wuminhao1991@whu.edu.cn
Department of Spine Surgery and Musculoskeletal Tumor, Zhongnan Hospital of Wuhan University, 168 Donghu Street, Wuchang District, Wuhan 430071 Hubei, People's Republic of China

Dr. Lin Cai. Email: orthopedics@whu.edu.cn
Department of Spine Surgery and Musculoskeletal Tumor, Zhongnan Hospital of Wuhan University, 168 Donghu Street, Wuchang District, Wuhan 430071 Hubei, People's Republic of China

Dr. Zhouming Deng. Email: dengzhouming@whu.edu.cn
Department of Spine Surgery and Musculoskeletal Tumor, Zhongnan Hospital of Wuhan University, 168 Donghu Street, Wuchang District, Wuhan 430071 Hubei, People's Republic of China

The PDF file includes:

Supplementary figures and tables

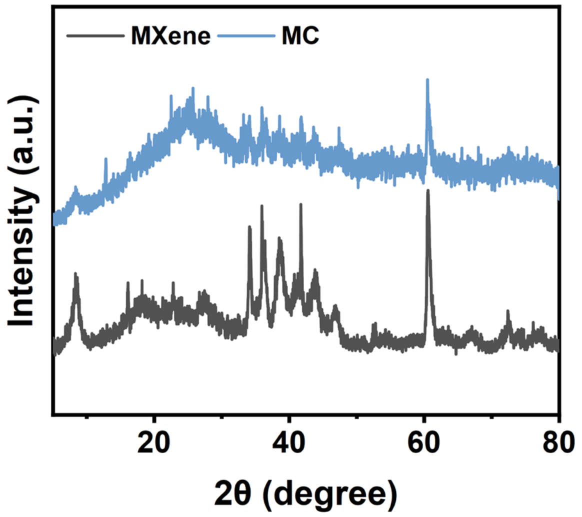

Figure S1. XRD spectra of MXene and MC nanosheets.

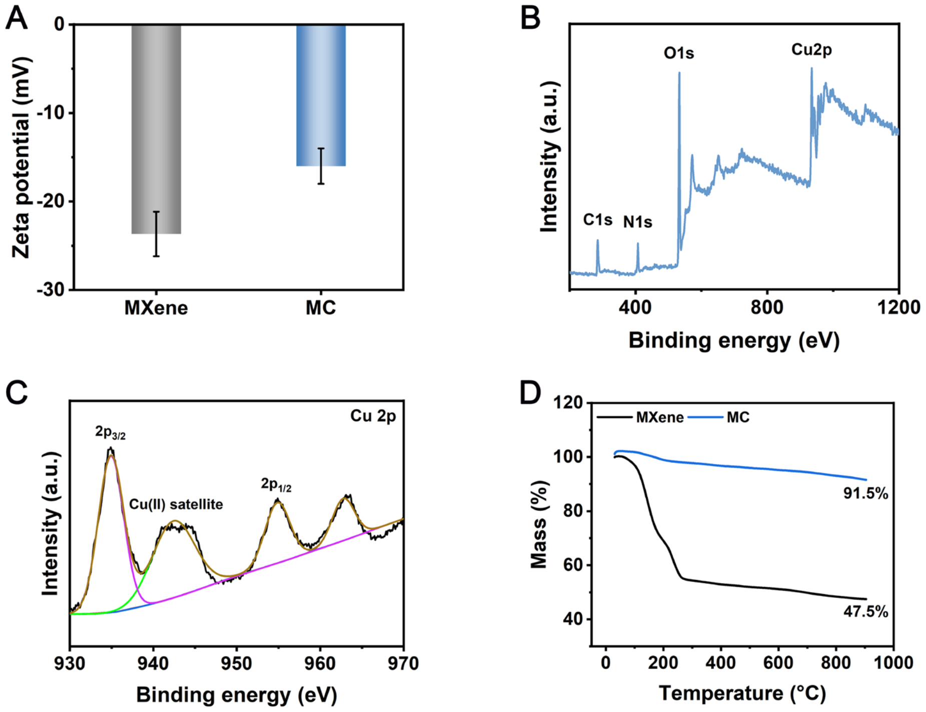

Figure S2. (A) Zeta potential of MXene and MC nanosheets. **(B)** XPS analysis of the MC nanosheets. **(C)** High-resolution Cu 2p XPS spectrum of MC nanosheets. **(D)** TG curves of MXene and MC nanosheets. Data are presented as the mean ± SD (n = 3).

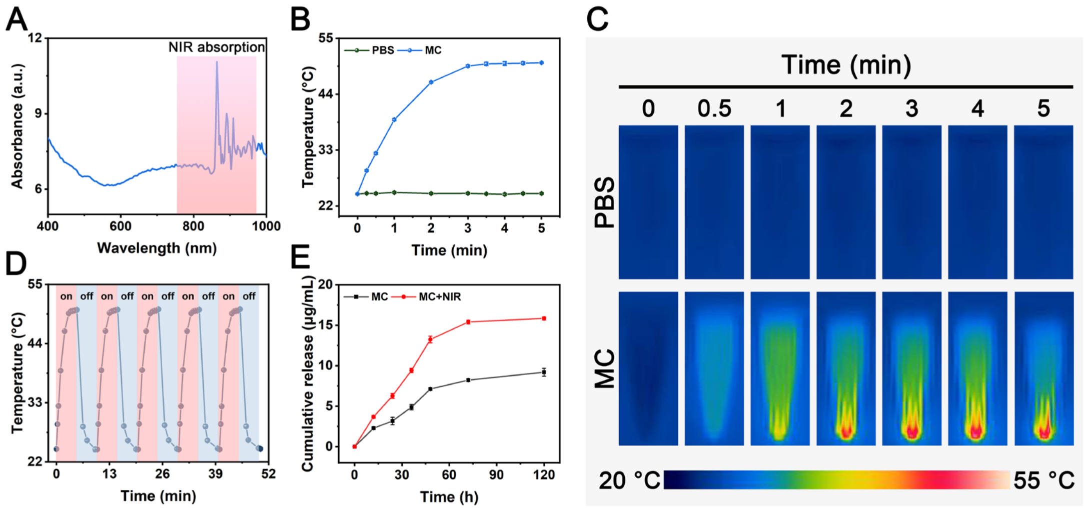

Figure S3. (A) UV-vis absorption spectra of the MC nanosheets. **(B-C)** Temperature curves and real-time infrared thermal images of MC nanosheets after 5 min of NIR irradiation. **(D)** Photothermal stability of MC nanosheets after five laser on/off cycles. **(E)** Release profiles of Cu^{2+} ions from MC nanosheets in PBS with or without NIR irradiation. Data are presented as the mean ± SD (n = 3).

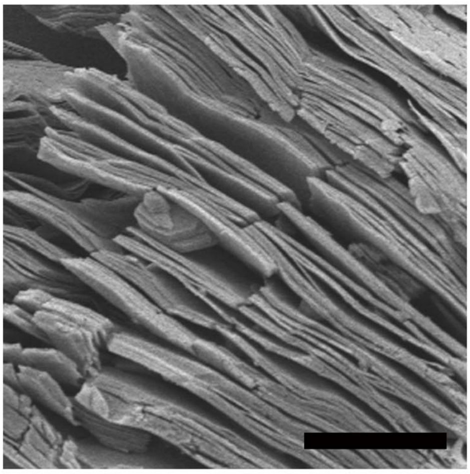

Figure S4. SEM images of MC nanosheets after NIR irradiation. Scale bar: 1 μm.

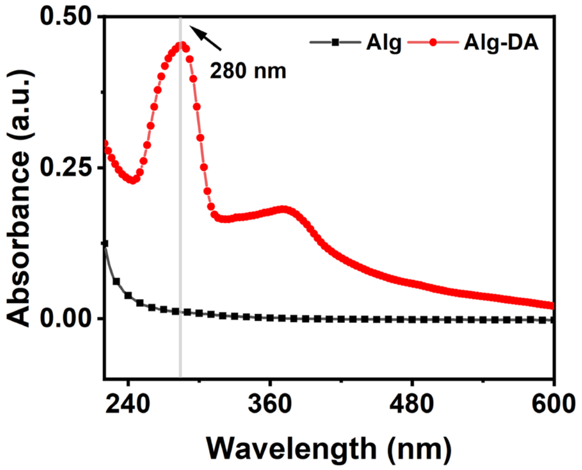

Figure S5. UV-vis absorption spectra of Alg and Alg-DA.

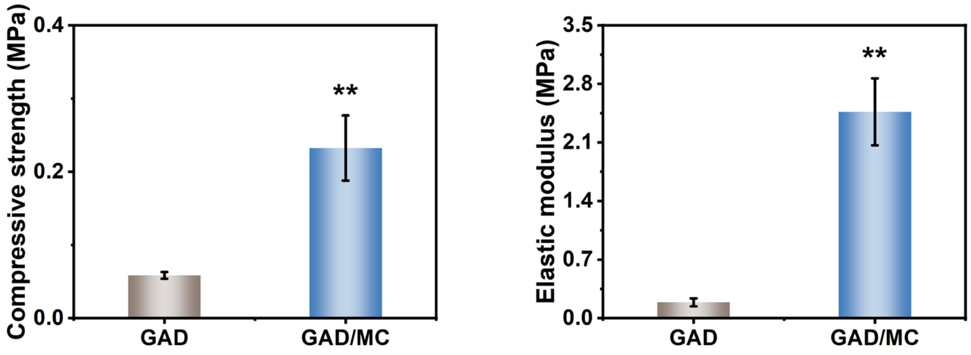

Figure S6. Compressive strength and elastic modulus of the hydrogels. Data are presented as the mean ± SD (n = 3). *P < 0.05 and **P < 0.01 indicate significant differences compared with the GAD group.

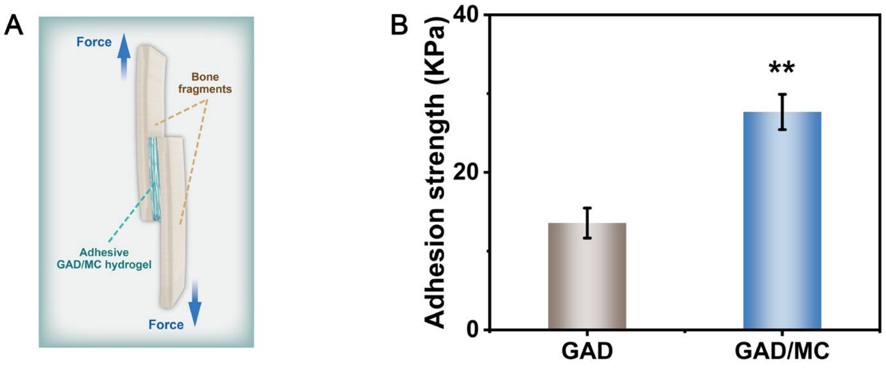

Figure S7. (A) Schematic illustration of the adhesive strength test. **(B)** Adhesive strength of hydrogels. Data are presented as the mean ± SD (n = 3). *P < 0.05 and **P < 0.01 indicate significant differences compared with the GAD group.

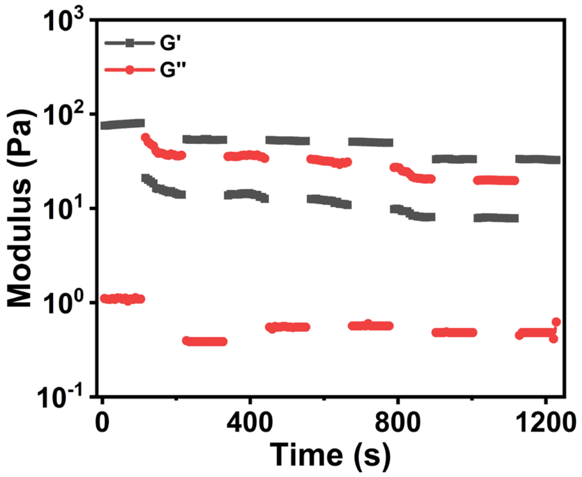

Figure S8. Rheological properties of the hydrogels dynamic rheological cycle test.

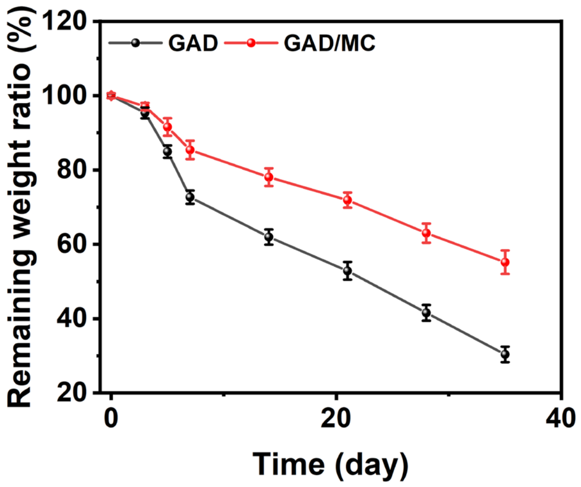

Figure S9. Degradation profiles of the hydrogels. Data are presented as the mean ± SD (n = 3).

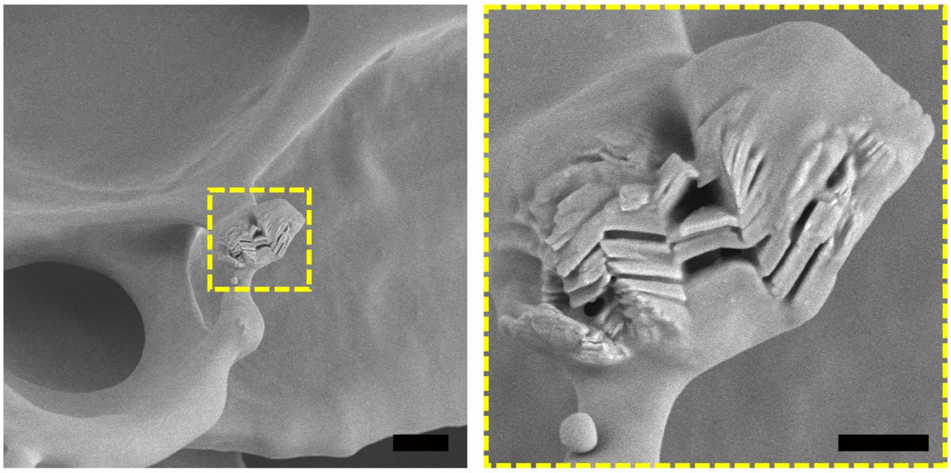

Figure S10. SEM image of the GAD/MC hydrogel after NIR irradiation. Scale bar: 3 μm (low-magnification SEM images), 1 μm (high-magnification SEM images).

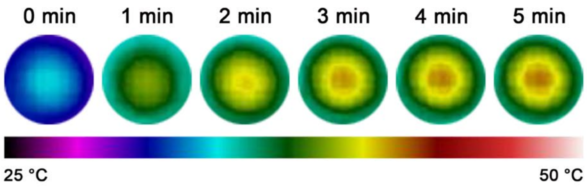

Figure S11. Infrared thermal images of the bacterial/hydrogel complex under NIR light (808 nm, 1.5 W/cm^2) for 5 min.

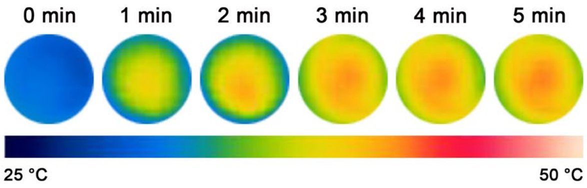

Figure S12. Infrared thermal images of the cell/hydrogel complex under NIR light (808 nm, 1.5 W/cm^2) for 5 min.

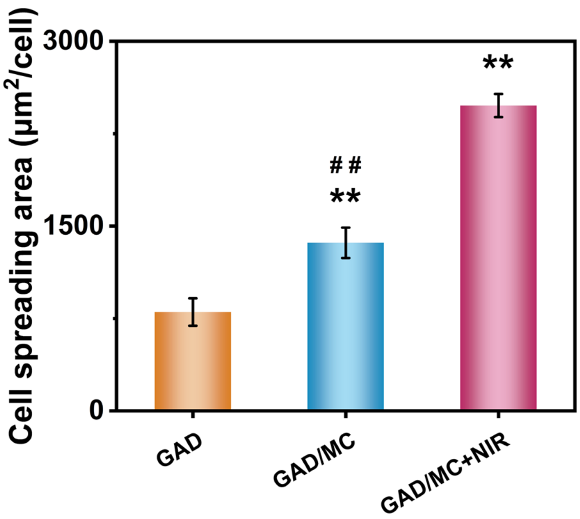

Figure S13. The quantification of the cell spreading area was based on F-actin staining. Data are presented as the mean ± SD (n = 3). *P < 0.05 and **P < 0.01 indicate significant differences compared with the GAD group. #P < 0.05 and # #P < 0.01 indicate significant differences compared with the GAD/MC+NIR group.

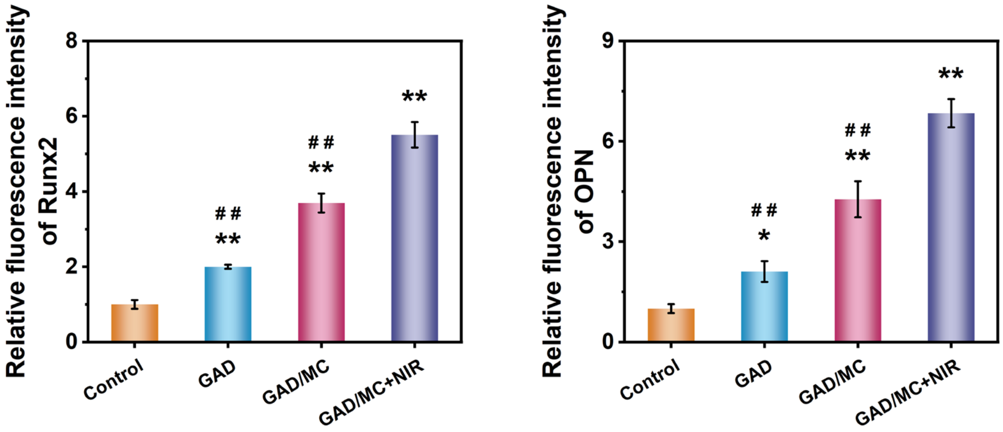

Figure S14. Quantitative analyses of the fluorescence intensity of Runx2 and OPN in different treatment groups. Data are presented as the mean ± SD (n = 3). *P < 0.05 and **P < 0.01 indicate significant differences compared with the control group. #P < 0.05 and # #P < 0.01 indicate significant differences compared with the GAD/MC+NIR group.

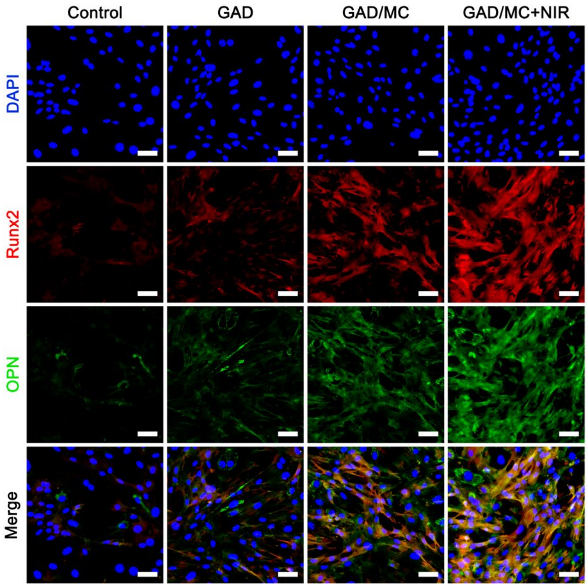

Figure S15. Representative immunohistochemical staining images of Runx2 and OPN in BMSCs after different treatments. Scale bar: 50 μm.

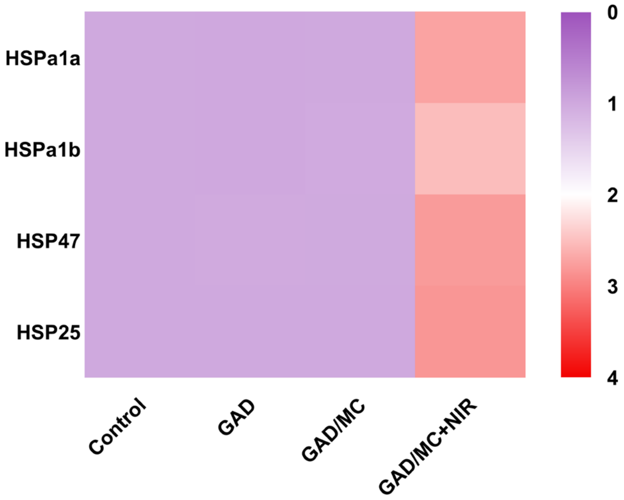

Figure S16. Relative mRNA expression of HSPs in MC3T3-E1 cells after different treatments. Data are presented as the mean ± SD (n = 3).

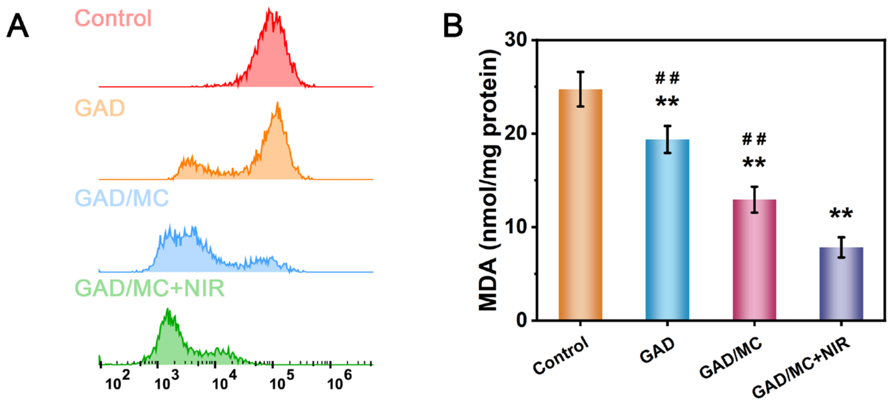

Figure S17. (A) Flow cytometry analysis of intracellular ROS levels in RAW264.7 cells under different treatment conditions. **(B)** MDA levels in the different treatment groups. Data are presented as the mean ± SD (n = 3). *P < 0.05 and **P < 0.01 indicate significant differences compared with the control group. $^{\#}$P < 0.05 and $^{\#\#}$P < 0.01 indicate significant differences compared with the GAD/MC+NIR group.

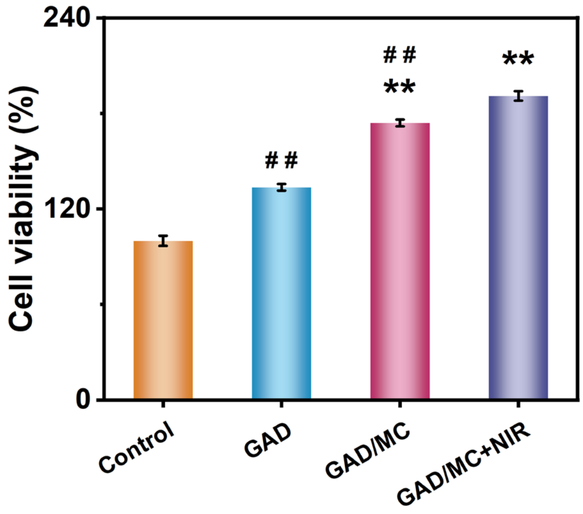

Figure S18. Cell viability of RAW264.7 cells after different treatments for 3 days. Data are presented as the mean ± SD (n = 3). *P < 0.05 and **P < 0.01 indicate significant differences compared with the control group. $^{\#}$P < 0.05 and $^{\#\#}$P < 0.01 indicate significant differences compared with the GAD/MC+NIR group.

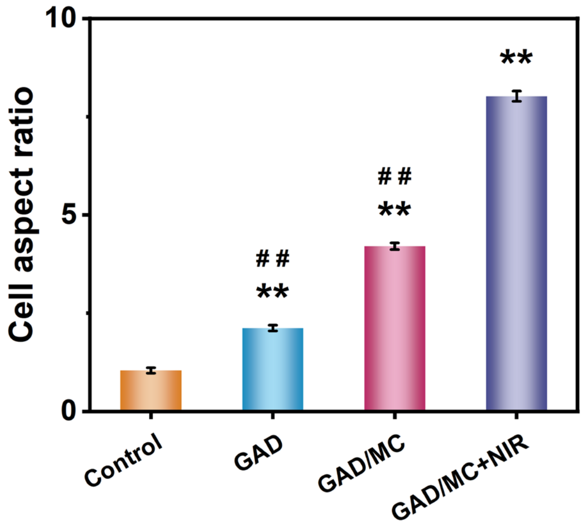

Figure S19. The cell aspect ratio was quantified based on F-actin staining. Data are presented as the mean ± SD (n = 3). *P < 0.05 and **P < 0.01 indicate significant differences compared with the control group. #P < 0.05 and # #P < 0.01 indicate significant differences compared with the GAD/MC+NIR group.

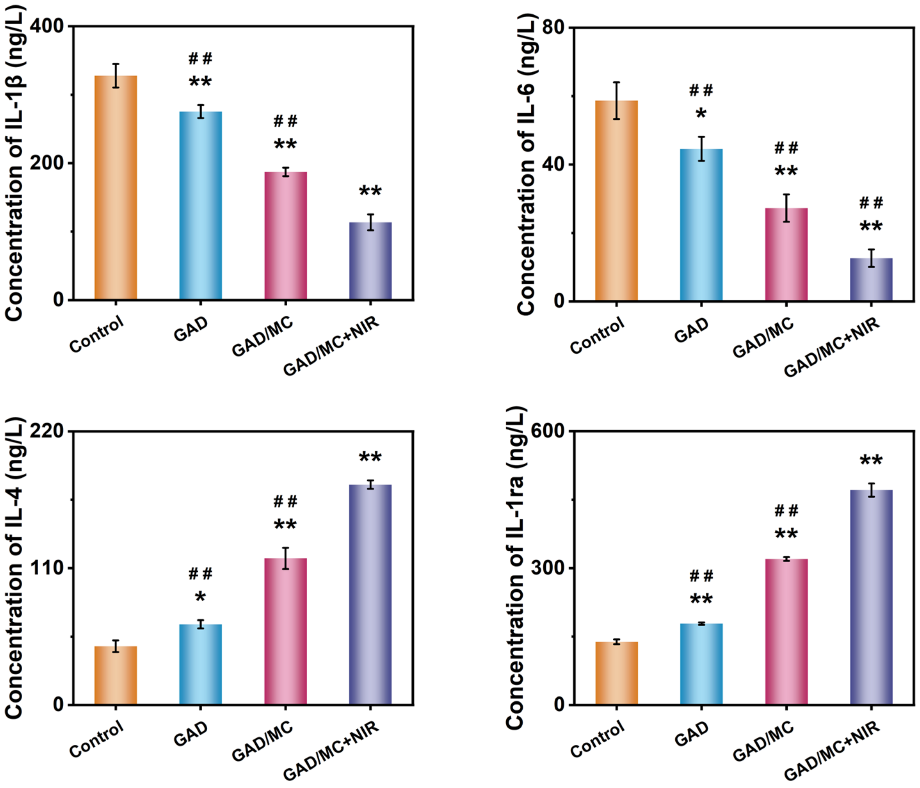

Figure S20. ELISA measurements of inflammatory factors secreted by macrophages after different treatments for 3 days. Data are presented as the mean ± SD (n = 3). *P < 0.05 and **P < 0.01 indicate significant differences compared with the control group. #P < 0.05 and # #P < 0.01 indicate significant differences compared with the GAD/MC+NIR group.

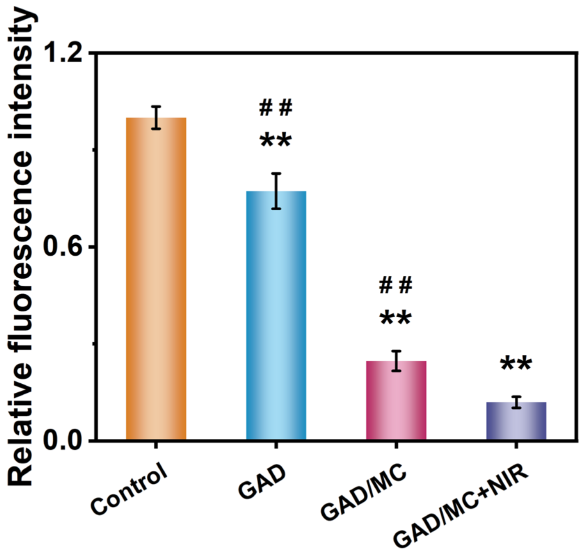

Figure S21. Quantitative analyses of the fluorescence intensity of ROS in different treatment groups. Data are presented as the mean ± SD (n = 3). *P < 0.05 and **P < 0.01 indicate significant differences compared with the control group. #P < 0.05 and ##P < 0.01 indicate significant differences compared with the GAD/MC+NIR group.

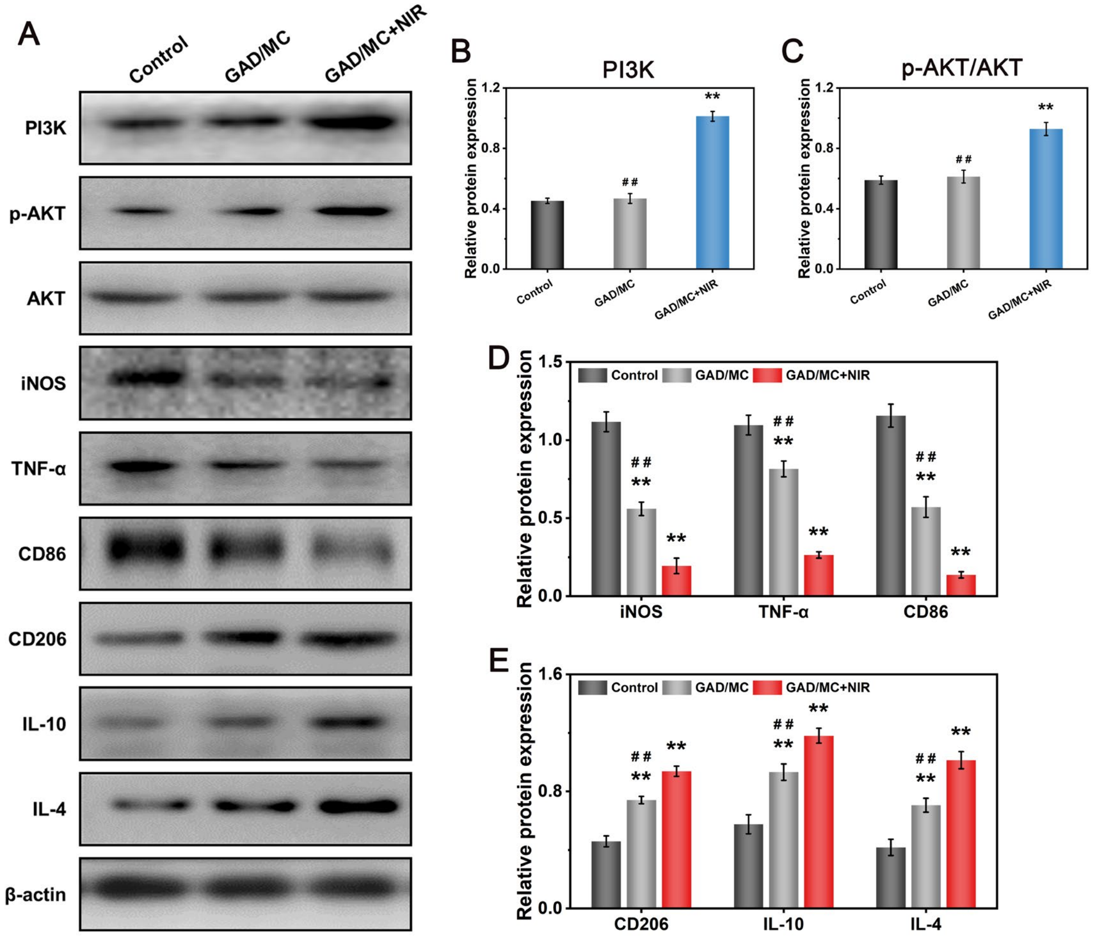

Figure S22. Western blot analysis and protein expression of PI3K, p-AKT, AKT, iNOS, TNF-α, CD86, CD206, IL-10, and IL-4 in RAW264.7 macrophages with or without periodic NIR irradiation. Data are presented as the mean ± SD (n = 3). *P < 0.05 and **P < 0.01 indicate significant differences compared with the control group. #P < 0.05 and ##P < 0.01 indicate significant differences compared with the GAD/MC+NIR group.

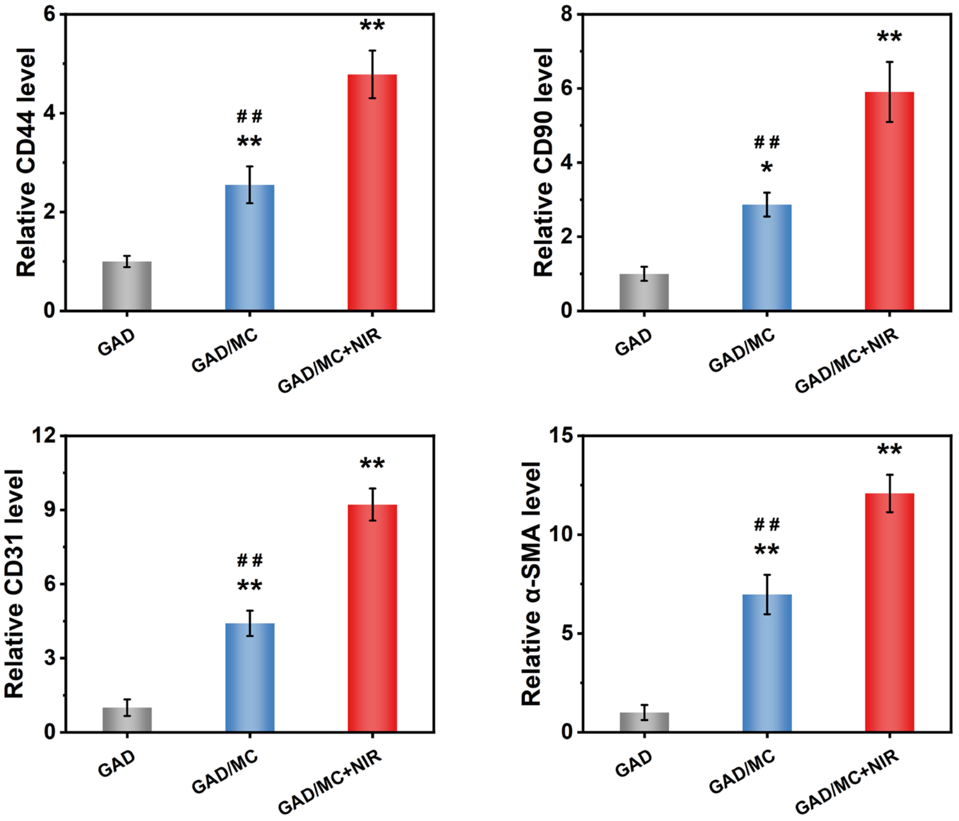

Figure S23. Quantitative analysis of immunohistochemical staining of hydrogel samples collected after different treatments. Data are presented as the mean ± SD (n = 3). *P < 0.05 and **P < 0.01 indicate significant differences compared with the GAD group. #P < 0.05 and ##P < 0.01 indicate significant differences compared with the GAD/MC+NIR group.

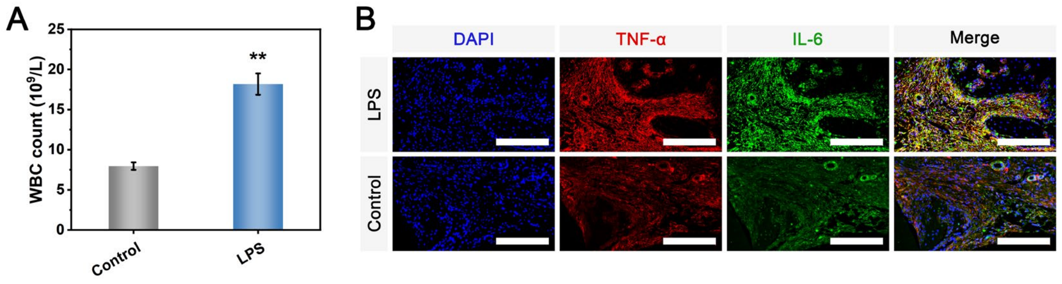

Figure S24. (A) WBC count of the rats with or without LPS injection. **(B)** Representative immunohistochemical staining images of TNF-α and IL-6 in the different treatment groups. Scale bar: 200 μm. Data are presented as the mean ± SD (n = 3). *P < 0.05 and **P < 0.01 indicate significant differences compared with the control group.

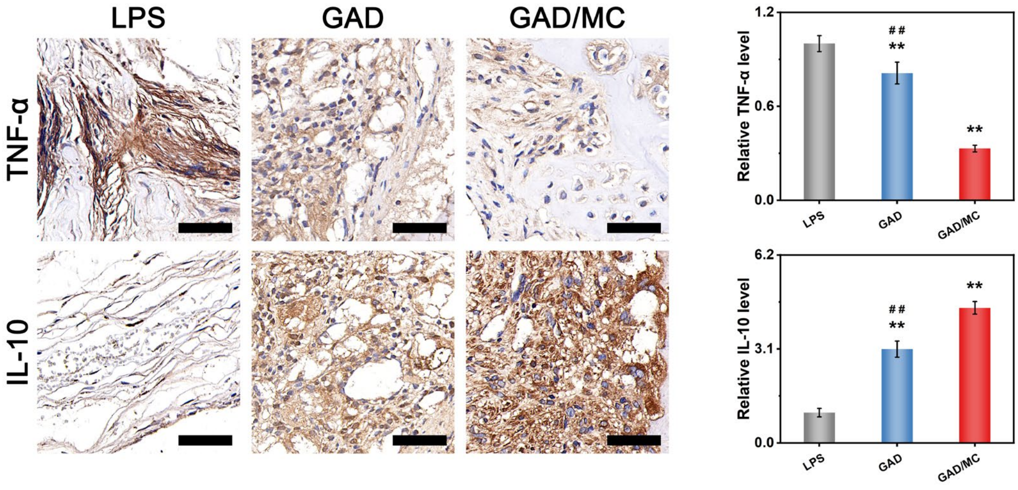

Figure S25. Representative immunohistochemical staining images and quantification of TNF-α and IL-10 in different treatment groups. Scale bar: 50 μm. Data are presented as the mean ± SD (n = 3). *P < 0.05 and **P < 0.01 indicate significant differences compared with the LPS group. #P < 0.05 and ##P < 0.01 indicate significant differences compared with the GAD/MC group.

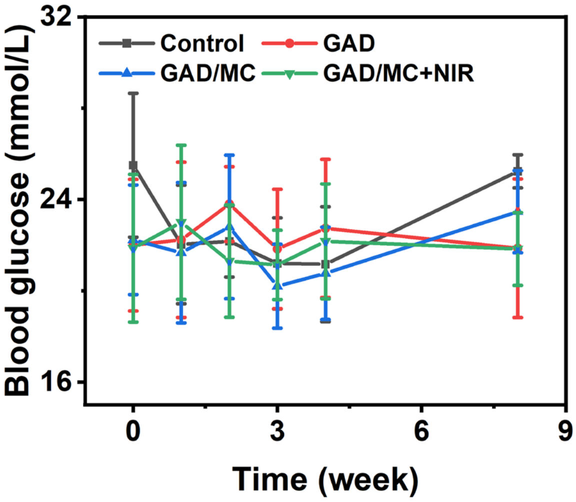

Figure S26. The blood glucose levels of the rats after induction with STZ. Data are presented as the mean ± SD (n = 3).

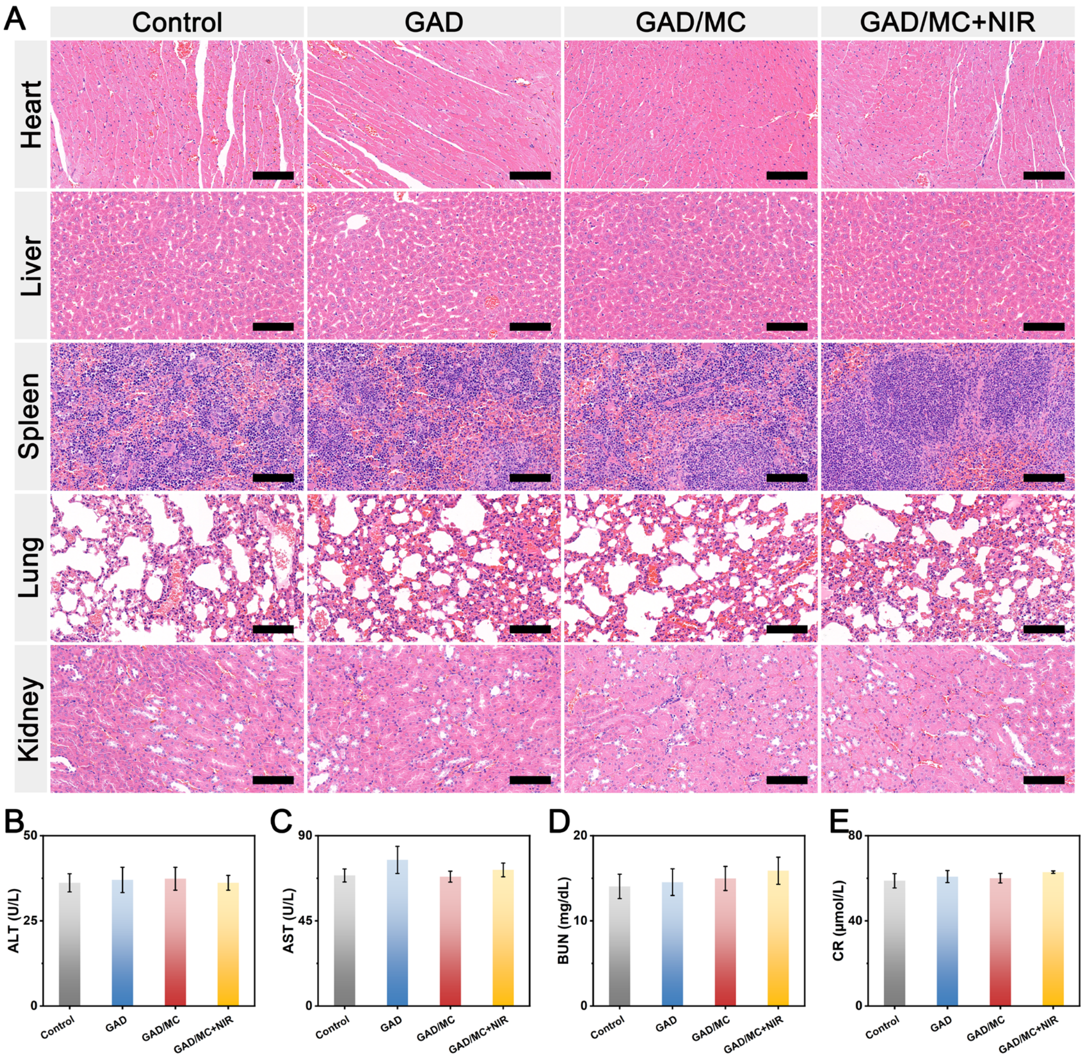

Figure S27. (A) Representative HE staining images of heart, liver, spleen, lung, and kidney after different treatments at 8 weeks. Scale bar: 50 μm. **(B)** Serological detection of ALT, AST, BUN, and CR levels. Data are presented as the mean ± SD (n = 3).

Table S1. Primer sequences used in qRT-PCR analysis.

Genes	Primers (F, forward; R, reverse; 5'-3')
Mouse-GADPH	F: TCAACGGCACAGTCAAGG
	R: TTAGTGGGGTCTCGCTCC
Mouse-Runx2	F: CATCCCAGTATGAGAGTAGGTGT
	R: GCTCAGATAGGAGGGGTAAGAC
Mouse-Col-1	F: CTGACTGGAAGAGCGGAGAG
	R: CGGCTGAGTAGGGAACACAC
Mouse-OPN	F: TCTGAGGGACTAACTACGACCAT
	R: TGGAAGAGTTTCTTGCTTAAAGTC

Mouse-OCN	F: TTCTGCTCACTCTGCTGACCC<br>R: CTGATAGCTCGTCACAAGCAGG
Mouse-TNF-α	F: CAGGCGGTGCCTATGTCTC<br>R: CGATCACCCCGAAGTTCAGTAG
Mouse-IL-6	F: GAGACCACTGGGGAGAATGC<br>R: TTGCCAGGTGGGTAAAGTGG
Mouse-iNOS	F: GAATCTTGGAGCGAGTTG<br>R: CCAGGAAGTAGGTGAGGG
Mouse-CD86	F: ATGGGCTCGTATGATTGT<br>R: TCTTAGGTTTCGGGTGAC
Mouse-IL-4	F: CATCCTGCTCTTCTTTCTC<br>R: TTCTCCTGTGACCTCGTT
Mouse-IL-10	F: TTTCAAACAAAGGACCAG<br>R: GGATCATTTCCGATAAGG
Mouse-Arg-1	F: AAGACAGCAGAGGAGGTG<br>R: AGTCAGTCCCTGGCTTA
Mouse-CD206	F: GCAAGTGATTTGGAGGCT<br>R: ATAGGAAACGGGAGAACC
Human-GAPDH	F: CATCATCCCTGCCTCTACTGG<br>R: GTGGGTGTCGCTGTTGAAGTC
Human-VEGF	F: TATGCGGATCAAACCTCACCA<br>R: CACAGGGATTTTTCTTGTCTTGCT
Human-HIF-1α	F: ATCCATGTGACCATGAGGAAAT<br>R: CTCGGCTAGTTAGGGTACACTT
Human-bFGF	F: AAGAGCGACCCTCACATCAA<br>R: GCCAGGTAACGGTTAGCACA
Human-Ang-1	F: CAGGAGGATGGTGGTTTG<br>R: GCCCTTTGAAGTAGTGCC

References

1. Wu, M.; Wang, Y.; Liu, H.; Chen, F.; Zhang, Y.; Wu, P.; Deng, Z.; Cai, L., *Mater Des* **2023,** *227*, 111705.
2. Wu, M.; Chen, F.; Liu, H.; Wu, P.; Yang, Z.; Zhang, Z.; Su, J.; Cai, L.; Zhang, Y., *Materials today. Bio* **2022,** *17*, 100458.
3. Qian, Y.; Zheng, Y.; Jin, J.; Wu, X.; Xu, K.; Dai, M.; Niu, Q.; Zheng, H.; He, X.; Shen, J., *Advanced materials (Deerfield Beach, Fla.)* **2022,** *34* (29), e2200521.

4. Li, S.; Yang, H.; Qu, X.; Qin, Y.; Liu, A.; Bao, G.; Huang, H.; Sun, C.; Dai, J.; Tan, J.; Shi, J.; Guan, Y.; Pan, W.; Gu, X.; Jia, B.; Wen, P.; Wang, X.; Zheng, Y., *Nature communications* **2024,** *15* (1), 3131.
5. Gong, J.; Ye, C.; Ran, J.; Xiong, X.; Fang, X.; Zhou, X.; Yi, Y.; Lu, X.; Wang, J.; Xie, C.; Liu, J., *ACS nano* **2023,** *17* (17), 16573-16586.
6. Wu, M.; Zhang, Y.; Wu, P.; Chen, F.; Yang, Z.; Zhang, S.; Xiao, L.; Cai, L.; Zhang, C.; Chen, Y.; Deng, Z., *NPJ Regenerative medicine* **2022,** *7* (1), 29.
7. Xue, Y.; Zhang, L.; Liu, F.; Kong, L.; Han, Y., *Nano Research* **2023,** *16* (2), 2905-2914.
8. Lee, N. H.; Kang, M. S.; Kim, T. H.; Yoon, D. S.; Mandakhbayar, N.; Jo, S. B.; Kim, H. S.; Knowles, J. C.; Lee, J. H.; Kim, H. W., *Biomaterials* **2021,** *276*, 121025.
9. Wu, M.; Liu, H.; Li, D.; Zhu, Y.; Wu, P.; Chen, Z.; Chen, F.; Chen, Y.; Deng, Z.; Cai, L., *Advanced science (Weinheim, Baden-Wurttemberg, Germany)* **2024,** *11* (2), e2304641.
10. Liu, W.; Zhao, H.; Zhang, C.; Xu, S.; Zhang, F.; Wei, L.; Zhu, F.; Chen, Y.; Chen, Y.; Huang, Y.; Xu, M.; He, Y.; Heng, B. C.; Zhang, J.; Shen, Y.; Zhang, X.; Huang, H.; Chen, L.; Deng, X., *Nature communications* **2023,** *14* (1), 4091.
11. Zhao, Y.; Wang, D.; Qian, T.; Zhang, J.; Li, Z.; Gong, Q.; Ren, X.; Zhao, Y., *ACS nano* **2023,** *17* (17), 16854-16869.
